# Higher Order Geometric Theory of Information and Heat Based on Poly-Symplectic Geometry of Souriau Lie Groups Thermodynamics and Their Contextures: The Bedrock for Lie Group Machine Learning

**DOI:** 10.3390/e20110840

**Published:** 2018-11-02

**Authors:** Frédéric Barbaresco

**Affiliations:** Department of Advanced Radar Concepts, Thales Land Air Systems, Voie Pierre-Gilles de Gennes, 91470 Limours, France; frederic.barbaresco@thalesgroup.com

**Keywords:** higher order thermodynamics, Lie groups thermodynamics, homogeneous manifold, poly-symplectic manifold, dynamical systems, non-equivariant cohomology, Lie group machine learning, Souriau-Fisher metric

## Abstract

We introduce poly-symplectic extension of Souriau Lie groups thermodynamics based on higher-order model of statistical physics introduced by Ingarden. This extended model could be used for small data analytics and machine learning on Lie groups. Souriau geometric theory of heat is well adapted to describe density of probability (maximum entropy Gibbs density) of data living on groups or on homogeneous manifolds. For small data analytics (rarified gases, sparse statistical surveys, …), the density of maximum entropy should consider higher order moments constraints (Gibbs density is not only defined by first moment but fluctuations request 2nd order and higher moments) as introduced by Ingarden. We use a poly-sympletic model introduced by Christian Günther, replacing the symplectic form by a vector-valued form. The poly-symplectic approach generalizes the Noether theorem, the existence of moment mappings, the Lie algebra structure of the space of currents, the (non-)equivariant cohomology and the classification of G-homogeneous systems. The formalism is covariant, i.e., no special coordinates or coordinate systems on the parameter space are used to construct the Hamiltonian equations. We underline the contextures of these models, and the process to build these generic structures. We also introduce a more synthetic Koszul definition of Fisher Metric, based on the Souriau model, that we name Souriau-Fisher metric. This Lie groups thermodynamics is the bedrock for Lie group machine learning providing a full covariant maximum entropy Gibbs density based on representation theory (symplectic structure of coadjoint orbits for Souriau non-equivariant model associated to a class of co-homology).

“*Inviter les savants géomètres à traiter nos problèmes avec le soucis de la commodité et de l’agrément: qu’ils écartent tout ce qui n’a rien à voir avec la pénétration de l’esprit, seule qualité dont nous faisons grand cas et que nous nous sommes proposé d’éprouver et de couronner*”

—Blaise Pascal—Deuxième Lettre sur la roulette, Paris, 19 Juillet 1658 [1]

“*Nous avons fait de la Dynamique un cas particulier de la Thermodynamique, une Science qui embrasse dans des principes communs tous les changements d’état des corps, aussi bien les changements de lieu que les changements de qualités physiques*”

—Pierre Duhem, Sur les équations générales de la Thermodynamique, 1891 [2]

“*Nous prenons le mot mouvement pour désigner non seulement un changement de position dans l’espace, mais encore un changement d’état quelconque, lors même qu’il ne serait accompagné d’aucun déplacement… De la sorte, le mot mouvement s’oppose non pas au mot repos, mais au mot équilibre*.”

—Pierre Duhem, Commentaire aux principes de la Thermodynamique, 1894 [3]

## 1. Introduction

These two Pierre Duhem’s citations (see [4] for English translations) make reference to Aristotle definition of “motion” (which can be found in *The Physics*) to designate not only a change of position in space, but also any change of state, even if not accompanied by any displacement. In this case, dynamics appears as a special case of General Thermodynamics [2,3,5], to describe in common principles all changes in the state of the body, both changes of place and changes in physical qualities. Making reference to Duhem’s “*Energetics*”, Stefano Bordini write in [6]: “*This theoretical design led Duhem to rediscover and reinterpret the tradition of Aristotle’s natural philosophy and Pascal’s epistemology … This outcome was surprising and clearly echoed the Aristotelian language and concept of motion as change and transformation: within the framework of Aristotelian natural philosophy, motion in the modern physical sense was actually a special case of the general concept of motion. The mathematisation of thermodynamics coincided with a generalisation of mechanics, and this generalisation led to an unexpected connection between modern mathematical physics and ancient natural philosophy*” (see [7,8] for more developments on the affiliation between Aristotle, Pascal and Duhem philosophies). This conceptual and epistemology point of view was enlightened 75 years later by Jean-Marie Souriau through the symplectic model of geometric mechanics applied to statistical mechanics and used to build a “Lie groups thermodynamics” of dynamical systems, where the Gibbs density is covariant with respect to the action of the Lie group on the system (dynamical groups as Galileo group). This Souriau theory is based on tools related to non-equivariant model associated to a class of co-homology and affine representation of Lie groups and Lie algebra (last approach was independently studied in mathematical domain by Koszul to characterize homogeneous convex cones geometry [9,10,11]). Duhem [12] and Souriau [13,14] also both studied how to extend Thermodynamics for a continuous media.

In this paper, we will explore and compare the joint geometric contextures shared in information theory (based on Koszul’s information geometry) and heat theory (based on Souriau’s Lie groups thermodynamics) to highlight their joint elementary structures. Classically, we address analogies between mathematical or physical models by comparing their “structures” defined as the arrangement of and relations between the parts or elements, or as the way in which the parts are arranged or organized. My personal concept of “contexture” is more general and phenomenological and could be defined as the act, process, or manner of weaving parts into a whole. We have then replaced the relations between objects by the act to build these relations. Based on Souriau’s general definition of entropy as the Legendre transform of the logarithm of generalized Laplace transform and symplectic structure associated to Lie group coadjoint orbits, we will see how geometric structures of information and heat theories are generated by these Souriau’s “generative processes”. We will extend theses contextures in the vector-valued case based on poly-symplectic model of higher order Souriau’s Lie groups thermodynamics.

In this paper, we identify the Riemanian metric introduced by Souriau based on co-homology, in the framework of “Lie groups thermodynamics” as an extension of classical Fisher metric introduced in information geometry. We have observed that Souriau metric preserves Fisher metric structure as the Hessian of the minus logarithm of a partition function, where the partition function is defined as a generalized Laplace transform on a convex cone. Souriau’s definition of Fisher metric extends the classical one in case of Lie groups or homogeneous manifolds. Souriau has developed “Lie groups thermodynamics” in the framework of homogeneous symplectic manifolds in geometric statistical mechanics for dynamical systems, but as observed by Souriau, these model equations are no longer linked to the symplectic manifold but only depend on the Lie group and the associated co-cycle.

This analogy with Fisher metric opens potential applications in machine learning, where the Fisher metric is used by information geometry, to define the “natural gradient” tool to improve ordinary stochastic gradient descent sensitivity to rescaling or changes of variable in parameter space [15,16,17,18,19,20,21,22]. In machine learning revised by natural gradient of information geometry, the ordinary gradient is designed to integrate the Fisher matrix. Amari has theoretically proved the asymptotic optimality of the natural gradient compared to classical gradient. With the Souriau approach, the Fisher metric could be extended, by Souriau-Fisher metric, to design natural gradients for data on homogeneous manifolds.

Information geometry has been derived from invariant geometrical structure involved in statistical inference. The Fisher metric defines a Riemannian metric as the Hessian of two dual potential functions, linked to dually coupled affine connections in a manifold of probability distributions. With the Souriau model, this structure is extended preserving the Legendre transform between two dual potential function parametrized in Lie algebra of the group acting transentively on the homogeneous manifold.

Classically, to optimize the parameter θ of a probabilistic model, based on a sequence of observations $y_{t}$, is an online gradient descent:

(1) $$\theta_{t}\leftarrow \theta_{t-1}-\eta_{t}\frac{\partial l_{t}{\left(y_{t}\right)}^{T}}{\partial \theta }\;$$

with learning rate $\eta_{t}$, and the loss function $l_{t}=-\log p\left(y_{t}/{\hat{y}}_{t}\right)$. This simple gradient descent has a first drawback of using the same non-adaptive learning rate for all parameter components, and a second drawback of non invariance with respect to parameter re-encoding inducing different learning rates. Amari has introduced the natural gradient to preserve this invariance to be insensitive to the characteristic scale of each parameter direction. The gradient descent could be corrected by $I{\left(\theta \right)}^{-1}$ where I is the Fisher information matrix with respect to parameter θ, given by:

(2) $$\begin{array}{l} I\left(\theta \right)=\left[g_{ij}\right]\\ \mathrm{with}\;g_{ij}={\left[-E_{y∼p(y/\theta )}\left[\frac{\partial^{2}\log p\left(y/\theta \right)}{\partial \theta_{i}\partial \theta_{j}}\right]\right]}_{ij}={\left[E_{y∼p(y/\theta )}\left[\frac{\partial \log p\left(y/\theta \right)}{\partial \theta_{i}}\frac{\partial \log p\left(y/\theta \right)}{\partial \theta_{j}}\right]\right]}_{ij} \end{array}$$

with natural gradient:

(3) $$\theta_{t}\leftarrow \theta_{t-1}-\eta_{t}I{\left(\theta \right)}^{-1}\frac{\partial l_{t}{\left(y_{t}\right)}^{T}}{\partial \theta }\;$$

Amari has proved that the Riemannian metric in an exponential family is the Fisher information matrix defined by:

(4) $$g_{ij}=-{\left[\frac{\partial^{2}\Phi }{\partial \theta_{i}\partial \theta_{j}}\right]}_{ij}\;\mathrm{with}\;\Phi (\theta )=-\log\int_{\mathbb{R}}e^{-\langle \theta ,y\rangle }dy\;$$

and the dual potential, the Shannon entropy, is given by the Legendre transform:

(5) $$S(\eta )=\langle \theta ,\eta \rangle -\Phi (\theta )\;\mathrm{with}\;\eta_{i}=\frac{\partial \Phi (\theta )}{\partial \theta_{i}}\;\mathrm{and}\;\theta_{i}=\frac{\partial S(\eta )}{\partial \eta_{i}}\;$$

In geometric statistical mechanics, Souriau has developed a “Lie groups thermodynamics” of dynamical systems where the (maximum entropy) Gibbs density is covariant with respect to the action of the Lie group. In the Souriau model, previous structures of information geometry are preserved:

(6) $$I(\beta )=-\frac{\partial^{2}\Phi }{\partial \beta^{2}}\;\mathrm{with}\;\Phi (\beta )=-\int_{M}e^{-\langle \beta ,U(\xi )\rangle }d\lambda \;$$

(7) $$S(Q)=\langle \beta ,Q\rangle -\Phi (\beta )\;\mathrm{with}\;Q=\frac{\partial \Phi (\beta )}{\partial \beta }\in g^{*}\;\mathrm{and}\;\beta =\frac{\partial S(Q)}{\partial Q}\in g\;$$

In the Souriau Lie groups thermodynamics model, β is a “geometric” (Planck) temperature, element of Lie algebra g of the group, and Q is a “geometric” heat, element of dual Lie algebra $g^{*}$ of the group. Souriau has proposed a Riemannian metric that we have identified as a generalization of the Fisher metric:

(8) $$I\left(\beta \right)=\left[g_{\beta }\right]\;\mathrm{with}\;g_{\beta }\left(\left[\beta ,Z_{1}\right],\left[\beta ,Z_{2}\right]\right)={\tilde{\Theta }}_{\beta }\left(Z_{1},\left[\beta ,Z_{2}\right]\right)\;$$

(9) $$\mathrm{with}\;{\tilde{\Theta }}_{\beta }\left(Z_{1},Z_{2}\right)=\tilde{\Theta }\left(Z_{1},Z_{2}\right)+\langle Q,ad_{Z_{1}}(Z_{2})\;\rangle \;\mathrm{where}\;ad_{Z_{1}}(Z_{2})=\left[Z_{1},Z_{2}\right]\;$$

The tensor $\tilde{\Theta }$ used to define this extended Fisher metric is defined by the moment map J(x), M (homogeneous symplectic manifold) to the dual Lie algebra $g^{*}$, given by:

(10) $$\tilde{\Theta }(X,Y)=J_{\left[X,Y\right]}-\left\{J_{X},J_{Y}\right\}\;\mathrm{with}\;J(x):\;M\to g^{*}\;\mathrm{such}\;\mathrm{that}\;J_{X}(x)=\langle J(x),X\rangle ,\;X\in g\;$$

This tensor $\tilde{\Theta }$ is also defined in tangent space of the cocycle $\theta \left(g\right)\in g^{*}$ (this cocycle appears due to the non-equivariance of the coadjoint operator $Ad_{g}^{*}$, action of the group on the dual lie algebra):

(11) $$Q\left(Ad_{g}(\beta )\right)=Ad_{g}^{*}(Q)+\theta \left(g\right)\;$$

(12) $$\begin{array}{lll} \tilde{\Theta }(X,Y): & g\times g\to ℜ\; & \mathrm{with}\;\Theta (X)=T_{e}\theta \left(X(e)\right)\\ & X,Y\mapsto \langle \Theta (X),Y\rangle & \end{array}$$

In Souriau’s Lie groups thermodynamics, the invariance by re-parameterization in information geometry has been replaced by invariance with respect to the action of the group. When an element of the group g acts on the element β∈g of the Lie algebra, given by adjoint operator $Ad_{g}$. Under the action of the group $Ad_{g}(\beta )$, the entropy S(Q) and the Fisher metric I(β) are invariant:

(13) $$\beta \in g\to Ad_{g}(\beta )\Rightarrow \left\{ \begin{array}{l} S\left[Q\left(Ad_{g}(\beta )\right)\right]=S\left(Q\right)\\ I\left[Ad_{g}(\beta )\right]=I\left(\beta \right) \end{array}\right.\;$$

In the case of small data analytics, we propose to parameterized the (maximum entropy) Gibbs density with higher order “geometric” temperature $\beta_{k}$ and higher order heat $Q_{k}$, that parameterized higher order entropy $S\left(Q_{1},\ldots ,Q_{n}\right)$ and dual potential function $\Phi (\beta_{1},\ldots ,\beta_{n})$:

(14) $$\begin{array}{l} S\left(Q_{1},\ldots ,Q_{n}\right)=\sum_{k=1}^{n}\langle \beta_{k},Q_{k}\rangle -\Phi (\beta_{1},\ldots ,\beta_{n})\\ \mathrm{with}\;\beta_{k}=\frac{\partial S(Q_{1},\ldots ,Q_{n})}{\partial Q_{k}}\;\mathrm{and}\;Q_{k}=\frac{\partial \Phi (\beta_{1},\ldots ,\beta_{n})}{\partial \beta_{k}}\\ \mathrm{where}\;\Phi (\beta_{1},\ldots ,\beta_{n})=-\log\int_{M}e^{-\sum_{k=1}^{n}\langle \beta_{k},U^{k}(\xi )\rangle }d\omega \end{array}$$

We will develop in the paper that the geometric approach of statistical thermodynamics, introduced by Souriau, offers an advantage over traditional formulations. Classical thermodynamics has been developed for static systems taking into accound only the time evolution, but in case of dynamical systems (e.g., a centrifuge system), this statistical physics is no longer valid because the Gibbs density (the density of the maximum entropy) is not covariant. In case of only time translation, what is preserved is only the energy, but for dynamical systems where a group is acting, invariants are given by components of the “moment map” (which is a geometrization of the Noether theorem providing invariants if there are symmetries). The “moment map” has been introduced in parallel by Kostant in mathematics and by Souriau in physics. Souriau has developed the non-equivariant case, and has applied it to statistical mechanics. The main advantages of “Lie groups thermodynamics” of dynamical systems, is that this statistical physics is a coordinate-free model preserving invariances with respect to the action of the (dynamical) Lie group acting on the system. We give in appendix the development of the centrifuge thermodynamics with classical approach given by Roger Balian, and prove that with the Souriau approach, the problem is solved by only applying Lie groups thermodunamics equations through moment map computation, where in classical case, we should consider additional terms related to all moments (energy, angular momentum, …) through additional Lagrange hyper-parameters, that corresponds to components of Souriau’s “geometric” (Planck) temperature.

Before developing all these models, and because this topic needs transverse knowledges of many concepts developed in different disciplines as statistical physics & thermodynamics, information geometry, symplectic mechanics and multi-symplectic geometry, we propose to the readers, in the preamble, to study the following books and papers:

- Introduction to Statistical Physics and Thermodynamics: [2,3,23,24,25,26]
- Introduction to Higher Order Thermodynamics: [27,28,29,30,31,32,33,34,35,36,37,38,39,40]
- Introduction to Information Geometry: [9,10,11,15,16,17,18,19,20,21,22,41,42]
- Introduction to Symplectic Mechanics: [43,44,45,46,47]
- Introduction to Multi-Symplectic Geometry: [48,49,50,51,52,53,54,55]

The geometric definition and extension of Fisher metric has been recently studied in the framework of quantum information geometry, but this community seems unaware of Souriau’s work on Lie groups thermodynamics for the study of statistical physics of dynamical systems based on symplectic geometry and c-homology tools in the 70s, and in particular the non-equivariant case developed by Souriau and Koszul. We can make reference to the following recent works on the symplectic formulation of the Fisher information theory [56,57,58,59].

The structure of the paper is the following:

- In Section 1, we introduce seminal idea on Symplectic geometry used in mechanics and in statistical mechanics, as introduced by Jean-Marie-Souriau during the 60s. From previous work of François Gallissot extending Cartan’s results on integral invariant (theorem on types of differential forms generating equations of movement of a material point invariant in the transformations of the Galilean group), we present the Lagrange 2-form and moment map elaborated by Souriau to build a geometric mechanics theory, where a dynamical system is then represented by a foliation of the evolution, determined by an antisymmetric covariant second order tensor. Souriau has applied this tool for mechanical statistics to build a thermodynamics of dynamical systems, where the classical notion of Gibbs canonical ensemble is extended for a homogeneous symplectic manifold on which a Lie group (dynamical group) has a symplectic action. In case of Galileo group, the symmetry is broken, and new “co-homological” Souriau relations should be verified in Lie algebra of the group.
- In Section 2, we synthetize results on higher order thermodynamics based on higher order temperatures and heats, as introduced by Ingarden and Jaworski for mesoscopic systems. This model is based on higher order maximum entropy Gibbs density definition constraining solution with respects to higher order moments.
- In Section 3, we develop “Lie groups thermodynamics” model, developed to describe Gibbs state for dynamical systems, where Souriau introduced the concept of co-adjoint action of a group on its momentum space that allows designing physical observables like energy, heat and momentum or moment as pure geometrical objects. The Souriau model then generalizes the Gibbs equilibrium state to all symplectic manifolds that have a dynamical group, with a “geometric” (Planck) temperature as an element of the Lie algebra and “geometric heat” as an element of the dual Lie algebra. We have observed that Souriau has introduced a symmetric tensor that is an extension of classical Fisher metric in information geometry. This new Fisher-Souriau metric is invariant with respect to the action of the group. These equations are universal, because they are not dependent on the symplectic manifold but only on the dynamical group and its associated two-cocycle. Souriau called it “Lie groups thermodynamics”.
- In Section 4, we give an extended Koszul study of Souriau’s non-equivariant model associated to a class of co-homology. Koszul has deepened the Souriau model, considering purely algebraic and geometric developments of geometric mechanics. Koszul has defined a skew symmetric bilinear form by a closed expression depending only on the cocycle and related to the Souriau antisymmetric bilinear map introduced previously in Section 3. This Koszul study of the moment map non-equivariance, and the existence of an affine action of G on g* is at the cornerstone of Souriau theory of Lie groups thermodynamics.
- In Section 5, at the step of the Souriau Lie groups thermodynamics presentation, we will introduce a generalized Souriau definition of entropy, as the Legendre transform of the logarithm of the Laplace transform, making the connection with information geometry. This definition is a general contexture that can be extended to highly abstract spaces preserving Legendre structure, if we are able to generalize the Laplace transform.
- In Section 6, we illustrate Souriau’s Lie groups thermodynamics for a centrifuge system. The main Souriau idea was to define the Gibbs states for one-parameter subgroups of the Galilean group, because he proved that the action of the full Galilean group on the space of motions of an isolated mechanical system is not related to any equilibrium Gibbs state (the open subset of the Lie algebra, associated to this Gibbs state, is empty).
- In Section 7, we have defined an higher-order model of Lie groups thermodynamics based on a poly-symplectic vector valued approach. This multi-symplectic extension, is based on a multi-valued one that preserve the notion of (poly-)moment map built by Günther based on an n-symplectic model. We replace the symplectic form of the Souriau model by a vector valued form that is called poly-symplectic. We consider the non-equivariance of poly-moment map by introducing poly-cocycle. We finally conclude with poly-symplectic definition extension of the Fisher-Souriau metric.
- In Section 8, we conclude with potential extension to Lie group machine learning.

To facilitate understanding of previous results, we add some additional complements:

- In Appendix A, we recall a synthesis of Günther’s poly-symplectic model with initial notation
- In Appendix B, we develop computation of the Fisher metric for multivariate Gaussian density, to establish links with Souriau’s Lie groups Gibbs density model.
- In Appendix C, we give more details on the Legendre transform, the basic tool of information geometry and Souriau Lie groups thermodynamics. More especially, we give a definition of the Legendre transform with projective geometry definition by Chasles as reciprocal polar with respect to a paraboloid.
- In Appendix D, we give solution of a centrifuge system thermodynamics, given by Roger Balian based on a classical approach, to make the link with the Souriau approach.
- In Appendix E, we recall the main proofs of Souriau’s Lie groups thermodynamics and its poly-symplectic extension.
- In Appendix F, we present another Souriau statistical physics model, developed for relativistic thermodynamics of continua, which preserves the Legendre transform, where temperature is given by a killing vector.

## 2. Seminal Idea of Symplectic Geometry in Mechanics and in Statistical Mechanics by Gallissot and Souriau

The symplectic structure has been introduced in mathematics much earlier than the word symplectic, in works of the French physicist Joseph Louis Lagrange (see paper on the slow changes of the orbital elements of planets in the solar system), who showed that this geometry is a fundamental tool in the mathematical model of any problem in mechanics. Jean-Marie Souriau has shown that Lagrange’s parentheses (nowdays called Lagranges bracket) are the components of the canonical symplectic 2-form on the manifold of motions of the mechanical system, in the chart of that manifold [60,61].

**Figure 1:**
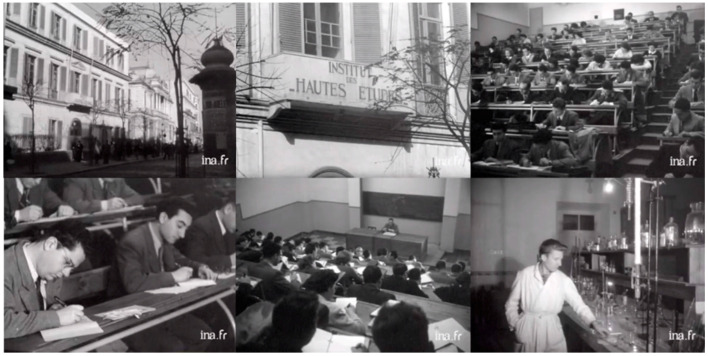
Institut des Hautes Etudes de Tunis, 8 rue de Rome where Souriau has developed his theory of Geometric Mechanics and Lie Groups Thermodynamics (http://www.ina.fr/video/AFE01000164).

Jean-Marie Souriau, graduated from ENS ULM 1942, was the nephew of the philosopher Etienne Souriau (graduated from ENS Ulm 1912, ranked 1st at aggregation, a collaborator of Gaston Bachelard in Paris Sorbonne University, PhD supervisor of Film Maker Eric Rohmer), author of “*Les Structures de l’oeuvre d’art*” and grandson of the philosopher Paul Souriau (graduated from ENS Ulm 1873), author of “*Esthétique du mouvement*” and a Latin thesis « De motus perceptione », who both have worked on “*aesthetics*”, and little nephew of literature historian Maurice Souriau, the editor of a critical version Blaise Pascal’s “*Pensées*” (awarded by 4 prices of Académie Française). The Souriau family, with Paul, Etienne and Jean-Marie were motivated to explore esthetical issues of “motion structures” (we could summarize by the triptych: the Esthetism of Motion of Paul Souriau, the Structure of Esthetism of Etienne Souriau and the Structure of Motion of Jean-Marie Souriau). Jean-Marie Souriau’s book “*Structure des Systems Dynamiques*” (SSD) was elaborated in Carthage and Marseille, where Souriau was installed with his wife Christiane Souriau-Hoebrecht. In 1952 Souriau found a position at Institut des Hautes Études de Tunis (8 rue de Rome, Tunis) (see Figure 1) and was back in Marseille in a position in 1958 at the Faculté des Sciences. The manuscript was given to the editor Dunod in 1969, but only edited in 1970 (2019 is the 50th birthday of this book and tributes will be given in 2 events FGSI’19 [62] and SOURIAU 2019 [63]). 

About the source of his book title, we are at the apogee or “acme” of the STRUCTURALISM in anthropology/sociology/linguistic/philosophy/ epistemology in France (Levi-Strauss, Barthes, Foucault, Althusser, Lacan, …). The word “structure” was in the air of the time, fashionable at the moment, circulating on all the lips as described by François Dosse in “*Histoire du structuralisme I & II*”. After his ONERA PhD Defence in 1953 (I have a copy of his PhD), his PhD supervisor André Lichnerowicz made one comment “*you have many anti-symmetrical forms in your calculations, you should be interested in symplectic structures*”.

As early as 1966, influenced by François Gallissot’s work, Souriau applied his theory of geometric mechanics to statistical mechanics, developed in Chapter IV of his book “*Structure of Dynamical Systems*” [43,64], what he called “Lie groups thermodynamics”. We have discovered that Souriau and Gallissot both attended the 1954 International Congress of Mathematicians (ICM’54) in Moscow. We could assume that they have discussed 1952 Gallissot’s paper introducing three types of differential forms generating equations of movement of a material point invariant in the transformations of the Galilean group and their links with Poincaré-Cartan integral invariant. This seminal work of Gallissot helped Souriau to formulate his new geometric mechanics and its extenxion to geometric statistical physics. Using Lagrange’s viewpoint, in Souriau statistical mechanics, a statistical state is a probability measure on the manifold of motions. As we can read in his book, Souriau was influenced by François Gallissot to introduce the Lagrange(-Souriau) 2-form.

In place of classical mechanical equations of a material point subjected to a force *F*, defined by its mass *m* and its position *r* at time *t*, the second order differential equations $m\frac{d^{2}r}{dt^{2}}=F\;$ is rewritten by a system of first order differential equations in phase space $\left(\begin{array}{c} r\\ v \end{array}\right)$:

(15) $$m\frac{dv}{dt}=F\;\mathrm{and}\;v=\frac{dr}{dt}\;$$

If the force *F* is derived from a potential *w*, we have classical equations:

(16) $$\begin{array}{l} \left\{ \begin{array}{l} L=\frac{1}{2}mv^{2}-w\;(\mathrm{Lagrangian})\\ H=\frac{1}{2}mv^{2}+w\;(\mathrm{Hamiltonian}) \end{array}\right.\\ \mathrm{with}\;A=\int_{t_{0}}^{t_{1}}Ldt \end{array}\;\mathrm{and}\;\mathrm{Hamilton}-\mathrm{Jacobi}\;\mathrm{equations}\;\left\{ \begin{array}{l} \frac{dq_{i}}{dt}=\frac{\partial H}{\partial p_{i}}\\ \frac{dp_{i}}{dt}=-\frac{\partial H}{\partial q_{i}} \end{array}\right.\;\mathrm{with}\;\left\{ \begin{array}{l} r=\left[\begin{array}{c} q_{1}\\ q_{2}\\ q_{3} \end{array}\right]\\ mv=\left[\begin{array}{c} p_{1}\\ p_{2}\\ p_{3} \end{array}\right] \end{array}\right.\;$$

**Figure 2:**
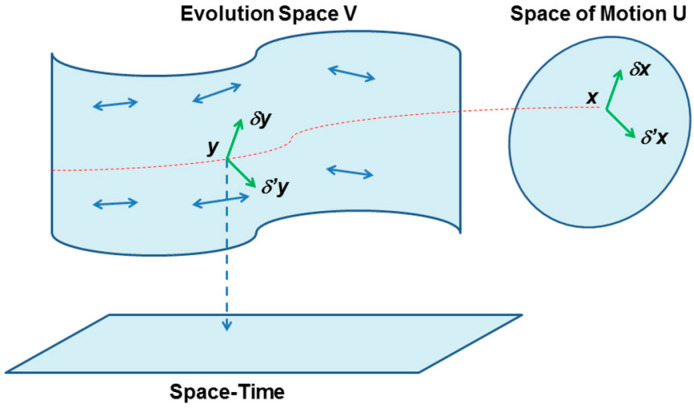
Evolution space V, Space of motions U and classical space time.

This idea of Lagrange, rediscovered by Souriau was to consider time *t* like the others variables. One should use then the 7-dimensional space *V* (evolution space) (see Figure 2):

(17) $$y=\left(\begin{array}{c} t\\ r\\ v \end{array}\right)\;$$

Classical system of first order differential equations in phase space can then be rewritten in evolution space *V* by the homogeneous form:

(18) $$\left\{ \begin{array}{l} m\delta v-F\delta t=0\\ \delta r-v\delta t=0 \end{array}\right.\;$$

At each point *y* of *V*, these equations define the tangent direction to the curve *x* described by the point *y* during the evolution of the system. These curves are the leaves (lines of force) of the field of directions defined by the equations of the homogeneous form, as defined for foliated manifolds. See [43], for more details on definition of the different derivatives used.

A dynamical system is then represented by a foliation of the evolution, where the foliation is determined by an antisymmetric covariant second order tensor, denoted by σ and called Lagrange-Souriau 2-form. The components of this tensor are expressions known as Lagrange brackets. σ is considered as a bilinear operator on tangent vectors of *V*. If we choose two such vectors:

(19) $$\delta y=\left(\begin{array}{c} \delta t\\ \delta r\\ \delta v \end{array}\right)\;\mathrm{and}\;\delta \prime y=\left(\begin{array}{c} \delta \prime t\\ \delta \prime r\\ \delta \prime v \end{array}\right)\;$$

σ associates to them an antisymmetric scalar product:

(20) σ(δy)(δ′y)=〈mδv−Fδt,δ′r−vδ′t〉−〈mδ′v−Fδ′t,δr−vδt〉 

In the Souriau-Lagrange model, σ is a 2-form on the evolution space *V*, and the differential equation of motion δy∈ε implies:

(21) σ(δy)(δ′y)=0 , ∀δ′y 

which can be written as:

(22) σ(δy)=0  or δy∈ker(σ) 

For study of this Souriau-Lagrange 2-form, readers should see the papers of Obădeanu [65,66,67].

Souriau has observed that this 2-form was introduced by Lagrange in a different language in his study of celestial mechanics in 1808. Souriau was also influenced by François Gallissot that used this 2-form in [68,69]. We will see in the following the Souriau’s “moment map *μ*” in dual Lie algebra of the group *G*, and the study of coadjoint orbits of *G*. For the definition of moment map, we make reference to [45]. Souriau has extended this model for thermodynamics. For this new phenomenological approach of mechanics, thermodynamics and information theory, we can give reference to Souriau introduction of his paper “*Quantique? Alors c’est géométrique*” [70] and a video of his talk [71]:

“*Plaçons-nous d’abord dans le cadre de la mécanique classique. Étudions un système mécanique isolé, non dissipatif—nous dirons brièvement une «chose». L’ensemble des mouvements de cette «chose» est une variété symplectique. Pourquoi? Il suffit de se reporter à la Mécanique Analytique de Lagrange (1811); l’espace des mouvements y est traité comme variété différentiable; les coordonnées covariantes et contravariantes de la forme symplectique y sont écrites (Ce sont les “parenthèses“ et “crochets“ de Lagrange). Évoquons maintenant la géométrie du 20 éme siècle. Soit G un groupe difféologique (par exemple un groupe de Lie); μ un moment de G (un moment, c’est une 1-forme invariante à gauche sur G); alors l’action du groupe sur μ engendre canoniquement un espace symplectique (ces groupes pourront avoir une dimension infinie). Présomption épistémologique: derrière chaque «chose» est caché un groupe G (sa “source“), et les mouvements de la «chose» sont simplement des moments de G (doublet latin mnémotechnique : momentum-movimentum). L’isolement de la «chose» indique alors que le groupe de Poincaré (respectivement de Galilée-Bargman) est inséré dans G; voilà l’origine des grandeurs conservées relativistes (respectivement classiques) associées à un mouvement x: elles constituent simplement le moment induit sur le groupe spacio-temporel par le moment-mouvement x.*” (In English: *Let’s put ourselves first in the framework of classical mechanics. Let’s study an isolated, non-dissipative mechanical system—we will briefly say a “thing”. The set of movements of this “thing” is a symplectic manifold. Why? It is enough to refer to the Analytical Mechanics of Lagrange (1811); the space of movements is treated as a differentiable manifold; the covariant and contravariant coordinates of the symplectic form are written there (these are the “parentheses” and “brackets” of Lagrange). Let’s now talk about the geometry of the 20th century. Let G be a diffeological group (for example a Lie group); μ a moment of G (a moment is a left invariant 1-form on G); then the action of the group on μ canonically generates a symplectic space (these groups can have an infinite dimension). Epistemological presumption: behind each “thing” is hidden a group G (its “source”), and the movements of the “thing” are simply moments of G (mnemonic Latin doublet: momentum-movimentum). The isolation of the “thing” then indicates that the group of Poincaré (respectively Galileo-Bargman) is inserted in G; here is the origin of the relativistic (respectively classical) conserved magnitudes associated with a movement x: they simply constitute the moment induced on the spacio-temporal group by the moment-motion x*.)

“*Il y a un théorème qui remonte au XXème siècle. Si on prend une orbite coadjointe d’un groupe de Lie, elle est pourvue d’une structure symplectique. Voici un algorithme pour produire des variétés symplectiques: prendre des orbites coadjointes d’un groupe. Donc cela laisse penser que derrière cette structure symplectique de Lagrange, il y avait un groupe caché. Prenons le mouvement classique d’un moment du groupe, alors ce groupe est très «gros» pour avoir tout le système solaire. Mais dans ce groupe est inclus le groupe de Galilée, et tout moment d’un groupe engendre des moments d’un sous-groupe. On va retrouver comme cela les moments du groupe de Galilée, et si on veut de la mécanique relativiste, cela va être du groupe de Poincaré. En fait avec le groupe de Galilée, il y a un petit problème, ce ne sont pas les moments du groupe de Galilée qu’on utilise, ce sont les moments d’une extension centrale du groupe de Galilée, qui s’appelle le groupe de Bargman, et qui est de dimension 11. C’est à cause de cette extension, qu’il y a cette fameuse constante arbitraire figurant dans l’énergie. Par contre quand on fait de la relativité restreinte, on prend le groupe de Poincaré et il n’y a plus de problèmes car parmi les moments il y a la masse et l’énergie c’est mc^2^. Donc le groupe de dimension 11 est un artéfact qui disparait, quand on fait de la relativité restreinte*.” (In Engish: *There is a theorem dating back to the twentieth century. If we take a coadjoint orbit of a Lie group, it is provided with a symplectic structure. Here is an algorithm to produce symplectic manifolds: take coadjoint orbits from a group. So it suggests that behind this symplectic structure of Lagrange, there was a hidden group. Take the classic movement of a moment of the group, so this group is very “big” to have the whole solar system. But in this group is included the Galileo group, and any moment of a group generates moments of a subgroup. We will find like that the moments of the group of Galileo, and if we want relativistic mechanics, it will be Poincaré group. In fact with Galileo group, there is a small problem, it is not the moments of the Galileo group that are used, it is the moments of a central extension of the Galileo group, which is called the Bargman group, and that is of dimension 11. It is because of this extension, that there is this famous arbitrary constant appearing in the energy. On the other hand, when we do special relativity, we take Poincaré group and there are no more problems because among the moments there is the mass and the energy is mc^2^. So the 11-dimensional group is an artifact that disappears, when we do special relativity*.)

François Gallissot has observed that in his famous lessons on integral invariants, Elie Cartan has shown that all the properties of the differential equations of the dynamics of holonomic systems result from the existence of the integral invariant:

(23) $$\int \omega \;\mathrm{with}\;\omega =\sum_{i}p_{i}dq_{i}-Hdt\;$$

Thus every holonomic system whose forces derive from a force function is associated to a form ω, the equations of motion being the characteristics of the exterior form dω. Around 1950, the theory of exterior forms on differentiable manifolds has been established on new foundations under the influence of topologists. The question was then to wonder:

- if classical mechanics cannot benefit from these models by placing an exterior form of degree two at its base
- if thanks to the notion of manifold, the notion of connection cannot be introduced in a more natural way
- if the paradoxal indeterminations/impossibilities in the Lagrangian framework could be explained more clearly
- if the problem of integration of equations of motion could be enlightened, generated by a form Ω of degree two.

To reach these various objectives, Gallissot has resumed first the study of the logical bases on which the Galilean mechanics is built. He thus shown that when it is proposed to find generating forms of the equations of motion of a material invariant point in the transformations of the Galilean group, the most interesting form is an exterior form of degree two defined on a variety $E^{3}\times E\times T$ ($E^{3}$ Euclidean space, T temporal). Gallissot had shown that any holonomic parametric system with n degrees of freedom is associated with a form Ω of degree *2n* defined on a differentiable manifold whose characteristics are the equations of the movement. This form is expressed by means of 2*n* Pfaff forms and by *dt*, the Hamiltonian form being a simple special case. He gave a summary of how we can get rid of the servitude of coordinates in the study of dynamical systems and the important role played by the operator i() antiderivative introduced by Cartan, the characteristic field *E* of the form Ω being defined by the relation i(E)Ω=0. Gallissot has then introduced the following theorem:

**Theorem** **1.** *There are three types of differential forms generating equations of movement of a material point invariant in the transformations of the Galilean group*:

(24) $$\begin{array}{l} A:\;\left\{ \begin{array}{l} s=\frac{1}{2m}\sum_{i=1}^{3}{\left(mdv_{i}-F_{i}dt\right)}^{2}\\ e=\frac{m}{2}\sum_{j=1}^{3}{\left(dx_{j}-v_{j}dt\right)}^{2} \end{array}\right.\\ B:\;f=\sum_{1}^{3}\delta_{ij}\left(dx_{i}-v_{i}dt\right)\left(mdv_{j}-F_{j}dt\right)\;\mathrm{with}\;\delta_{ij}\;\mathrm{krönecker}\;\mathrm{symbol}\\ C:\;\omega =\sum_{1}^{3}\delta_{ij}\left(mdv_{i}-F_{i}dt\right)\wedge \left(dx_{j}-v_{j}dt\right) \end{array}$$

If we consider the last form “*C*”:

(25) $$\omega =\sum_{1}^{3}\delta_{ij}\left(mdv_{i}-F_{i}dt\right)\wedge \left(dx_{j}-v_{j}dt\right)=m\delta_{ij}dv_{i}\wedge dx_{j}-m\delta_{ij}v_{i}dv_{j}\wedge dt+\delta_{ij}F_{i}dx_{j}\wedge dt\;$$

dω=0 constraints Pfaff form $\delta_{ij}F_{i}dx_{j}$ to be closed, and to reduce the differential of function U:

(26) $$\omega =m\delta_{ij}dv_{i}\wedge dx_{j}-dH\wedge dt\;$$

(27) $$\mathrm{with}\;H=T-U\;\mathrm{and}\;T=\frac{1}{2}\sum_{i=1}^{3}m{\left(v_{i}\right)}^{2}\;$$

It proves that the exterior derivative of ω is:

(28) $$d\omega =\sum_{i=1}^{3}mv_{i}dx_{j}-Hdt\;$$

The form $\omega^{*}=d\omega$ generates Elie Cartan integral invariant.

In Chapter IV of his book, Souriau applied this model based on symplectic geometry for statistical mechanics. Souriau observed that Gibbs equilibrium is not covariant with respect to dynamic groups of physics. To solve this breaking of symmetry, Souriau introduced a new “geometric theory of heat” where the equilibrium states are indexed by a parameter β with values in the Lie algebra of the group, generalizing the Gibbs equilibrium states, where β plays the role of a geometric (Planck) temperature. Souriau observed that the group of time translations of the classical thermodynamics is not a normal subgroup of the Galileo group, proving that if a dynamical system is conservative in an inertial reference frame, it need not be conservative in another. Based on this fact, Souriau generalized the formulation of the Gibbs principle to become compatible with Galileo’s relativity in classical mechanics and with Poincaré relativity in relativistic mechanics. The maximum entropy principle is preserved, and the Gibbs density is given by the density of maximum entropy (among the equilibrium states for which the average value of the energy takes a prescribed value, the Gibbs measures are those which have the largest entropy), but with a new principle “*If a dynamical system is invariant under a Lie subgroup G’ of the Galileo group, then the natural equilibria of the system forms the Gibbs ensemble of the dynamical group G’*”. The classical notion of Gibbs canonical ensemble is extended for a homogneous symplectic manifold on which a Lie group (dynamic group) has a symplectic action. In case of a Galileo group, the symmetry is broken, and new “cohomological” relations should be verified in Lie algebra of the group. A natural equilibrium state will thus be characterized by an element of the Lie algebra of the Lie group, determining the equilibrium temperature β. The entropy s(Q), parametrized by Q the geometric heat (mean of energy U, element of the dual Lie algebra) is defined by the Legendre transform of the Massieu potential given by Φ(β), parametrized by β (Φ(β) is the minus logarithm of the partition function $\psi_{\Omega }\left(\beta \right)$):

(29) $$s\left(Q\right)=\langle \beta ,Q\rangle -\Phi (\beta )\;\mathrm{with}\;\Phi (\beta )=-\log\int_{M}e^{-\langle \beta ,U(\xi )\rangle }d\omega \;,\;Q=\frac{\partial \Phi }{\partial \beta }\in g^{*}\;\mathrm{and}\;\beta =\frac{\partial s}{\partial Q}\in g\;$$

Souriau has proposed to study the statistical mechanics from the new point of view of symplectic geometry, completing the work of Poincaré and Cartan on integral invariant, reinventing the Lagrangian symplectic form in place of classical variational formulation and geometrizing the Noether theorem with a moment map as new conserved quantities. Firstly, Souriau Lie groups thermodynamics gives geometrical status to the (Planck) temperature and the entropy with a new general definition of the Fisher Metric. Secondly, Souriau’s relativistic thermodynamics of continua provides a geometrization of the smecond principle by the permanence of the entropy current, whose flux has positive divergence [13,14,72,73,74]. This 2nd model of Souriau’s thermodynamics is described in the Appendix. Other authors have studied this relativistic thermodynamics of continua [75,76,77,78,79,80,81,82].

If some works have been done from the 80s by Ingarden [83,84] and Mrugala [85,86,87,88,89] and Arnold [90] to give a geometric structures to thermodynamics, Souriau’s Lie groups thermodynamics was ignored for more than 50 years until recently recovered in [23,91].

## 3. Higher Order Thermodynamics Based on Higher Order Temperatures

We will generalize Souriau’s theory [43,64], reconsidered in [23] and with links to information geometry in [91], in the framework of higher order thermodynamics as introduced by Ingarden [29,30,31] and Jaworski [32,33,34,35] for mesoscopic systems. We can make also reference to other publications of Ingarden [36,37,38,39,40], Jaworsky [92,93,94] and Nakagomi [95] on higher order thermodynamics. The Gibbs canonical state results from the maximum entropy principle when the statistical mean value of the energy is supposed to be known. A Polish school has studied the maximum entropy inference with higher-order moments of energy (when not only mean values but also statistical moments of higher order of some physical quantities are taken into account). Ingarden in 1963 and Jaworski in 1981 have introduced the concept of second and higher-order temperatures, by assuming a distribution function which includes information not only on the average of the energy but also on higher-order moments, in particular 2nd moment related to fluctuations. This case should be considered in situations where fluctuations are not negligible, such as near phase transitions or critical points, in metastable states in systems with a small number of degrees of freedom. Ingarden’s idea is that if we can measure more details, such as the first *n* cumulants of the energy, we can then introduce *n* high-order temperature, as the Lagrange multipliers when we maximize the entropy with respect to these values:

(30) $$P_{\left(\beta_{1},\beta_{2}\right)}=\frac{1}{Z\left(\beta_{1},\beta_{2}\right)}e^{-\beta_{1}.H-\beta_{2}{\left(H-U\right)}^{2}}=e^{\beta_{0}-\beta_{1}.H-\beta_{2}{\left(H-U\right)}^{2}}\;$$

Ingarden proposed that if we can measure the second cumulant of the energy (the fluctuation of the energy), the equilibrium state is not the canonical state, but would need two temperatures. Ingarden argues that for a macroscopic system there is very little difference between the two states, and that we would need a mesoscopic or microscopic system to be able to detect the higher temperature. Jaworski [27,28] has shown that the contribution to the total entropy, arising from the extra information corresponding to the higher-order moments, is *o*(*N*), when *N* tends to infinity and *N/V* ratio is constant, with *N* the number of particles and *V* the volume. The main result of Jaworski is that from a purely thermodynamic point of view, the information corresponding to the higher-order moments of extensive physical quantities is not essential and can be neglected in the maximum entropy procedure. Jaworski showed that the maximum entropy inference has a certain stability property with respect to information corresponding to higher order moments of extensive quantities. It can serve as an argument in favor of the maximum entropy method in statistical physics and to understand better why these methods are successful. Streater [96] has prefered to say that the states with generalized temperatures are not in equilibrium, assuming that the final state, at large times, will be the canonical or grand canonical state depending on mixing properties. Streater [96] intends that this occur even for a mesoscopic system, such as a few atoms, adding that his approach is equivalent to Ingarden model if the relaxation time from the state with generalized temperatures to the final equilibrium is very long.

Some examples of higher order maximum Entropy are given by Ingarden:

● *1st Example of Higher Oder Maximum Entropy Density*:

Density of maximum Entropy

(31) $$S(P)=-\int_{-\infty }^{+\infty }P(x)\log P(x)dx\;$$

under the constraints:

(32) $$P(x)\geq 0,\;\int_{-\infty }^{+\infty }P(x)dx=1\;\mathrm{and}\;E\left(x^{2n}\right)=\int_{-\infty }^{+\infty }x^{2n}P(x)dx=\sigma^{2n}\;$$

is given by:

(33) $$P(x)=\frac{1}{2{\left(2n\right)}^{\frac{1}{2n}}\sigma .\Gamma \left(1+1/2n\right)}\exp\left(-\frac{x^{2n}}{2n\sigma^{2n}}\right)=f_{n}(x)\;$$

with the following parameters:

(34) $$\beta_{n}=\frac{1}{2n\sigma^{2n}},\;Z\left(\beta_{n}\right)=\frac{2\Gamma \left(1+1/2n\right)}{\beta_{n}^{1/2n}},\;S(P)=\log Z\left(\beta_{n}\right)+\frac{1}{2n}\;$$

where:

(35) $$E\left(x^{2k-1}\right)=0\;\mathrm{and}\;-\frac{\partial \log Z\left(\beta_{k}\right)}{\partial \beta_{k}}=\sigma^{2k}=E\left(x^{2k}\right)=\frac{{\left(2n\right)}^{k/n}\sigma^{2k}\Gamma \left(1+\left(2k+1\right)/2n\right)}{\left(2k+1\right)\Gamma \left(1+1/2n\right)}\;$$

**Figure 3:**
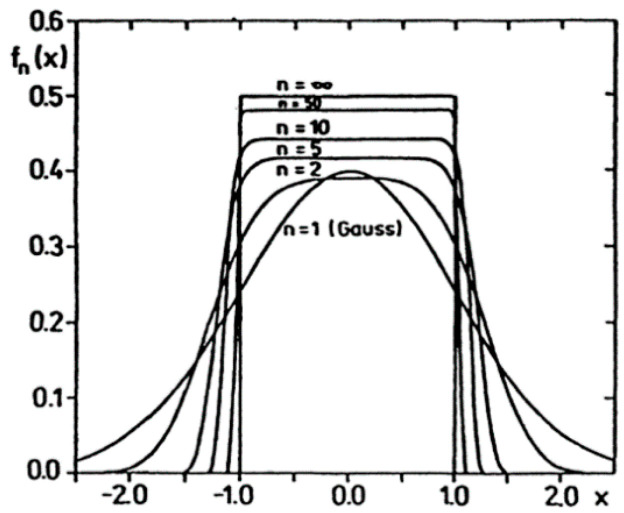
Higher order maximum entropy density for constraints (32) from Ingarden’s paper.

We illustrate this higher order maximum entropy density in Figure 3.

● *2nd Example of Higher Oder Maximum Entropy Density*:

Density of maximum Entropy $S(P)=-\int_{0}^{+\infty }P(x)\log P(x)dx$ under the constraints:

(36) $$P(x)\geq 0,\;\int_{0}^{+\infty }P(x)dx=1\;\mathrm{and}\;E\left(x^{n}\right)=\int_{0}^{+\infty }x^{n}P(x)dx=\sigma^{n}\;$$

is given by:

(37) $$P(x)=\frac{1}{n^{\frac{1}{n}}\sigma .\Gamma \left(1+1/n\right)}\exp\left(-\frac{x^{n}}{n\sigma^{n}}\right)=f_{n}(x)\;$$

with the following parameters:

(38) $$\beta_{n}=\frac{1}{n\sigma^{n}},\;Z\left(\beta_{n}\right)=\frac{\Gamma \left(1+1/n\right)}{\beta_{n}^{1/n}},\;S(P)=\log Z\left(\beta_{n}\right)+\frac{1}{n}\;$$

where:

(39) $$-\frac{\partial \log Z\left(\beta_{k}\right)}{\partial \beta_{k}}=\sigma^{k}=E\left(x^{k}\right)=\frac{n^{k/n}\sigma^{k}\Gamma \left(1+\left(k+1\right)/n\right)}{\left(k+1\right)\Gamma \left(1+1/n\right)}\;$$

**Figure 4:**
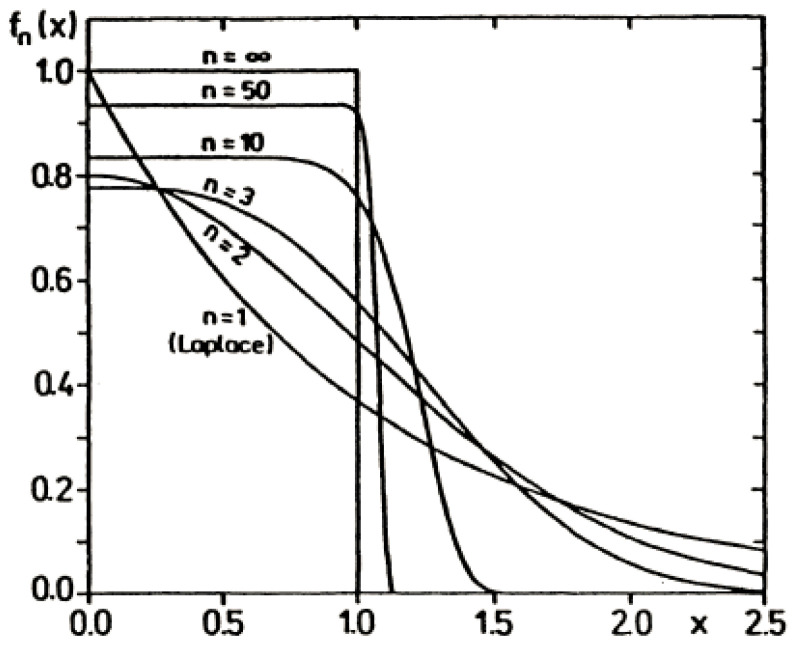
Higher order maximum entropy density for constraints (36) from Ingarden paper.

We illustrate this higher order maximum entropy density in Figure 4.

As soon as 1963, Ingarden has introduced this concept of higher order temperatures for statistical systems such as thermodynamics. In physics, the concept of temperature is connected with the mean value of kinetic energy of molecules in an ideal gas. For a general physical system with interactions among particles (the case of non-ideal gas: liquid or solid), an equilibrium probability distribution depends on temperature *T* as the only statistical parameter of the Gibbs state: $P_{\beta }(x)=\frac{1}{Z\left(\beta \right)}e^{-\beta .H(x)}$ with $\beta =\frac{1}{k_{\beta }T}$ and H(x)=H(p,q) where *p* is position, *q* the mechanical momentum and $k_{\beta }$ the Boltzmann constant (a factor to insure that β.H is dimensionless). If there are no stochastic interactions between particles (ideal gas), the partition function *Z* has the property to be integrable and we can obtain Gauss distribution in the momentum space deduced from the result of the limit theorem for large *N*. The ideal gas model of Boltzmann can fail if the number of particles is not large enough in the case of mesoscopic systems, and also if the interactions between particles are not weak enough. Gibbs hypothesis can also fail in other cases when stochastic interactions with the environment are not sufficiently weak. As remarked by Ingarden, nobody has ever observed thermal Gibbs equilibrium in large and complex systems (cosmic systems, Earth’s atmosphere, biological organisms), but only in cases of turbulence, flows or pumping, by replacing classical approach by local temperature and concept of thermodynamic flows (non-equilibrium thermodynamics and thermo-hydrodynamics), that is non-coherent with the classical concept of temperature which is, by definition, global/intensive and does not depend on position. R.S. Ingarden proposed to consider the stationary case using of the concept of higher order temperatures given by the Gibbs density:

(40) $$P_{\left(\beta_{1},\ldots ,\beta_{n}\right)}(x)=\frac{1}{Z\left(\beta_{1},\ldots ,\beta_{n}\right)}e^{-\beta_{1}.H(x)-\beta_{2}{\left(H(x)-U\right)}^{2}-\ldots -\beta_{n}{\left(H(x)-U\right)}^{n}}\;$$

where U=E(H) is the mean energy. This mean energy has been introduced to preserve the the total energy invariance with respect to an arbitrary additive constant, and $\beta_{0}=-\log Z\left(\beta_{1},\ldots ,\beta_{n}\right)$ the constant of normalization. The new constants $\beta_{k}$ are said to be *β*-temperatures of order *k*. H(x) is usually defined as a quadratic function of *x*. The probability distribution is uniquely defined from statistical moments which should be measured experimentally. But if values number is too high to make this method practical, we are only able to measure the lowest moments up to some order (if we can neglect the higher orders that do not change the result to a given accuracy), and to fix *β*-temperatures defined as Lagrange multipliers by maximization of entropy of distribution $S=-\int P_{\left(\beta_{1},\ldots ,\beta_{n}\right)}(x)\log P_{\left(\beta_{1},\ldots ,\beta_{n}\right)}(x)dx$, with the given moments as constraints. R.S. Ingarden observed that the entropy maximization randomizes higher moments in a symmetric way, and it cancel any possible bias with respect to their special values, and it gives the best estimate to a given accuracy. The values of β can be found by:

(41) $$E\left(x^{k}\right)=\frac{\partial \beta_{0}}{\partial \beta_{k}}=\frac{\partial \log Z}{\partial \beta_{k}}\;\mathrm{with}\;E\left(x^{k}\right)=Z^{-1}\int x^{k}e^{-\sum_{k=1}^{n}\beta_{k}x^{k}}dx=\int x^{k}P_{\left(\beta_{1},\ldots ,\beta_{n}\right)}(x)dx\;$$

(42) $$Z=\int e^{-\sum_{k=1}^{n}\beta_{k}x^{k}}dx\;\mathrm{and}\;\mathrm{the}\;\mathrm{relation}:\;S=\sum_{k=1}^{n}\beta_{k}E\left(x^{k}\right)+\log Z=\sum_{k=1}^{n}\beta_{k}\frac{\partial \beta_{0}}{\partial \beta_{k}}-\beta_{0}\;$$

Ingarden has applied this model for linguistic statistics, assuming the appearance of higher order temperatures since there occur rather strong statistical correlations between phonemes and words as elements of these statistics. He argued his choice observing that in the case of word statistics, the existence of strong correlations is given by grammatical or semantical studies [9]. Ingarden made the conjecture that his high order thermodynamics is the model of statistically interacting, biological living systems, and small systems although the calculation/observation are more difficult.

Ingarden higher order temperatures could be defined in the case when no variation is considered, but when a probability distribution depending on more than one parameter. It has been observed by Ingarden, that Gibbs assumption can fail if the number of components of the sum goes to infinity and the components of the sum are stochastically independent, and if stochastic interactions with the environment are not sufficiently weak. In all these cases, we never observe absolute thermal equilibrium of Gibbs type but only flows or turbulence. Non-equilibrium thermodynamics could be indirectly addressed by means of high order temperatures.

## 4. Model of Souriau Lie Groups Thermodynamics

For introduction to symplectic geometry, we make reference to Marle’s book [45] and Koszul’s book [44]. In 1969, Souriau [43,64] introduced the concept of co-adjoint action of a group on its momentum space, based on the orbit method works, that allows to define physical observables like energy, heat and momentum or moment as pure geometrical objects. The moment map is a constant of the motion and is associated to symplectic cohomology. In a first step to establish new foundations of thermodynamics, Souriau has defined a Gibbs canonical ensemble on a symplectic manifold *M* for a Lie group action on *M*. In classical statistical mechanics, a state is given by the solution of Liouville equation on the phase space, the partition function. As symplectic manifolds have a completely continuous measure, invariant by diffeomorphisms, the Liouville measure *λ*, all statistical states will be the product of the Liouville measure by the scalar function given by the generalized partition function $e^{\Phi (\beta )-\langle \beta ,U(\xi )\rangle }$ defined by the energy U (defined in the dual of the Lie algebra of this dynamical group) and the geometric temperature β, where Φ is a normalizing constant such the mass of probability is equal to 1, $\Phi (\beta )=-\log\int_{M}e^{-\langle \beta ,U(\xi )\rangle }d\lambda$. Souriau then generalizes the Gibbs equilibrium state to all symplectic manifolds that have a dynamical group. Souriau has observed that if we apply this theory for a Galileo group, the symmetry has been broken. For each temperature β, element of the Lie algebra g, Souriau has introduced a tensor ${\tilde{\Theta }}_{\beta }$, equal to the sum of the cocycle $\tilde{\Theta }$ and the heat coboundary (with [.,.] Lie bracket):

(43) $${\tilde{\Theta }}_{\beta }\left(Z_{1},Z_{2}\right)=\tilde{\Theta }\left(Z_{1},Z_{2}\right)+\langle Q,ad_{Z_{1}}(Z_{2})\;\rangle \;$$

This tensor ${\tilde{\Theta }}_{\beta }$ has the following properties: $\tilde{\Theta }(X,Y)=\langle \Theta (X),Y\rangle$ where the map Θ is the symplectic one-cocycle of the Lie algebra g with values in $g^{*}$, with $\Theta (X)=T_{e}\theta \left(X(e)\right)$ where θ the one-cocycle of the Lie group *G*. $\tilde{\Theta }\left(X,Y\right)$ is constant on *M* and the map $\tilde{\Theta }\left(X,Y\right):g\times g\to ℜ$ is a skew-symmetric bilinear form, and is called the *symplectic two-cocycle of Lie algebra* g associated to the *moment map* J, with the following properties:

(44) $$\tilde{\Theta }(X,Y)=J_{\left[X,Y\right]}-\left\{J_{X},J_{Y}\right\}\;\mathrm{with}\;J\;\mathrm{the}\;\mathrm{Moment}\;\mathrm{Map}\;$$

(45) $$\tilde{\Theta }(\left[X,Y\right],Z)+\tilde{\Theta }(\left[Y,Z\right],X)+\tilde{\Theta }(\left[Z,X\right],Y)=0\;$$

where $J_{X}$ linear application from g to differential function on M:$g\to C^{\infty }(M,R),X\to J_{X}$ and the associated differentiable application J, called moment(um) map:

(46) $$J:M\to g^{*}\;,x\mapsto J(x)\;\mathrm{such}\;\mathrm{that}\;J_{X}(x)=\langle J(x),X\rangle ,\;X\in g\;$$

The geometric temperature, element of the algebra g, is in the the kernel of the tensor ${\tilde{\Theta }}_{\beta }$:

(47) $$\beta \in Ker\;{\tilde{\Theta }}_{\beta }\;\mathrm{such}\;\mathrm{that}\;{\tilde{\Theta }}_{\beta }\left(\beta ,\beta \right)=0\;,\;\forall \beta \in g\;$$

The following symmetric tensor $g_{\beta }\left(\left[\beta ,Z_{1}\right],\left[\beta ,Z_{2}\right]\right)={\tilde{\Theta }}_{\beta }\left(Z_{1},\left[\beta ,Z_{2}\right]\right)$, defined on all values of $ad_{\beta }(.)=\left[\beta ,.\right]$ is positive definite, and **d**efines extension of the classical Fisher metric in information geometry (as the Hessian of the logarithm of partition function):

(48) gβ([β,Z1],Z2)=Θ˜β(Z1,Z2) , ∀Z1∈g,∀Z2∈Im(adβ(.)) 

with:

(49) gβ(Z1,Z2)≥0 , ∀Z1,Z2∈Im(adβ(.)) 

**Figure 5:**
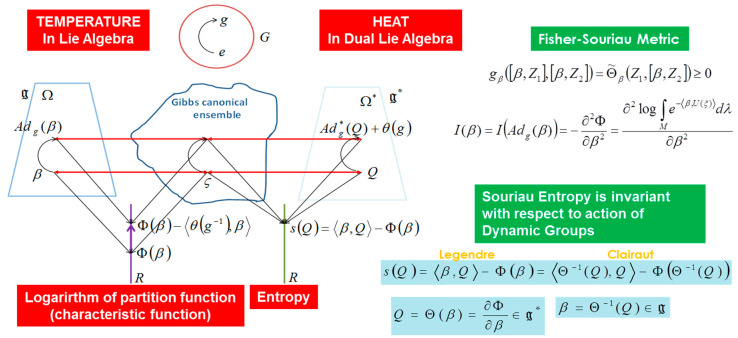
Global Souriau scheme of Lie groups thermodynamics.

**Figure 6:**
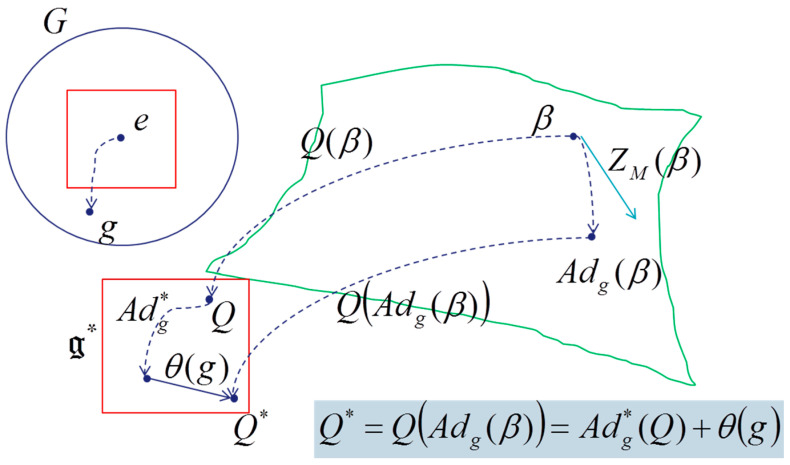
Broken symmetry on geometric heat *Q* due to adjoint action of the group on temperature *β* as an element of the Lie algebra.

These equations are universal, because they are not dependent on the symplectic manifold but only on the dynamical group *G*, the symplectic two-cocycle Θ, the temperature β and the heat Q. Souriau called it “*Lie groups thermodynamics”* (see Figure 5 and Figure 6).

**Theorem** **2.** **[Souriau Theorem of Lie Groups Thermodynamics]** *Let*Ω*be the largest open proper subset of*g*, Lie algebra of G, such that*$\int_{M}e^{-\langle \beta ,U(\xi )\rangle }d\lambda$*and*$\int_{M}\xi .e^{-\langle \beta ,U(\xi )\rangle }d\lambda$*are convergent integrals, this set*Ω*is convex and is invariant under every transformation*$Ad_{g}(.)$. Then, the fundamental equations of Lie groups thermodynamics are given by the action of the group:

- *Action of Lie group on Lie algebra:*
  (50) $$\beta \to Ad_{g}(\beta )\;$$
- *Characteristic function after Lie group action:*
  (51) $$\Phi \to \Phi -\langle \theta \left(g^{-1}\right),\beta \rangle \;$$
- *Invariance of entropy with respect to action of Lie group:*
  (52) s→s 
- *Action of Lie group on geometric heat:*
  (53) $$Q\to a(g,Q)=Ad_{g}^{*}(Q)+\theta \left(g\right)\;$$

Souriau’s equations of Lie groups thermodynamics are summarized in the following figures.

In the framework of Lie group action on a symplectic manifold, equivariance of moment could be studied to prove that there is a unique action *a*(.,.) of the Lie group G on the dual $g^{*}$ of its Lie algebra for which the moment map J is equivariant, that means for each x∈M:

(54) $$J\left(\Phi_{g}(x)\right)=a(g,J(x))=Ad_{g}^{*}\left(J(x)\right)+\theta (g)\;$$

When the group is not abelian (non-commutative group), the symmetry is broken, and new “cohomological” relations should be verified in Lie algebra of the group. A natural equilibrium state will thus be characterized by an element of the Lie algebra of the Lie group, determining the equilibrium temperature β. The entropy s(Q), parametrized by Q the geometric heat (mean of energy U, element of the dual Lie algebra) is defined by the Legendre transform [97,98,99,100,101,102,103] of the Massieu potential Φ(β) parametrized by β (Φ(β) is the minus logarithm of the partition function $\psi_{\Omega }\left(\beta \right)$):

(55) $$s\left(Q\right)=\langle \beta ,Q\rangle -\Phi (\beta )\;\mathrm{with}\;\left\{ \begin{array}{l} Q=\frac{\partial \Phi }{\partial \beta }\in g^{*}\\ \beta =\frac{\partial s}{\partial Q}\in g \end{array}\right.\;$$

(56) $$\begin{array}{l} p_{Gibbs}(\xi )=e^{\Phi (\beta )-\langle \beta ,U(\xi )\rangle }=\frac{e^{-\langle \beta ,U(\xi )\rangle }}{\int_{M}e^{-\langle \beta ,U(\xi )\rangle }d\omega }\;,\;Q=\frac{\partial \Phi (\beta )}{\partial \beta }=\frac{\int_{M}U(\xi )e^{-\langle \beta ,U(\xi )\rangle }d\omega }{\int_{M}e^{-\langle \beta ,U(\xi )\rangle }d\omega }=\int_{M}U(\xi )p(\xi )d\omega \\ \mathrm{with}\;\Phi (\beta )=-\log\int_{M}e^{-\langle \beta ,U(\xi )\rangle }d\omega \end{array}$$

Souriau completed his “geometric heat theory” by introducing a 2-form in the Lie algebra, that is a Riemannian metric tensor in the values of adjoint orbit of β, [β,Z] with Z an element of the Lie algebra. This metric is given for (β,Q):

(57) $$g_{\beta }\left(\left[\beta ,Z_{1}\right],\left[\beta ,Z_{2}\right]\right)=\;\langle \Theta \left(Z_{1}\right),\left[\beta ,Z_{2}\right]\rangle +\langle Q,\left[Z_{1},\left[\beta ,Z_{2}\right]\right]\rangle \;$$

where Θ is a cocycle of the Lie algebra, defined by $\Theta =T_{e}\theta$ with θ a cocycle of the Lie group defined by $\theta (M)=Q\left(Ad_{M}(\beta )\right)-Ad_{M}^{*}Q$.

We observe that Souriau Riemannian metric, introduced with symplectic cocycle, is a generalization of the Fisher metric, that we call the Souriau-Fisher metric, that preserves the property to be defined as a Hessian of the partition function logarithm $g_{\beta }=-\frac{\partial^{2}\Phi }{\partial \beta^{2}}=\frac{\partial^{2}\log\psi_{\Omega }}{\partial \beta^{2}}$ as in classical information geometry. We will establish the equality of two terms, between Souriau definition based on Lie group cocycle Θ and parameterized by “geometric heat” *Q* (element of dual Lie algebra) and “geometric temperature” *β* (element of Lie algebra) and hessian of characteristic function $\Phi \left(\beta \right)=-\log\psi_{\Omega }(\beta )$ with respect to the variable *β*:

(58) $$g_{\beta }\left(\left[\beta ,Z_{1}\right],\left[\beta ,Z_{2}\right]\right)=\;\langle \Theta \left(Z_{1}\right),\left[\beta ,Z_{2}\right]\rangle +\langle Q,\left[Z_{1},\left[\beta ,Z_{2}\right]\right]\rangle =\frac{\partial^{2}\log\psi_{\Omega }}{\partial \beta^{2}}\;$$

If we differentiate this relation of Souriau theorem $Q\left(Ad_{g}(\beta )\right)=Ad_{g}^{*}(Q)+\theta \left(g\right)$, this relation occurs:

(59) $$\frac{\partial Q}{\partial \beta }\left(-\left[Z_{1},\beta \right],.\right)=\tilde{\Theta }\left(Z_{1},\left[\beta ,.\right]\right)+\langle Q,Ad_{.Z_{1}}(\left[\beta ,.\right])\rangle ={\tilde{\Theta }}_{\beta }\left(Z_{1},\left[\beta ,.\right]\right)\;$$

(60) $$-\frac{\partial Q}{\partial \beta }\left(\left[Z_{1},\beta \right],Z_{2}.\right)=\tilde{\Theta }\left(Z_{1},\left[\beta ,Z_{2}\right]\right)+\langle Q,Ad_{.Z_{1}}(\left[\beta ,Z_{2}\right])\rangle ={\tilde{\Theta }}_{\beta }\left(Z_{1},\left[\beta ,Z_{2}\right]\right)\;$$

(61) $$\Rightarrow -\frac{\partial Q}{\partial \beta }=g_{\beta }\left(\left[\beta ,Z_{1}\right],\left[\beta ,Z_{2}\right]\right)\;$$

As the entropy is defined by the Legendre transform of the characteristic function, this Souriau-Fisher metric is also equal to the inverse of the hessian of “geometric entropy” s(Q) with respect to the variable *Q:* $\frac{\partial^{2}s(Q)}{\partial Q^{2}}$.

For the maximum entropy density (Gibbs density), the following three terms coincide: $\frac{\partial^{2}\log\psi_{\Omega }}{\partial \beta^{2}}$ that describes the convexity of the log-likelihood function, $I(\beta )=-E\left[\frac{\partial^{2}\log p_{\beta }(\xi )}{\partial \beta^{2}}\right]$ the Fisher metric that describes the covariance of the log-likelihood gradient, whereas $I(\beta )=E\left[\left(\xi -Q\right){\left(\xi -Q\right)}^{T}\right]=Var(\xi )$ that describes the covariance of the observables.

We can also observe that the Fisher metric $I(\beta )=-\frac{\partial Q}{\partial \beta }$ is exactly the Souriau metric defined through symplectic cocycle:

(62) $$I(\beta )={\tilde{\Theta }}_{\beta }\left(Z_{1},\left[\beta ,Z_{2}\right]\right)=g_{\beta }\left(\left[\beta ,Z_{1}\right],\left[\beta ,Z_{2}\right]\right)\;$$

The Fisher metric $I(\beta )=-\frac{\partial^{2}\Phi (\beta )}{\partial \beta^{2}}=-\frac{\partial Q}{\partial \beta }$ has been considered by Souriau as a *generalization of* “*heat capacity*”. Souriau called it K the “*geometric capacity*”.

We could observe that Souriau Lie groups thermodynamics is compatible with Balian and Valentin’s theory of thermodynamics [24], that is obtained by symplectization in dimension 2*n* + 2 of contact manifold in dimension 2*n* + 1. All elements of the Souriau geometric temperature vector are multiplied by the same gauge parameter. The Balian and Valentin model was first explored in [104] and has been recently developed by der Schaft and Maschke in [26,105].

## 5. Extended Koszul Study of Souriau Non-Equivariant Model Associated to a Class of Cohomology

Koszul has deepened Souriau’s model in his book “*Introduction to symplectic geometry*” [44] as explained in [10]. In the historical foreword of this book, Koszul write “*The development of analytical mechanics provided the basic concepts of symplectic structures. The term symplectic structure is due largely to analytical mechanics. But in this book, the applications of symplectic structure theory to mechanics is not discussed in any detail*”. Koszul considers in this book purely algebraic and geometric developments of geometric/analytic mechanics developed during the 60s, more especially in Jean-Marie Souriau’s works detailed in chapters 4 and 5. The originality of this book lies in the fact that Koszul develops new points of view, and demonstrations not considered initially by Souriau and after by the geometrical mechanics community.

To highlight the importance of this Koszul book, we will illustrate the links of the detailed tools, including demonstrations or original Koszul extensions, with Souriau’s Lie groups thermodynamics. Koszul originally developed Souriau’s model, in the case of non-equivariance, of the action of the group G on the moment map. As explained in [106] by Thomas Delzant at the 2010 CIRM conference “*Action Hamiltoniennes: invariants et classification*”, organized with Michel Brion: “The definition of the moment map is due to Jean-Marie Souriau…. In the book of Souriau, we find a proof of the proposition: the map J is equivariant for an affine action of G on g* whose linear part is Ad*…. In Souriau’s book, we can also find a study of the non-equivariant case and its applications to classical and quantum mechanics. In the case of the Galileo group operating in the phase space of space-time, obstruction to equivariance (a class of cohomology) is interpreted as the inert mass of the object under study”. We can uniquely define the moment map up to an additive constant of integration, that can always be chosen to make the moment map equivariant (a moment map is *G*-equivariant, when *G* acts on g* via the coadjoint action) if the group is compact or semi-simple. In 1969, Souriau has considered the non-equivariant case where the coadjoint action must be modified to make the map equivariant by a 1-cocycle on the group with values in dual Lie algebra g*.

The concept and seminal idea of moment map was in the Sophus *Lie’s book* 2nd volume published in 1890, developed for homogeneous canonical transformations. Professor Marsden has summarized the development of this concept by Jean-Marie Souriau and Bertram Kostant based on their both testimonials: “*In Kostant’s 1965 Phillips lectures at Haverford, and in the 1965 U.S.–Japan Seminar, Kostant introduced the momentum map to generalize a theorem of Wang and thereby classified all homogeneous symplectic manifolds; this is called today ‘Kostant’s coadjoint orbit covering theorem’…. Souriau introduced the momentum map in his 1965 Marseille lecture notes and put it in print in 1966. The momentum map finally got its formal definition and its name, based on its physical interpretation, by Souriau in 1967. Souriau also studied its properties of equivariance, and formulated the coadjoint orbit theorem. The momentum map appeared as a key tool in Kostant’s quantization lectures in 1970 [46], and Souriau discussed in 1970 it at length in his book [43]. Kostant and Souriau realized its importance for linear representations, a fact apparently not foreseen by Lie*”. Souriau’s book reference date is 1970, but it was published by Dunod in 1969. For information, Jean-Louis Koszul knew very well the Souriau and Kostant works, and as soon as 1958, Koszul made a survey of first Kostant’s works at a Bourbaky seminar [47]. 

In this book in Chapter 4, Koszul calls symplectic *G*-space a symplectic manifold (*M*; *ω*) on which a Lie group *G* acts by a symplectic action (an action which leaves unchanged the symplectic form *ω*). Koszul then introduces and develop properties of the moment map *μ* (Souriau’s invention) of a Hamiltonian action of the Lie algebra *g*. Koszul also defines the Souriau 2-cocycle, considering that the difference of two moments of the same Hamiltonian action is a locally constant application on *M* ,showing that when *μ* is a moment map, for every pair (*a*;*b*) of elements of *g*, the function $c_{\mu }(a,b)=\left\{\langle \mu ,a\rangle ,\langle \mu ,b\rangle \right\}-\langle \mu ,\left\{a,b\right\}\rangle$ is locally constant on *M*, defining an antisymmetric bilinear application of *g*x*g* in *H^0^(M; R)* which verifies Jacobi’s identity. This is the 2-cocycle introduced by Jean-Marie Souriau in Geometric Mechanics, that will play a fundamental role in Souriau Lie Groups Thermodynamics to define an extension of the Fisher Metric from Information Geometry: “Fisher-Souriau metric”.

*The antisymmetric bilinear map (31) and (32), with definition (27) and (28), introduced by Souriau is exactly equal to the mathematical object extensively studied in Chapter 4 of Koszul’s book:*

(63) $$c_{\mu }(a,b)=\left\{\langle \mu ,a\rangle ,\langle \mu ,b\rangle \right\}-\langle \mu ,\left\{a,b\right\}\rangle \;$$

In this book, Koszul has studied this antisymmetric bilinear map considering the following developments. For any moment map μ, Koszul defines the skew symmetric bilinear form $c_{\mu }\left(a,b\right)$ on Lie algebra by:

(64) $$c_{\mu }(a,b)=\langle d\theta_{\mu }(a),b\rangle \;,\;a,b\in g\;$$

Koszul observes that if he uses:

(65) $$\theta_{\mu }(st)=\mu (stx)-Ad_{st}^{*}\mu (x)=\theta_{\mu }(s)+Ad_{s}^{*}\mu (tx)-Ad_{s}^{*}Ad_{t}^{*}\mu (x)=\theta_{\mu }(s)+Ad_{s}^{*}\theta_{\mu }(t)\;$$

by developing dμ(ax)=adatμ(x)+dθμ(a) , x∈M,a∈g, he obtains:

(66) $$\langle d\mu (ax),b\rangle =\langle \mu (x),\left[a,b\right]\rangle +\langle d\theta_{\mu }(a),b\rangle \;=\left\{\langle \mu ,a\rangle ,\langle \mu ,b\rangle \right\}(x)\;,\;x\in M,a,b\in g\;$$

He has then:

(67) $$c_{\mu }(a,b)=\left\{\langle \mu ,a\rangle ,\langle \mu ,b\rangle \right\}-\langle \mu ,\left[a,b\right]\rangle =\langle d\theta_{\mu }(a),b\rangle \;,\;a,b\in g\;$$

and the property:

(68) $$c_{\mu }\left(\left[a,b\right],c\right)+c_{\mu }\left(\left[b,c\right],a\right)+c_{\mu }\left(\left[c,a\right],b\right)=0\;,\;a,b,c\in g\;$$

Koszul concludes by observing that if the moment map is transform as μ′=μ+ϕ then we have:

(69) $$c_{\mu \prime }(a,b)=c_{\mu }(a,b)-\langle \phi ,\left[a,b\right]\rangle \;$$

Finally using $c_{\mu }(a,b)=\left\{\langle \mu ,a\rangle ,\langle \mu ,b\rangle \right\}-\langle \mu ,\left[a,b\right]\rangle =\langle d\theta_{\mu }(a),b\rangle \;,\;a,b\in g$, koszul highlights the property that:

(70) $$\left\{\mu^{*}(a),\mu^{*}(b)\right\}=\left\{\langle \mu ,a\rangle ,\langle \mu ,b\rangle \right\}=\mu^{*}\left(\left[a,b\right]+c_{\mu }(a,b)\right)=\mu^{*}{\left\{a,b\right\}}_{c_{\mu }}\;$$

In Chapter 4, Koszul introduces the equivariance of the moment map *μ*. Based on the definitions of the adjoint and coadjoint representations of a Lie group or a Lie algebra, Koszul proves that when (*M*; *ω*) is a connected Hamiltonian *G*-space and $\mu :M\to g^{*}$ a moment of the action of *G*, there exists an affine action of *G* on g*, whose linear part is the coadjoint action, for which the moment *μ* is equivariant. This affine action is obtained by modifying the coadjoint action by means of a cocycle. This notion is also developed in Chapter *5* for studying Poisson manifolds.

Defining classical operation $Ad_{s}a=sas^{-1}\;,\;s\in G,a\in g$, $ad_{a}b=\left[a,b\right]\;,\;a\in g,b\in g$ and Ads*=Ads-1t, s∈G with classical properties:

(71) Adexpa=exp(−ada) , a∈g or Adexpa*=exp(ada)t , a∈g 

Koszul considers:

(72) $$x\mapsto sx\;,\;x\in M,\;\mu :M\to g^{*}\;$$

From which, he obtains:

(73) 〈dμ(v),a〉=ω(ax,v) 

Koszul then study $\mu \circ s_{M}-Ad_{s}^{*}\circ \mu :M\to g^{*}$, and develops:

(74) $$d\langle Ad_{s}^{*}\circ \mu ,a\rangle =\langle Ad_{s}^{*}d\mu ,a\rangle =\langle d\mu ,Ad_{s^{-1}}a\rangle \;$$

(75) $$\langle d\mu (v),Ad_{s^{-1}}a\rangle =\omega \left(s^{-1}asx,v\right)=\omega \left(asx,sv\right)=\langle d\mu (sv),a\rangle =\left(d\langle \mu \circ s_{M},a\rangle \right)(v)\;$$

(76) $$d\langle Ad_{s}^{*}\circ \mu ,a\rangle =d\langle \mu \circ s_{M},a\rangle \;\mathrm{and}\;\mathrm{then}\;\mathrm{proves}\;\mathrm{that}\;d\langle \mu \circ s_{M}-Ad_{s}^{*}\circ \mu ,a\rangle =0\;$$

Koszul considers the cocycle given by $\theta_{\mu }(s)=\mu (sx)-Ad_{s}^{*}\mu (x)\;,\;s\in G$, and observes that:

(77) $$\theta_{\mu }(st)=\theta_{\mu }(s)-Ad_{s}^{*}\theta_{\mu }(t)\;,\;s,t\in G\;$$

From this action of the group on dual Lie algebra:

(78) $$G\times g^{*}\to g^{*},(s,\xi )\mapsto s\xi =Ad_{s}^{*}\xi +\theta_{\mu }(s)\;$$

Koszul introduces the following properties:

(79) $$\mu (sx)=s\mu (x)=Ad_{s}^{*}\mu (x)+\theta_{\mu }(s)\;,\;\forall s\in G,x\in M\;$$

(80) $$G\times g^{*}\to g^{*},\left(e,\xi \right)\mapsto e\xi =Ad_{e}^{*}\xi +\theta_{\mu }(e)=\xi +\mu (x)-\mu (x)=\xi \;$$

(81) $$\begin{array}{l} \left(s_{1}s_{2})\xi =Ad_{s_{1}s_{2}}^{*}\xi +\theta_{\mu }(s_{1}s_{2})=Ad_{s_{1}}^{*}Ad_{s_{2}}^{*}\xi +\theta_{\mu }(s_{1})+Ad_{s_{1}}^{*}\theta_{\mu }(s_{2}\right)\\ (s_{1}s_{2})\xi =Ad_{s_{1}}^{*}\left(Ad_{s_{2}}^{*}\xi +\theta_{\mu }(s_{2})\right)+\theta_{\mu }(s_{1})=s_{1}\left(s_{2}\xi \right)\;,\;\forall s_{1},s_{2}\in G,\xi \in g^{*} \end{array}$$

This Koszul study of the moment map *μ* equivariance, and the existence of an affine action of G on g*, whose linear part is the coadjoint action, for which the moment *μ* is equivariant, is at the cornerstone of Souriau theory of geometric mechanics and Lie groups thermodynamics.

**Figure 7:**
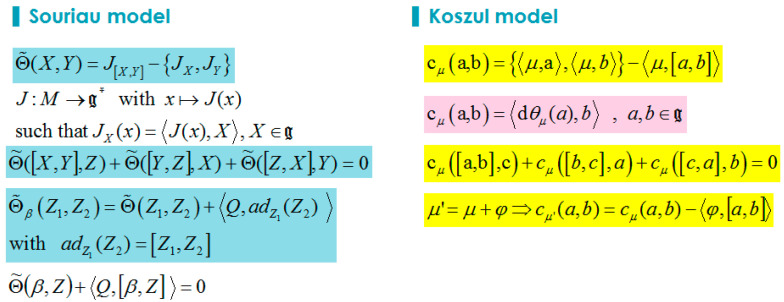
Comparison of the Souriau equations (column on the left) and Koszul equations (column on the right).

We compare Souriau and Koszul notations in Figure 7.

We have also to make reference to Muriel Casalis’ papers [41,42] on this topic.

## 6. Souriau Model of Generalized Entropy Based on Legendre and Laplace Transforms

At the step of the development of Souriau Lie groups thermodynamics, we will introduce generalized Souriau definition of entropy. Souriau first start to define “Laplace transform”:

Let E a vector space of finite size, μ a measure of its dual $E^{*}$, then the function given by:

(82) $$\alpha \mapsto \int_{E^{*}}e^{M\alpha }\mu (M)dM\;$$

for all α∈E such that the integral is convergent. This function is called (generalized) Laplace transform. This transform F of the measure μ is differentiable inside is definition set def(F). Its *p*-th derivative is given by the following convergent integral for all point inside def(F):

(83) $$F^{\left(p\right)}(\alpha )=\int_{E^{*}}M⊗M\ldots ⊗M\mu (M)dM\;$$

**Theorem** **3.** **[Souriau Theorem]** *Let* E *a vector space of finite size,* μ *a non-zero positive measure of dual space* $E^{*}$ *,* F *its Laplace transform, then:*

- F *is semi-definite convex function,*
  (84) F(α)>0,∀α∈def(F) 
- f=logF *is convex and semi-continuous*
- *Let* α *an interior point of* def(F) *then:*
  (85) $$D^{2}(f)(\alpha )\geq 0\;$$
  (86) $$D^{2}(f)(\alpha )=\int_{E^{*}}e^{M\alpha }{\left[M-D(f)(\alpha )\right]}^{{⊗}^{2}}\mu (M)dM\;$$
  (87) $$D^{2}(f)(\alpha )\text{ inversible }\Leftrightarrow \text{Affine envelop}(\mu ))=E^{*}\;$$

*See [107], for links between dual convex functions and optimization.*

Before introducing Entropy, Souriau introduced the following lemma:

**Lemma** **1.** *Let* X *be a locally compact space, Let* λ *a positive measure of* X *, having* X *as support, then the following function* Φ *is convex:*

(88) $$\Phi (h)=\log\int_{X}e^{h(X)}\lambda (x)dx\;,\;\forall h\in C(X)\;$$

*such that the integral is converging.*

The integral is strictly positive when it converges, and then insures existence of its logarithm. The epigraph of Φ is the set of $\left(\begin{array}{c} h\\ y \end{array}\right)$ such that $\int_{X}e^{h(x)-y}\lambda (x)dx\leq 1$. Convexity of exponential shows that this epigraph is convex. Finally, Souriau introduced the “negentropy” as Legendre transform of the function Φ:

**Definition1.** **[Souriau Entropy Definition]** *We call “Boltzmann Law” (relative to*λ*) all measure*μ*of*X*such that the set of real values:*

(89) μ(h)−Φ(h),h∈def(Φ) and h is μ-integrable

This definition of entropy by Souriau is a general scheme that can be extended to highly abstract spaces preserving Legendre structure [108], if we can define generalized Laplace transform. These operations of Laplace and Legendre transforms are the core contextures of theory of Information and Heat, generating the well-defined structures, from which we can preserve the definition of “average value”. Jean-Marie Souriau explained this contexture property in the following sentence:

“*Il est évident que l’on ne peut définir de valeurs moyennes que sur des objets appartenant à un espace vectoriel (ou affine); donc—si bourbakiste que puisse sembler cette affirmation—que l’on n’observera et ne mesurera de valeurs moyennes que sur des grandeurs appartenant à un ensemble possédant physiquement une structure affine. Il est clair que cette structure est nécessairement unique—sinon les valeurs moyennes ne seraient pas bien définies.*” (In English: *It is obvious that one can only define average values on objects belonging to a vector (or affine) space; Therefore—so this assertion may seem Bourbakist—that we will observe and measure average values only as quantity belonging to a set having physically an affine structure. It is clear that this structure is necessarily unique—if not the average values would not be well defined*.)

See also papers of Kostant [109] and Leray [100] for generalized Laplace transforms.

## 7. Illustration of Souriau Thermodynamics of a Centrifuge System

Duhem [110,111,112,113] and Poincaré [114] have studied statistical mechanics model of centrifuges. We will illustrate Souriau’s Lie groups thermodynamics for Souriau Gibbs states for Hamiltonian actions of subgroups of the Galilean group, as illustrated in Souriau’s book [43] and more recentltly by Charles-Michel Marle [23].

Consider a Galilean Lie group:

(90) $$\left(\begin{array}{ccc} A & \vec{b} & \vec{d}\\ 0 & 1 & e\\ 0 & 0 & 1 \end{array}\right)\;\mathrm{with}\;\left\{ \begin{array}{l} A\in SO(3):\;\mathrm{rotation}\\ \vec{b}\in R^{3}:\;\mathrm{boost}\\ \vec{d}\in R^{3}:\mathrm{space}\;\mathrm{translation}\\ e:\;\mathrm{time}\;\mathrm{translation}\; \end{array}\right.\;$$

Galilean Lie algebra:

(91) $$\left(\begin{array}{ccc} j\left(\vec{\omega }\right) & \vec{\alpha } & \vec{\delta }\\ 0 & 1 & \varepsilon \\ 0 & 0 & 0 \end{array}\right)\;\mathrm{with}\;\left\{ \begin{array}{l} \vec{\omega }=\left(\begin{array}{c} \omega_{x}\\ \omega_{y}\\ \omega_{z} \end{array}\right),\vec{\alpha }\;\mathrm{and}\;\vec{\delta }\in R^{3},\;\varepsilon \in R\\ j\left(\vec{\omega }\right)=\left(\begin{array}{ccc} 0 & -\omega_{z} & \omega_{y}\\ \omega_{z} & 0 & -\omega_{x}\\ -\omega_{y} & \omega_{x} & 0 \end{array}\right)\in \mathrm{so}(3),j\left(\vec{\omega }\right)\vec{r}=\vec{\omega }\times \vec{r} \end{array}\right.\;$$

Action of Lie group:

(92) $$\left(\begin{array}{ccc} A & \vec{b} & \vec{d}\\ 0 & 1 & e\\ 0 & 0 & 1 \end{array}\right)\left(\begin{array}{c} \vec{r}\\ t\\ 1 \end{array}\right)=\left(\begin{array}{c} A\vec{r}+t\vec{b}+\vec{d}\\ t+e\\ 1 \end{array}\right)\;\mathrm{with}\;\vec{r}=\left(\begin{array}{c} x\\ y\\ z \end{array}\right)\;$$

Galilean transformation on position and speed is given by:

(93) $$\left(\begin{array}{cc} \vec{r}\prime & \vec{v}\prime \\ t\prime & 1\\ 1 & 0 \end{array}\right)=\left(\begin{array}{ccc} A & \vec{b} & \vec{d}\\ 0 & 1 & e\\ 0 & 0 & 1 \end{array}\right)\left(\begin{array}{cc} \vec{r} & \vec{v}\\ t & 1\\ 1 & 0 \end{array}\right)=\left(\begin{array}{cc} A\vec{r}+t\vec{b}+\vec{d} & A\vec{v}+\vec{b}\\ t+e & 1\\ 1 & 0 \end{array}\right)\;$$

Souriau has proved that this action is Hamiltonian, with the map *J*, defined on the evolution space of the particle, with value in the dual g* of the Lie algebra *G*, as momentum map:

(94) $$J\left(\vec{r},t,\vec{v},m\right)=m\left(\begin{array}{ccc} \vec{r}\times \vec{v} & 0 & 0\\ \vec{r}-t\vec{v} & 0 & 0\\ \vec{v} & \frac{1}{2}{\left\|\vec{v}\right\|}^{2} & 0 \end{array}\right)=m\left\{\vec{r}\times \vec{v},\vec{r}-t\vec{v},\vec{v},\frac{1}{2}{\left\|\vec{v}\right\|}^{2}\right\}\in g^{*}\;$$

where the coupling formula is given by:

(95) $$\begin{array}{l} \langle J\left(\vec{r},t,\vec{v},m\right),\beta \rangle =\langle m\left\{\vec{r}\times \vec{v},\vec{r}-t\vec{v},\vec{v},\frac{1}{2}{\left\|\vec{v}\right\|}^{2}\right\},\left\{\vec{\omega },\vec{\alpha },\vec{\delta },\varepsilon \right\}\rangle \\ \langle J\left(\vec{r},t,\vec{v},m\right),\beta \rangle =m\left(\vec{\omega }.\vec{r}\times \vec{v}-(\vec{r}\times \vec{v}).\vec{\alpha }+\vec{v}.\vec{\delta }-\frac{1}{2}{\left\|\vec{v}\right\|}^{2}\varepsilon \right) \end{array}$$

with:

(96) $$Z=\left(\begin{array}{ccc} j\left(\vec{\omega }\right) & \vec{\alpha } & \vec{\delta }\\ 0 & 1 & \varepsilon \\ 0 & 0 & 0 \end{array}\right)=\left\{\vec{\omega },\vec{\alpha },\vec{\delta },\varepsilon \right\}\in g\;$$

Souriau gave the demonstration for the Galilean moment map for a free particle, considering the definition of moment map:

(97) σ(dp)(δp)=−d〈J,Z〉 , ∀dp 

and the definition of tangent vector field:

(98) $$Z_{V}(p)=\delta \left[a_{V}(p)\right]\;$$

(99) $$Z=\left(\begin{array}{ccc} j\left(\vec{\omega }\right) & \vec{\alpha } & \vec{\delta }\\ 0 & 1 & \varepsilon \\ 0 & 0 & 0 \end{array}\right)\in g\underset{Z_{V}(p)=\delta \left[a_{V}(p)\right]}{\Rightarrow }\left\{ \begin{array}{l} \delta t=\varepsilon \\ \delta r_{j}=\vec{\omega }\times r_{j}+\vec{\alpha }t+\vec{\delta }\\ \delta v_{j}=\vec{\omega }\times v_{j}+\vec{\alpha } \end{array}\right.\;$$

Then, as General Lagrange 2 form for a force *F* is:

(100) $$dp=\left(\begin{array}{c} dt\\ dr\\ dv \end{array}\right)\;\mathrm{and}\;\delta p=\left(\begin{array}{c} \delta t\\ \delta r\\ \delta v \end{array}\right)\Rightarrow \sigma \left(dp\right)\left(\delta p\right)=\langle mdv-Fdt,\delta r-v\delta t\rangle -\langle m\delta v-F\delta t,dr-vdt\rangle \;$$

If *F* is equal to zero, we obtain:

(101) $$\begin{array}{l} \sigma \left(dp\right)\left(\delta p\right)=\sum_{j}\langle mdv,\vec{\omega }\times r_{j}+\vec{\alpha }t+\vec{\delta }-v\varepsilon \rangle -\langle m\left(\vec{\omega }\times v_{j}+\vec{\alpha }\right),dr-vdt\rangle \\ \sigma \left(dp\right)\left(\delta p\right)==-d\langle J,Z\rangle =-dJ_{Z}=-dH \end{array}$$

and the co-cycle is given by:

(102) $$\theta (g)=J\left(Ad_{g}Z\right)-Ad_{g}^{*}\left(J(Z)\right)=\left\{\vec{d}\times \vec{b},\vec{d}-\vec{b}e,\vec{b},\frac{1}{2}{\left\|\vec{b}\right\|}^{2}\right\}\;$$

The main Souriau idea was to define the Gibbs states for one-parameter subgroups of the Galilean group. Souriau has proved that action of the full Galilean group on the space of motions of an isolated mechanical system is not related to any equilibrium Gibbs state (the open subset of the Lie algebra, associated to this Gibbs state, is empty). Then, if we consider the 1-parameter subgroup of the Galilean group generated by b element of Lie algebra, is the set of matrices:

(103) $$\exp(\tau \beta )=\left(\begin{array}{ccc} A(\tau ) & \vec{b}(\tau ) & \vec{d}(\tau )\\ 0 & 1 & \tau \varepsilon \\ 0 & 0 & 1 \end{array}\right)\;\mathrm{with}\;\left\{ \begin{array}{l} A(\tau )=\exp\left(\tau j(\vec{\omega })\right)\;\mathrm{and}\;\vec{b}(\tau )=\left(\sum_{i=1}^{\infty }\frac{\tau^{i}}{i!}{\left(j(\vec{\omega })\right)}^{i-1}\right)\vec{\alpha }\\ \vec{d}(\tau )=\left(\sum_{i=1}^{\infty }\frac{\tau^{i}}{i!}{\left(j(\vec{\omega })\right)}^{i-1}\right)\vec{\delta }+\varepsilon \left(\sum_{i=2}^{\infty }\frac{\tau^{i}}{i!}{\left(j(\vec{\omega })\right)}^{i-2}\right)\vec{\alpha } \end{array}\right.\;$$

and:

(104) $$\beta =\left(\begin{array}{ccc} j\left(\vec{\omega }\right) & \vec{\alpha } & \vec{\delta }\\ 0 & 1 & \varepsilon \\ 0 & 0 & 0 \end{array}\right)\in g\;$$

Then, Gibbs state defined for a gas enclosed in a moving box could be computed by Souriau formula. If we fix the affine Euclidean reference frame $\left(0,{\vec{e}}_{x},{\vec{e}}_{y},{\vec{e}}_{z}\right)$ at t=0, if we set the value τ=t/ε, moving frame $\left(0,{\vec{e}}_{x}(t),{\vec{e}}_{y}(t),{\vec{e}}_{z}(t)\right)$ velocity and acceleration are given by the vector field related to β element of the Lie algebra. For each point, we can associate a rotation speed $\left\|\vec{\omega }\right\|/\varepsilon$, a speed $\vec{\delta }/\varepsilon$ and an acceleration $\vec{\alpha }/\varepsilon$. If we consider a gas made of *N* point particles, indexed by *i ∈ {1,2, …, N}*, enclosed in a box with rigid and undeformable walls, whose motion is described by the action of the 1-parameter subgroup of the Galilean group, A(t/ε) where *t* ∈ *R*. If we consider $m_{i},r_{i}(t),v_{i}(t)$, respectively the mass, position vector and velocity vector of the *i*th particle at time *t*. If we assume free particle and we neglect contributions given by the collisions of the particles between themselves collisions with the walls, then we can write:

(105) $$\langle J,\beta \rangle =\sum_{i=1}^{N}\langle J_{i},\beta \rangle \;\mathrm{with}\;\langle J_{i}\left({\vec{r}}_{i},t,{\vec{v}}_{i},m_{i}\right),\beta \rangle =m_{i}\left(\vec{\omega }.\left({\vec{r}}_{i}\times {\vec{v}}_{i}\right)-({\vec{r}}_{i}-t{\vec{v}}_{i}).\vec{\alpha }+{\vec{v}}_{i}.\vec{\delta }-\frac{1}{2}{\left\|{\vec{v}}_{i}\right\|}^{2}\varepsilon \right)\;$$

The important idea is to observe that $\langle J_{i},\beta \rangle$ is invariant by the action of 1-parameter subgroup. The proof of $\langle J_{i},\beta \rangle$ invariance is based on Souriau equation for default of equivariance with cocyle. If the action of the 1-parameter subgroup is $\exp\left(\frac{t}{\varepsilon }\beta \right)$, according to Souriau equation:

(106) $$a(g,J)=Ad_{g}^{*}\left(J\right)+\theta (g)\;$$

We obtain for:

$$\langle J_{i}(p),\beta \rangle =\langle Ad_{g}^{*}\left(J_{i}(p_{0}\right),\beta \rangle +\langle \theta (g),\beta \rangle =\langle J_{i}(p_{0}),Ad_{g^{-1}}\beta \rangle +\langle \theta (g),\beta \rangle \;$$

that can be reduded by using the properties:

(107) $$\left\{ \begin{array}{l} Ad_{g^{-1}}\beta =\beta \\ \langle \theta (g),\beta \rangle =0 \end{array}\right.\Rightarrow \langle J_{i}(p),\beta \rangle =\langle J_{i}(p_{0}),\beta \rangle \;$$

and:

(108) $$\begin{array}{ll} \mathrm{at}\;t=0\;\mathrm{then}\;\langle J_{i}\left({\vec{r}}_{i},t,{\vec{v}}_{i},m_{i}\right),\beta \rangle & =m_{i}\left(\vec{\omega }.\left({\vec{r}}_{i0}\times {\vec{v}}_{i0}\right)-{\vec{r}}_{i0}.\vec{\alpha }+{\vec{v}}_{i0}.\vec{\delta }-\frac{1}{2}{\left\|{\vec{v}}_{i}\right\|}^{2}\varepsilon \right)\\ & =m_{i}\left({\vec{v}}_{i0}.\left(\vec{\omega }\times {\vec{v}}_{i0}+\vec{\delta }\right)-{\vec{r}}_{i0}.\vec{\alpha }-\frac{1}{2}{\left\|{\vec{v}}_{i}\right\|}^{2}\varepsilon \right) \end{array}$$

To obtain Souriau’s Gibbs maximum entropy density, we have to use the following change of variables:

(109) $${\vec{U}}^{*}=\frac{1}{\varepsilon }\left(\vec{\omega }\times {\vec{v}}_{i0}+\vec{\delta }\right)\;$$

(110) $$\langle J_{i}\left({\vec{r}}_{i},t,{\vec{v}}_{i},m_{i}\right),\beta \rangle =m_{i}\varepsilon \left(-\frac{1}{2}{\left\|{\vec{v}}_{i0}-{\vec{U}}^{*}\right\|}^{2}-{\vec{r}}_{i0}.\frac{\vec{\alpha }}{\varepsilon }+\frac{1}{2}{\left\|{\vec{U}}^{*}\right\|}^{2}\right)\;$$

We can then write:

(111) $$\begin{array}{l} \langle J_{i}\left({\vec{r}}_{i0},{\vec{p}}_{i0}\right),\beta \rangle =-\varepsilon \left(-\frac{1}{2m_{i}}{\left\|{\vec{p}}_{i0}\right\|}^{2}+m_{i}f_{i}\left({\vec{r}}_{i0}\right)\right)\;\mathrm{with}\;\varepsilon =-\frac{1}{\kappa T}\\ \mathrm{with}\;\left\{ \begin{array}{l} {\vec{p}}_{i0}=m_{i}{\vec{w}}_{i0}=m_{i}\left({\vec{v}}_{i0}-{\vec{U}}^{*}\right)\\ f_{i}\left({\vec{r}}_{i0}\right)={\vec{r}}_{i0}.\frac{\vec{\alpha }}{\varepsilon }-\frac{1}{2\varepsilon^{2}}{\left\|\vec{\omega }\times {\vec{r}}_{i0}\right\|}^{2}-\frac{\vec{\delta }}{\varepsilon }.\left(\frac{\vec{\omega }}{\varepsilon }\times {\vec{r}}_{i0}\right)-\frac{1}{2\varepsilon^{2}}{\left\|\vec{\delta }\right\|}^{2} \end{array}\right. \end{array}$$

and finally, the Souriau Gibbs density is given by:

(112) $$\rho \left(\beta \right)=\prod_{i=1}^{N}\rho_{i}\left(\beta \right)\;\mathrm{with}\;\rho_{i}\left(\beta \right)=\frac{1}{P_{i}\left(\beta \right)}\exp\left(-\langle J_{i},\beta \rangle \right)\;$$

(113) $$P_{i}\left(\beta \right)=\int_{M_{i}}\exp\left(-\langle J_{i},\beta \rangle \right)d\lambda_{\omega_{i}}\;,\;Q_{i}\left(\beta \right)=\int_{M_{i}}J_{i}\exp\left(-\langle J_{i},\beta \rangle \right)d\lambda_{\omega_{i}}\;\mathrm{et}\;P\left(\beta \right)=\prod_{i=1}^{N}P_{i}\left(\beta \right)\;$$

If we consider the case of the centrifuge (as for a butter churn, device used to convert cream into butter), the parameter of Galilean group Lie algebra are reduced to:

(114) $$\begin{array}{l} \vec{\omega }=\omega {\vec{e}}_{z}\;,\;\vec{\alpha }=0\;\mathrm{and}\;\vec{\delta }=0\\ Rotation\;speed:\frac{\omega }{\varepsilon } \end{array}\;\mathrm{with}\;\beta =\left(\begin{array}{ccc} j\left(\vec{\omega }\right) & \vec{\alpha } & \vec{\delta }\\ 0 & 1 & \varepsilon \\ 0 & 0 & 0 \end{array}\right)\in g\;$$

with variables:

(115) $$f_{i}\left({\vec{r}}_{i0}\right)=-\frac{\omega^{2}}{2\varepsilon^{2}}{\left\|{\vec{e}}_{z}\times {\vec{r}}_{i0}\right\|}^{2}\;\mathrm{with}\;\Delta =\left\|{\vec{e}}_{z}\times {\vec{r}}_{i0}\right\|\;\mathrm{distance}\;\mathrm{to}\;\mathrm{axis}\;z\;$$

We obtain the closed form for maximum entropy Souriau-Gibbs density:

(116) $$\rho_{i}\left(\beta \right)=\frac{1}{P_{i}\left(\beta \right)}\exp\left(-\langle J_{i},\beta \rangle \right)=cst.\exp\left(-\frac{1}{2m_{i}\kappa T}{\left\|{\vec{p}}_{i0}\right\|}^{2}+\frac{m_{i}}{2\kappa T}{\left(\frac{\omega }{\varepsilon }\right)}^{2}\Delta^{2}\right)\;$$

**Figure 8:**
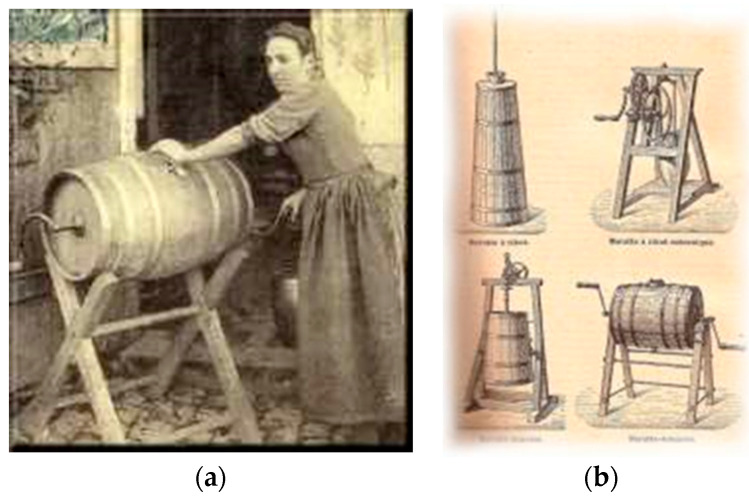
Most simple use-case of Souriau’s Lie groups thermodynamics: the thermodynamics of the centrifuge of butter churn (device used to convert cream into butter). (**a**) butter churn centrifuge with horizontal axis; (**b**) butter churn centrifuge with vertical axis.

This equation describes the behaviour of a gas made of point particles of various masses in a centrifuge rotating at a constant angular velocity and explains the observation that the heavier particles concentrate farther from the rotation axis than the lighter ones. Souriau made reference to thermodynamics of butter churn (see Figure 8).

Souriau Lie groups thermodynamics provides right results if we apply it to subgroups of Galileo group, as previous example of a cylindrical box with fluid with an invariance sub-group of size 2 (rotation along the axis, time translation) providing a 2-dimensional Souriau (Planck) temperature-vector. Souriau has observed that the process, by which a refrigerated centrifuge transmits its own temperature-vector to its content, has two names: thermal conduction and viscosity, depending on the temperature-vector component that is considered. Conduction and viscosity should therefore be unified in a fundamental theory of irreversible processes (theory that remains to be constructed).

In the Appendix, we develop a solution given by Roger Balian [25] for the previous case of centrifuge thermodynamics based on classical methods. Balian recover the same Gibbs density but by introducing an additional Lagrange hyper-parameter associated to total angular momentum. Balian has computed the Boltzmann-Gibbs distribution without knowing the Souriau equations (exercice 7b of). Balian started by considering the constants of motion that are the energy and the component $J_{z}$ of the total angular momentum $J=\sum_{i}\left(r_{i}\times p_{i}\right)$. Balian observed that he must add to the Lagrangian parameter, given by (Planck) temperature β for energy, an additional one associated with $J_{z}$. He identifies this additional multiplier with −βω by evaluating the mean velocity at each point. He then introduced the same results also by changing the frame of reference, the Lagrangian and the Hamiltonian in the rotating frame and by writing down the canonical equilibrium in that frame. He uses the resulting distribution to find, through integration, over the momenta, an expression for the particles density as the function of the distance from the cylinder axis. The main Souriau model advantage is that we can define covariant Gibbs density for dynamical systems, only by applying formulas without any considerations [64].

## 8. Higher-Order Model of Lie Groups Thermodynamics Based on Poly-Symplectic Vector Valued Model

As observed by Souriau in Chapter IV of [43], the Gausian density is a maximum entropy density of 1st order. Considering multivariate Gaussian density, this remark is clear if we replace classical parameterization z and (m,R) by the new parameterization, linked to information geometry coordinates, ξ and β:

(117) $$\begin{array}{l} p_{\left(m,R\right)}(z)=\frac{1}{{\left(2\pi \right)}^{n/2}\det{\left(R\right)}^{1/2}}e^{-\frac{1}{2}{\left(z-m\right)}^{T}R^{-1}(z-m)}=\frac{1}{{\left(2\pi \right)}^{n/2}\det{\left(R\right)}^{1/2}e^{\frac{1}{2}m^{T}R^{-1}m}}e^{-\left[-m^{T}R^{-1}z+\frac{1}{2}z^{T}R^{-1}z\right]}\\ p_{\left(m,R\right)}(z)=p_{\hat{\xi }}(\xi )=\frac{1}{Z}e^{-\langle \beta ,\xi \rangle }\text{ with }\xi =\left[\begin{array}{c} z\\ zz^{T} \end{array}\right]\text{ , }\hat{\xi }=\left[\begin{array}{c} E\left[z\right]\\ E\left[zz^{T}\right] \end{array}\right]=\left[\begin{array}{c} m\\ R+mm^{T} \end{array}\right]\\ \text{and }\beta =\left[\begin{array}{c} -R^{-1}m\\ \frac{1}{2}R^{-1} \end{array}\right]=\left[\begin{array}{c} a\\ H \end{array}\right]\text{ where }\langle \beta ,\xi \rangle =a^{T}z+z^{T}Hz=Tr\left[za^{T}+zz^{T}H^{T}\right]\\ \text{with }\log\left(Z\right)=\frac{n}{2}\log(2\pi )+\frac{1}{2}\log\det(R)+\frac{1}{2}m^{T}R^{-1}m\text{ and }S(\hat{\xi })=\langle \hat{\xi },\beta \rangle -\Phi (\beta )\\ \hat{\xi }=\Theta (\beta )=\frac{\partial \Phi (\beta )}{\partial \beta }\text{ and }\beta =\Theta^{-1}(\hat{\xi })\text{ with }\Phi (\beta )=-\log\psi_{\Omega }(\beta )=-\log\int_{\Omega^{*}}e^{-\langle \beta ,\xi \rangle }d\xi \\ Fisher\text{: }I(\beta )=\frac{\partial^{2}\log\psi_{\Omega }(\beta )}{\partial \beta^{2}}=E\left[\frac{\partial \log p_{\beta }(\xi )}{\partial \beta }{\frac{\partial \log p_{\beta }(\xi )}{\partial \beta }}^{T}\right]=E\left[\left(\xi -\hat{\xi }\right){\left(\xi -\hat{\xi }\right)}^{T}\right] \end{array}$$

We can observe in previous equations that classical multivariate Gaussian density, classically expressed by $p_{\left(m,R\right)}(z)=\frac{1}{{\left(2\pi \right)}^{n/2}\det{\left(R\right)}^{1/2}}e^{-\frac{1}{2}{\left(z-m\right)}^{T}R^{-1}(z-m)}$ could be rewritten in a new parameterization in a Gibbs density form $p_{\hat{\xi }}(\xi )=\frac{1}{Z}e^{-\langle \beta ,\xi \rangle }$ with tensor variable $\xi =\left[\begin{array}{c} z\\ zz^{T} \end{array}\right]$, where $\hat{\xi }=E\left[\xi \right]=\left[\begin{array}{c} m\\ R+mm^{T} \end{array}\right]$ and tensor parameterization $\beta =\left[\begin{array}{c} -R^{-1}m\\ \frac{1}{2}R^{-1} \end{array}\right]=\left[\begin{array}{c} a\\ H \end{array}\right]$ with the following definition of duality braket given by $\langle \beta ,\xi \rangle =a^{T}z+z^{T}Hz=Tr\left[za^{T}+zz^{T}H^{T}\right]$ also written in the initial parameterization $\langle \beta ,\xi \rangle =-m^{T}R^{-1}z+\frac{1}{2}z^{T}R^{-1}z=Tr\left[-zm^{T}R^{-1}+\frac{1}{2}zz^{T}R^{-1}\right]$. To understand the meaning of these tensors, we can consider them as homeomorph to the following respective matrices $\xi =\left[\begin{array}{cc} zz^{T} & z\\ 0_{1\times n} & 0 \end{array}\right]$, $\hat{\xi }=\left[\begin{array}{cc} R+mm^{T} & m\\ 0_{1\times n} & 0 \end{array}\right]$ and $\beta =\left[\begin{array}{cc} \frac{1}{2}R^{-1} & -R^{-1}m\\ 0_{1\times n} & 0 \end{array}\right]$ with $\langle \beta ,\xi \rangle =Tr\left[\beta \xi^{T}\right]$ (see [91] for more details).

*Z* is the classical normalization constant that is equal to $log\left(Z\right)=\frac{n}{2}\log(2\pi )+\frac{1}{2}\log\det(R)+\frac{1}{2}m^{T}R^{-1}m$. In this new parameterization, we can express the entropy by Legendre transform $S(\hat{\xi })=\langle \hat{\xi },\beta \rangle -\Phi (\beta )$ of Massieu characteristic function $\Phi (\beta )=-\log\psi_{\Omega }(\beta )=-\log\int_{\Omega^{*}}e^{-\langle \beta ,\xi \rangle }d\xi$ (minus logarithm of partition function $\psi_{\Omega }(\beta )=\int_{\Omega^{*}}e^{-\langle \beta ,\xi \rangle }d\xi$), with the Souriay (Planck) geometric temperature given by $\beta =\Theta^{-1}(\hat{\xi })$ where the function Θ(.) is the inverse of the function given by $\hat{\xi }=\Theta (\beta )=\frac{\partial \Phi (\beta )}{\partial \beta }$ (the temperature is also given by $\beta =\frac{\partial S(\hat{\xi })}{\partial \hat{\xi }}$ given by Lagendre transform; where we recover classical definition of entropy by Clausius $dS=\frac{dQ}{T}$ when $\beta =\frac{1}{T}$ and $\hat{\xi }=Q$ heat). We can also defined Fisher metric of information geometry by $I(\beta )=\frac{\partial^{2}\log\psi_{\Omega }(\beta )}{\partial \beta^{2}}$ or $I(\beta )=-E\left[\frac{\partial^{2}\log p_{\beta }(\xi )}{\partial \beta^{2}}\right]=E\left[\frac{\partial \log p_{\beta }(\xi )}{\partial \beta }{\frac{\partial \log p_{\beta }(\xi )}{\partial \beta }}^{T}\right]=E\left[\left(\xi -\hat{\xi }\right){\left(\xi -\hat{\xi }\right)}^{T}\right]$. From this development, we can observe that classical multivariate Gaussian Density $p_{\hat{\xi }}(\xi )=\frac{1}{Z}e^{-\langle \beta ,\xi \rangle }$ is a maximum entropy Gibbs density of 1st order with respect to the tensorial variable $\hat{\xi }=E\left[\xi \right]=\left[\begin{array}{c} m\\ R+mm^{T} \end{array}\right]$. Classically Gaussian density is considered as a maximum entropy Gibbs density of 2nd order where $p_{\left(m,R\right)}(z)=\frac{1}{{\left(2\pi \right)}^{n/2}\det{\left(R\right)}^{1/2}}e^{-\frac{1}{2}{\left(z-m\right)}^{T}R^{-1}(z-m)}$ is solution to $-\int p_{\left(m,R\right)}(z)\log p_{\left(m,R\right)}(z)dz$ under the constraints that first two moments are known $m=\int z.p_{\left(m,R\right)}(z)dz$ and $R=\int \left(z-m\right){\left(z-m\right)}^{T}.p_{\left(m,R\right)}(z)dz$. The question is then, could we define a Gaussian density of higher order?

We have seen that Souriau has replaced classical maximum entropy approach by replacing Lagrange parameters by only one geometric “temperature vector” as element of Lie algebra. In parallel, Ingarden has introduced second and higher order temperature of the Gibbs state that could be extended to Souriau’s theory of thermodynamics. The question is then, how to extend the Souriau model to define an higher order Lie groups thermodynamics. For this purpose, we propose to consider multi-symplectic geometry and more particularly poly-symplectic geometry [115]. The variational problems generalization with several variables was developed by Volterra in two papers [116,117] where two different generalizations of the Hamilton system of equations are introduced. In parallel, De Donder [53] has also studied this approach in a geometrical framework based on Elie Cartan’s idea of invariant structure with no dependence to local coordinates and based on affine multisymplectic manifold. We can also formalize the multisymplectic geometry with an extension of the Poincaré-Cartan invariant integrals. Frédéric Hélein has observed the fact that different theories could cohabitate was considered jointly by Lepage [54], Dedecker [118,119] and Kijowski [92,93,94]. The Lepage–Dedecker theory was developed by Hélein [120], and the modern formulation using the multisymplectic (*n* + 1)-form as the fundamental structure of the theory starts with Kijowski’s papers. The geometrical multisymplectic approach uses the generalized Legendre correspondence introduced by Lepage and Dedecker and Hamiltonian formalism developed by Hélein [55]. We can also make references to poly-symplectic formulation of physical systems by Carathéodory [121] and Weyl [122].

Among all multi-symplectic models, the more natural multi-valued one that preserve the notion of (poly-)moment map has been initiated by Günther based on n-symplectic model. Günther has shown that the symplectic structure on the phase space remains true, if we replace the symplectic form by a vector valued form, that is called poly-symplectic. The Günther formalism is based on the notion of a poly-symplectic form, which is a vector valued generalization of symplectic forms. Hamiltonian formalism for multiple integral variational problems and field theory is presented in a global geometric setting. Günther has introduced in this poly-symplectic formalism: Hamiltonian equations, canonical transformations, Lagrange systems, symmetries, Field theoretic moment mappings, a classification of G-homogeneous field theoretic systems on a generalization of coadjoint orbits.

Günther has defined six conditions for a multidimensional Hamiltonian formalism:

- *C0*: For each field system, an evolution space can be constructed, which describes the states of the system completely.
- *C1*: The evolution space carries a geometric structure, which assigns to each function (Hamiltonian density) its Hamiltonian equations.
- *C2*: The geometry of the evolution space gives ‘canonical transformations’, i.e., the general symmetry group of a system independently of the choice of Hamiltonian density.
- *C*3: The formalism is covariant, i.e., no special coordinates or coordinate systems on the parameter space are used to construct the Hamiltonian equations.
- *C4*: There is an equivalence between regular Lagrange systems and certain (regular) Hamiltonian systems.
- *C5*: For one dimensional parameter space the theory reduces to the ordinary Hamiltonian formalism on symplectic manifolds in classical mechanics.

Günther has observed that Hamiltonian field theory by Marsden is not covariant, because C3 is not verified and causes problems in relativistic theories, and by the multisymplectic approach by Tulczyjew, based on the general theory by Dedecker, does not satisfy C1 and C2.

The key idea of Günther for this generalized Hamiltonian formalism is to replace the symplectic form in classical mechanics by a vector valued, so called poly-symplectic form with the property that:

- the evolution space of a classical field will appear as the dual of a jet bundle, which carries naturally a polysymplectic structure.
- canonical transformations are bundle isomorphisms leaving this poly-symplectic form invariant.

The polysymplectic approach recovers all classical results also generalize the Noether theorem based on canonical transformations and preserve the existence of momentum mappings. Christian Günther’s work was inspired by the symplectic formulation of classical mechanics by Souriau and by the work of Edelen [52,123] and Rund [124] on a local Hamiltonian formulation of field theory. Edelen’s work is a coordinate version of the local polysymplectic approach of Günther.

Initiated by Gunther [48,49] based on n-symplectic model [50,51], it has been shown that the symplectic structure on the phase space remains true, if we replace the symplectic form by a vector valued form, that is called polysymplectic.

In Günther’s poly-symplectic model, we set: P: space of field values , ϕ:U→P and we consider the bundle of linear maps from *R^n^* into the tangent spaces of *P*:

(118) $$I^{n}P≅Hom\left(R^{n},TP\right)≅TP⊗R^{n*}\;$$

The base of *R^n^* is interpreted as n-tangent υectors of *M*, there is the isomophy:

(119) $$I^{n}P≅{⊕}_{1}^{n}TP\;$$

The natural projection is given by:

(120) $$\tau_{P}^{n}:I^{n}P\to P\;$$

The cojet space $Hom\left(R^{n},TP\right)$ carries a natural *R^n^*-valued:

- one-form: $\Theta_{0}$ (canonical one-form):
  (121) $$\Theta_{0}=\sum_{i=1}^{n}p_{i}dq⊗\frac{\partial }{\partial x_{i}}\;$$
- two-form: $\Omega_{0}=-d\Theta_{0}$ closed & non-degenerate (canonical polysymplectic form)
  (122) $$\Omega_{0}=\sum_{i=1}^{n}dq\wedge dp_{i}⊗\frac{\partial }{\partial x_{i}}\;$$

**Definition** **2.**

- *A closed nondegenerate R^n^-valued two-form Ω on a manifold M is called a polysymplectic form. The pair (M, Ω) is a polysymplectic manifold.*
- *A polysymplectic form Ω on a manifold M is called a standard form iff M has an atlas of canonical charts for Ω, i.e., charts in which locally Ω is written as the canonical evaluation form on P x Lin (P,R^n^). (M, Ω) is called a standard polysymplectic manifold.*

The classification of symplectic homogeneous spaces by coadjoint orbits by Souriau belong to the major achievements in Hamiltonian mechanics. Günther has extended these results to polysymplectic manifolds. Let Ad:G×LG→LG be the adjoint action. We denote by $Ad^{n}$ induced action on $Lin\left(R^{n},LG\right)$:

(123) $$\begin{array}{l} Ad_{g}^{n}:G\times Lin\left(R^{n},LG\right)\to Lin\left(R^{n},LG\right)\\ \;Ad_{g}^{n}(f)(x)=Ad_{g}\left(f(x)\right)\;,\;f\in Lin\left(R^{n},LG\right),x\in R^{n},g\in G \end{array}$$

The dual of $Ad^{n}$ is denoted by $Ad_{g}^{(n)*}$:

(124) $$Ad^{\#}:G\times LG^{*}⊗R^{n}\to LG^{*}⊗R^{n}\;$$

**Corollary 1.** **[Günther Corollary]** *Let the moment map*$J^{\left(n\right)}:M\to Lin\left(LG,R^{n}\right)=LG^{*}⊗R^{n}$*, there is a smooth map*$\theta^{\left(n\right)}$:

(125) $$\theta^{\left(n\right)}:G\to LG^{*}⊗R^{n}\;,\;\theta^{\left(n\right)}(g)=J^{\left(n\right)}\left(\Phi_{g}(x)\right)-Ad_{g}^{(n)*}\left(J^{\left(n\right)}(x)\right)\;$$

*with the following properties:*$\theta^{\left(n\right)}$ is a 1-cocyle for all g,h∈G then:

(126) $$\theta^{\left(n\right)}\left(gh\right)=Ad_{h}^{(n)*}\left(\theta^{\left(n\right)}(g)\right)+\theta^{\left(n\right)}(h)\;$$

**Theorem** **4.** **[Günther Theorem (Vector-Valued Extension of Souriau Theorem)]** *The map:*

(127) $$\begin{array}{l} a:G\times LG^{*}⊗R^{n}\to G\times LG^{*}⊗R^{n}\\ a\left(g,\eta \right)=Ad_{g}^{(n)*}\eta +\theta^{\left(n\right)}(g) \end{array}$$

*is an affine operation of*G*on*$LG^{*}⊗R^{n}$*, and commutes for all*g∈G.

This extension by Günther defines an action of *G* over $g^{*}\times \overset{\left(n\right)}{\ldots }\times g^{*}$ called n-coadjoint action:

**Definition** **3.** (128) $$\begin{array}{ll} Ad_{g}^{*(n)}: & G\times \left(g^{*}\times \overset{\left(n\right)}{\ldots }\times g^{*}\right)\to g^{*}\times \overset{\left(n\right)}{\ldots }\times g^{*}\\ & g\times \mu_{1}\times \ldots \times \mu_{n}\mapsto Ad_{g}^{*(n)}\left(\mu_{1},\ldots ,\mu_{n}\right)=\left(Ad_{g}^{*}\mu_{1},\ldots ,Ad_{g}^{*}\mu_{n}\right) \end{array}$$

*Let*$\mu =\left(\mu_{1},\ldots ,\mu_{n}\right)$*a poly-momentum, element of*$g^{*}\times \overset{\left(n\right)}{\ldots }\times g^{*}$, we can define a n-coadjoint orbit$O_{\mu }=O_{\left(\mu_{1},\ldots ,\mu_{n}\right)}$*at the point*μ*, for which the canonical projection*${\mathrm{Pr}}_{k}:g^{*}\times \overset{\left(n\right)}{\ldots }\times g^{*}\to g^{*}\;,\;\left(\nu_{1},\ldots ,\nu_{n}\right)\mapsto \nu_{k}$*induces a smooth map between the n-coadjoint orbit*$O_{\mu }$*and the coadjoint orbit*$O_{\mu_{k}}$*:*$\pi_{k}:O_{\mu }=O_{\left(\mu_{1},\ldots ,\mu_{n}\right)}\to O_{\mu_{k}}$*that is a surjective submersion with*$\overset{n}{\underset{k=1}{\cap }}KerT\pi_{k}=\left\{0\right\}$.

**Proposition** **1.** Extending Souriau’s approach, equivariance of poly-moment is a unique action a(.,.) of the Lie group G *on* $g^{*}\times \overset{\left(n\right)}{\ldots }\times g^{*}$ *for which the polymoment map* $J^{\left(n\right)}=\left(J^{1},\ldots ,J^{n}\right):M\to g^{*}\times \overset{\left(n\right)}{\ldots }\times g^{*}$ *verifies* x∈M *and* g∈G:

(129) $$J^{\left(n\right)}\left(\Phi_{g}(x)\right)=a(g,J^{\left(n\right)}(x))=Ad_{g}^{*(n)}\left(J^{\left(n\right)}(x)\right)+\theta^{\left(n\right)}(g)\;$$

*with:*

(130) $$Ad_{g}^{*(n)}\left(J^{\left(n\right)}(x)\right)=\left(Ad_{g}^{*}J^{1},\ldots ,Ad_{g}^{*}J^{n}\right)\;$$

*and:*

(131) $$\theta^{\left(n\right)}(g)=\left(\theta^{1}(g),\ldots ,\theta^{n}(g)\right)\;$$

$\theta^{\left(n\right)}(g)$ *is a poly-symplectic one-cocycle.*

**Definition** **4.** *We define a poly-symplectic two-cocycle*${\tilde{\Theta }}^{\left(n\right)}=\left({\tilde{\Theta }}^{1},\ldots ,{\tilde{\Theta }}^{n}\right)$*with*

(132) $${\tilde{\Theta }}^{k}(X,Y)=\langle \Theta^{k}(X),Y\rangle =J_{\left[X,Y\right]}^{k}-\left\{J_{X}^{k},J_{Y}^{k}\right\}\;$$

where:

(133) $$\Theta^{k}(X)=T_{e}\theta^{k}\left(X(e)\right)\;$$

*Finally, we propose to define the poly-symplectic Souriau-Fisher metric.*

**Definition** **5.** (134) gβ([β,Z1],Z2)=diag[Θ˜βk(Z1,Z2)]k , ∀Z1∈g,∀Z2∈Im(adβ(.)),β=(β1,…,βn) 

*with*

(135) $${\tilde{\Theta }}_{\beta_{k}}\left(Z_{1},Z_{2}\right)=-\frac{\partial \Phi \left(\beta_{1},\ldots ,\beta_{n}\right)}{\partial \beta_{k}}={\tilde{\Theta }}^{k}\left(Z_{1},Z_{2}\right)+\langle Q_{k},ad_{Z_{1}}(Z_{2})\;\rangle \;$$

*is a poly-symplectic extension of Souriau-Fisher Metric*.

Compared to the Souriau model, heat is replaced by previous polysymplectic model:

(136) $$Q=\left(Q_{1},\ldots ,Q_{n}\right)\in g^{*}\times \overset{\left(n\right)}{\ldots }\times g^{*}\;\;\mathrm{with}\;Q_{k}=\frac{\partial \Phi (\beta_{1},\ldots ,\beta_{n})}{\partial \beta_{k}}=\frac{\int_{M}U^{⊗k}(\xi ).e^{-\sum_{k=1}^{n}\langle \beta_{k},U^{⊗k}(\xi )\rangle }d\omega }{\int_{M}e^{-\sum_{k=1}^{n}\langle \beta_{k},U^{⊗k}(\xi )\rangle }d\omega }\;$$

**Proposition** **2.** *The characteristic function:*

(137) $$\Phi (\beta_{1},\ldots ,\beta_{n})=-\log\int_{M}e^{-\sum_{k=1}^{n}\langle \beta_{k},U^{⊗k}(\xi )\rangle }d\omega \;$$

*exists.*

**Proof.**  We extrapolate Souriau’s results, who proved in [1,2] that $\int_{M}U^{⊗k}(\xi ).e^{-\langle \beta_{k},U^{⊗k}(\xi )\rangle }d\omega$ is locally normally convergent using multi-linear norm $\left\|U^{⊗k}\right\|=\underset{U}{Sup}{\langle E,U\rangle }^{k}$ and where $U^{⊗k}=U⊗\overset{\left(k\right)}{U}\ldots ⊗U\;$ is defined as a tensorial product [43]. □

Entropy is defined by the Legendre transform of the Souriau-Massieu characteristic function:

**Definition** **6.** *The poly-entropy is given by Legendre transform of the poly-symplectic characteristic function:*

(138) $$S\left(Q_{1},\ldots ,Q_{n}\right)=\sum_{k=1}^{n}\langle \beta_{k},Q_{k}\rangle -\Phi (\beta_{1},\ldots ,\beta_{n})\;\mathrm{where}\;\beta_{k}=\frac{\partial S(Q_{1},\ldots ,Q_{n})}{\partial Q_{k}}\;$$

*The Gibbs density could be then extended with respect to high order temperatures.*

**Definition** **7.** *Gibbs density is defined as the maximum entropy density of poly-Entropy:*

(139) $$p_{Gibbs}(\xi )=e^{\Phi \left(\beta_{1},\ldots ,\beta_{n}\right)-\sum_{k=1}^{n}\langle \beta_{k},U^{⊗k}(\xi )\rangle }=\frac{e^{-\sum_{k=1}^{n}\langle \beta_{k},U^{⊗k}(\xi )\rangle }}{\int_{M}e^{-\sum_{k=1}^{n}\langle \beta_{k},U^{⊗k}(\xi )\rangle }d\omega }\;$$

## 9. Conclusions and Possible Extensions

**Figure 9:**
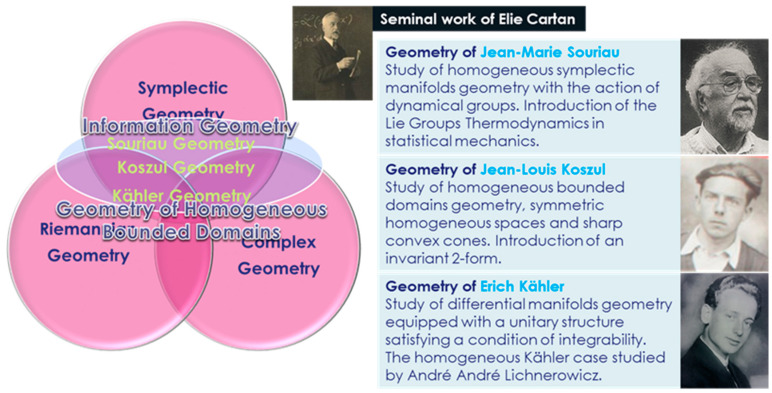
Three Sources of Geometric Structures for Information and Heat.

We have introduced contextures of geometric theory of information and heat based on Souriau’s approach, but information geometry is at the interface between different geometries. First, information geometry is at the intersection between “Riemannian geometry”, “complex geometry” and “symplectic geometry”. Based on seminal work of Cartan on homogeneous domains and other works [125,126,127,128], information geometry is jointly founded by (see Figure 9):

- *Geometry of Jean-Marie Souriau*: Study of homogeneous symplectic manifolds geometry with the action of dynamical groups. Introduction of the Lie groups thermodynamics in statistical mechanics [43,44].
- *Geometry of Jean-Louis Koszul*: Study of homogeneous bounded domains geometry, symmetric homogeneous spaces and sharp convex cones. Introduction of an invariant 2-form [9,10,11,97,98,129].
- *Geometry of Erich Kähler*: Study of differential manifolds geometry equipped with a unitary structure satisfying a condition of integrability. The homogeneous Kähler case studied by André Lichnerowicz [130].

We have extended Souriau’s Lie groups thermodynamics by a vector-valued model based on poly-symplectic geometry, introducing higher order Souriau-Gibbs density with higher order Souriau temperatures, and elements of Lie algebra. This model preserves all contextures of Souriau’s thermodynamics with covariance of Gibbs density with respect to dynamical groups in physics. Poly-moment maps are compliant with the Noether theorem generalization in vector-valued cases.

**Figure 10:**
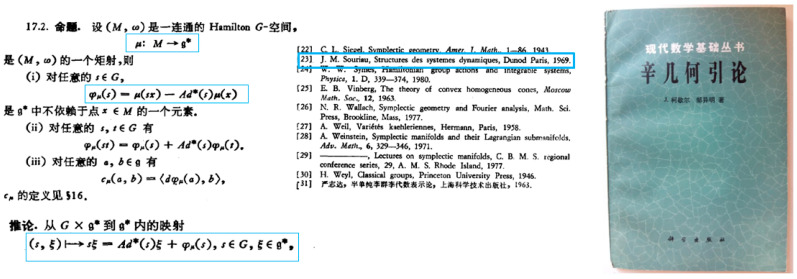
Koszul Lecture on “*Introduction of Symplectic Geometry*” where the Souriau model of non-equivariance is developed.

The Jean-Marie Souriau model and equations were extensively studied in the Koszul Lecture given in China in 1986 “*Introduction to Symplectic Geometry*”, in Chinese (see Figure 10). This book should be translated in English in 2019. Chuan Yu Ma has written on the Koszul book: “*This beautiful, modern book should not be absent from any institutional library. …. During the past eighteen years there has been considerable growth in the research on symplectic geometry. Recent research in this field has been extensive and varied. This work has coincided with developments in the field of analytic mechanics. Many new ideas have also been derived with the help of a great variety of notions from modern algebra, differential geometry, Lie groups, functional analysis, differentiable manifolds and representation theory. [Koszul’s book] emphasizes the differential-geometric and topological properties of symplectic manifolds. It gives a modern treatment of the subject that is useful for beginners as well as for experts.*”

We have seen that in geometrical mechanics, the Galileo group related to classical mechanics:

(140) $$\left[\begin{array}{c} \vec{x}\prime \\ t\prime \\ 1 \end{array}\right]=\left[\begin{array}{ccc} R & \vec{u} & \vec{w}\\ 0 & 1 & e\\ 0 & 0 & 1 \end{array}\right]\left[\begin{array}{c} \vec{x}\\ t\\ 1 \end{array}\right]\;,R\in SO(3),\;\vec{u},\vec{w}\in R^{3},e\in R\;$$

and its central extension given by the Bargman group:

(141) $$\left[\begin{array}{cccc} R & \vec{u} & 0 & \vec{w}\\ 0 & 1 & 0 & e\\ -{\vec{u}}^{t}R & -\frac{{\left\|\vec{u}\right\|}^{2}}{2} & 1 & f\\ 0 & 0 & 0 & 1 \end{array}\right]\;$$

and Poincaré group in relativity. We then observe, that affine group or its sub-groups are at cornerstone of different disciplines such as:

- In robotics, the special Euclidean group SE(3) which is the homogeneous Galileo group (robotics also consider the group of similitudes SIM(3)):
  (142) $$\left[\begin{array}{c} Z\prime \\ 1 \end{array}\right]=\left[\begin{array}{cc} \Omega & t\\ 0 & 1 \end{array}\right]\left[\begin{array}{c} Z\\ 1 \end{array}\right]\;,\;\left\{ \begin{array}{l} \Omega \in SO(3)\\ t\in R^{3} \end{array}\right.\;$$
- In information geometry, the general affine group is involved A(n,R) for exponential family:
  (143) $$\left[\begin{array}{c} Z\prime \\ 1 \end{array}\right]=\left[\begin{array}{cc} A & t\\ 0 & 1 \end{array}\right]\left[\begin{array}{c} Z\\ 1 \end{array}\right]\;,\;\left\{ \begin{array}{l} A\in GL(n)\\ t\in R^{n} \end{array}\right.\;$$
  with particular case of Gaussian density, associated by Cholesky factorisation of covariance matrix, where covariance matrix square root is triangular matrix with positive elements on its diagonal (it is a group):
  (144) $$\left[\begin{array}{c} Y\\ 1 \end{array}\right]=\left[\begin{array}{cc} R^{1/2} & m\\ 0 & 1 \end{array}\right]\left[\begin{array}{c} X\\ 1 \end{array}\right]\;,\;\left\{ \begin{array}{l} R^{1/2}\in T_{n}^{+}\;\\ \left(R^{1/2}:\;\mathrm{Cholesky}\;\mathrm{de}\;R\right)\\ m\in R^{n} \end{array}\right.\;$$
- In the study of homogeneous bounded domains, as the simplest one given by Poincaré upper-half plane:
  (145) $$\left[\begin{array}{c} X\prime \\ 1 \end{array}\right]=\left[\begin{array}{cc} a & b\\ 0 & 1 \end{array}\right]\left[\begin{array}{c} X\\ 1 \end{array}\right]\;,\;a\in R_{+}^{*}\;\mathrm{et}\;b\in R\;$$

**Figure 11:**
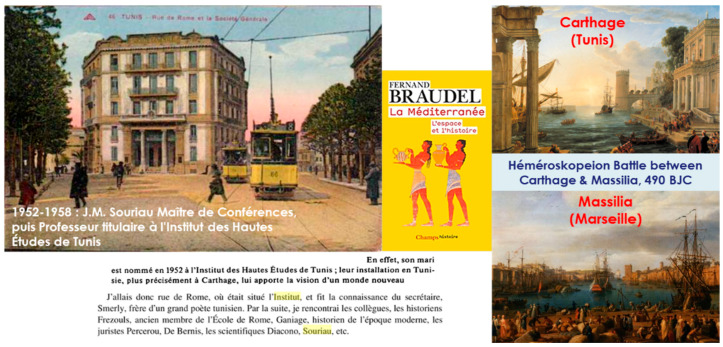
Mediterranean sources of Souriau Book on Structure of Dynamical systems at Carthage and Massilia where souriau wrote this text and theory.

As illustrated in Figure 11, Jean-Marie Souriau developed these models at Carthage in Tunisia and at Marseilles in France during 50’s and 60’s. Jean-Marie Souriau was motivated by group invariance, not only in physics but also in neuroscience. Souriau intuition was highly premonitory, because this neuroscience domain has been developed few decades after by Alain Berthoz at College de France (http://public.weconext.eu/academie-sciences/2017-10-03_5a7/video_id_002/index.html) and by Daniel Bennequin (https://www.youtube.com/watch?v=a-ctwxBpJxE) to study the brain sense of movment. We can read in Souriau’s text the very interesting remarks on geometry and neuroscience:

“*Je me suis dit, à force de rencontrer des groupes, il y a quelque chose de caché là-dessous. La catégorie métaphysique des groupes qui plane dans l’empyrée des mathématiques, que nous découvrons et que nous adorons, elle doit se rattacher à quelque chose de plus proche de nous. En écoutant de nombreux exposés faits par des neurophysiologistes, j’ai fini par apprendre le rôle primitif du déplacement des objets. Nous savons manipuler ces déplacements mentalement avec une très grande virtuosité. Ce qui nous permet de nous manipuler nous-même, de marcher, de courir, de sauter, de nous rattraper quand nous tombons,* etc. *Ce n’est pas vrai seulement pour nous, c’est vrai aussi pour les singes ; ils sont beaucoup plus adroits que nous pour anticiper les résultats d’un déplacement. Pour certaines opérations élémentaires de «lecture», ils vont même dix fois plus vite que nous. Beaucoup de neurophysiologistes pensent qu’il y a une structure spéciale génétiquement inscrite dans le cerveau, le câblage d’un groupe … Lorsque il y un tremblement de terre, nous assistons à la mort de l’Espace. … Nous vivons avec nos habitudes que nous pensons universelles. … La neuroscience s’occupe rarement de la géométrie … Pour les singes qui vivent dans les arbres, certaines propriétés du groupe d’Euclide sont mieux câblées dans leurs cerveaux.”* (In Engish: *“I said to myself, because of meeting groups everywhere, there is something hidden there. The metaphysical category of groups that hovers in the empyrean of mathematics, which we discover and adore, must be connected with something closer to us. Listening to many presentations by neurophysiologists, I ended up learning the primitive role of moving objects. We know how to manipulate these movements mentally with great virtuosity. That allows us to manipulate ourselves, to walk, run, jump, catch up when we fall, and so on. This is not true only for us, it is true also for monkeys; they are much more adroit than we are to anticipate the results of a trip. For some basic “reading” operations, they are even ten times faster than us. Many neurophysiologists think that there is a special structure genetically inscribed in the brain, the wiring of a group… When there is an earthquake, we witness the death of Space. … We live with our habits that we think universal. … Neuroscience rarely deals with geometry … For monkeys living in trees, some of Euclid’s group properties are better wired in their brains*.)

Our new research directions will concern extension of “*Le Hasard et la Courbure (Randomness and Curvature)*” (title of Yann Ollivier HDR), that we have synthetized in Souriau-Fisher metric to “*Le Hasard et la Torsion (Randomness and Torsion)*” based on Elie Cartan works founded on Cosserats brothers model of elasticity [125,126,127,131].

“*Il est une Cosmologie avec laquelle la Thermodynamique générale présente une analogie non-méconnaissable; cette Cosmologie, c’est la Physique péripatéticienne … Parmi les attributs de la substance, la Physique péripatéticienne confère une égale importance à la catégorie de la quantité et à la catégorie de la qualité; or, par ses symboles numériques, la Thermodynamique générale représente également les diverses grandeurs des quantités et les diverses intensités des qualités. Le mouvement local n’est, pour Aristote, qu’une des formes du mouvement général, tandis que les Cosmologies cartésienne, atomistique et newtonienne concordent en ceci que le seul mouvement possible est le changement de lieu dans l’espace. Et voici que la Thermodynamique générale traite, en ses formules, d’une foule de modifications telles que les variations de températures, les changements d’état électrique ou d’aimantation, sans chercher le moins du monde à réduire ces variations au mouvement local*”

—Pierre Duhem—La théorie Physique: son objet, sa structure [132].

“*Pour la théorie de la connaissance mais aussi pour les sciences est fondamentale la notion de perspective. Or, les expériences faites dans la géométrie algébriques, dans la théorie des nombres, et dans l’algèbre abstraite m’induisent à tenter une formulation mathématique de cette notion pour surmonter ainsi au moyen de raisonnements d’origine géométrique la géométrie. Il me semble en effet, que la tendance vers l’abstraction observée dans les mathématiques d’aujourd’hui, loin d’être l’ennemi de l’intuition ait le sens profond de quitter l’intuition pour la faire renaitre dans une alliance entre «esprit de géométrie» et «esprit de finesse», alliance rendue possible par les réserves énormes des mathématiques pures dont Pascal et Goethe ne pouvaient pas encore se douter*”

—Erich Kähler—Sur la théorie des corps purement algébriques, 1952.

## Figures and Tables

**Figure A1:**
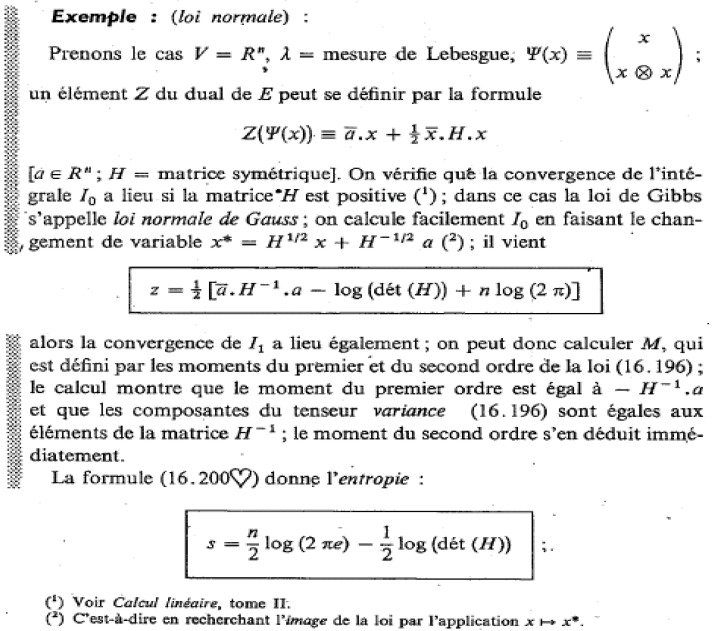
Introduction of potential function for multivariate Gaussian law in Souriau book.

**Figure A2:**
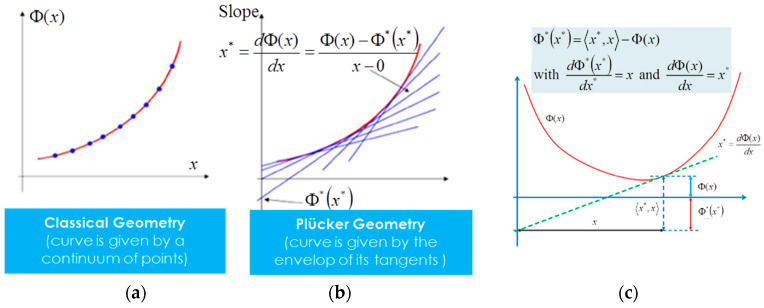
Legendre Transform and duality. (**a**) Classical Geometry; (**b**) Plücker Geometry; (**c**) Legendre Transform.

**Figure A3:**
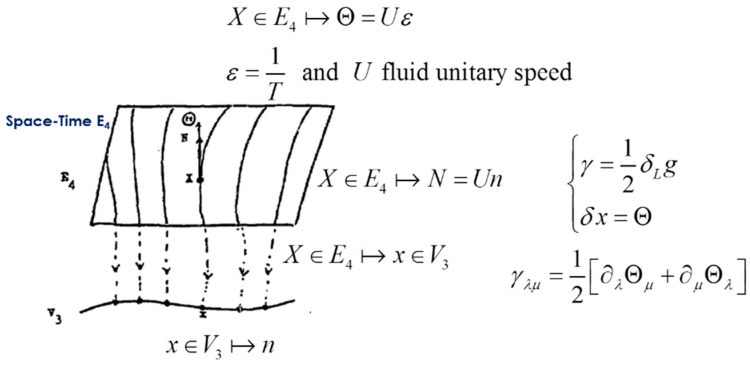
Souriau’s model of Thermodynamics of Continua.

## Appendix A. Günther’s Polysymplectic Model

We recall in this appendix, a synthesis of Christian Günther Poly-symplectic model with his initial notation [48].

We set:

(A1) $$\begin{array}{l} Q:\;\mathrm{space}\;\mathrm{of}\;\mathrm{field}\;\mathrm{values}\\ \varphi :U\to Q \end{array}$$

The bundle of linear maps from *R^n^* into the tangent spaces of *Q*.

(A2) $$I^{n}Q≅Hom\left(R^{n},TQ\right)≅TQ⊗R^{n*}\;$$

If a base of *R^n^* is chosen, can also be interpreted as *n*-tangent υectors of *Q*, there is the isomophy:

(A3) $$I^{n}Q≅{⊕}_{1}^{n}TQ\;$$

The natural projection is given by:

(A4) $$\tau_{Q}^{n}:I^{n}Q\to Q\;$$

In analogy to the canonical forms on the cotangent bundle, the cojet space $Hom\left(R^{n},TQ\right)$ carries a natural *R^n^*-valued:

- one-form: $\Theta_{0}$ (canonical one-form)
- two-form: $\Omega_{0}=-d\Theta_{0}$ closed & non-degenerate (canonical polysymplectic form)

In the natural bundle coordinates the canonical forms on $Hom\left(R^{n},TQ\right)$ have the local representation:

(A5) $$\Theta_{0}=\sum_{i=1}^{n}p_{i}dq⊗\frac{\partial }{\partial x_{i}}\;$$

(A6) $$\Omega_{0}=\sum_{i=1}^{n}dq\wedge dp_{i}⊗\frac{\partial }{\partial x_{i}}\;$$

Following diffeomorphism leaves invariant one and two forms:

(A7) $$\begin{array}{l} f:Q\to Q\;\mathrm{and}\;I^{n*}f:Hom\left(TQ,R^{n}\right)\to Hom\left(TQ,R^{n}\right)\\ {\left(I^{n*}f\right)}^{*}\Theta_{0}=\Theta_{0}\;\mathrm{and}\;{\left(I^{n*}f\right)}^{*}\Omega_{0}=\Omega_{0} \end{array}$$

**Definition** **A1.** *A closed nondegenerate R^n^-valued two-form Ω on a manifold M is called a polysymplectic form. The pair (M, Ω) is a polysymplectic manifold*.

The classification of linear polysymplectic forms is not trivial, because two polysymplectic forms are not necessarily locally equivariant.

**Definition** **A2.** *A polysymplectic form Ω on a manifold M is called a standard form iff M has an atlas of canonical charts for Ω, i.e., charts in which locally Ω is written as the canonical evaluation form on Q x Lin (Q,R^n^). (M, Ω) is called a standard polysymplectic manifold*.

The polysymplectic structure provides the procedure which assigns to a function on *M*, the Hamiltonian, its associated Hamiltonian equations. Let (*M, Ω*) a polysymplectic manifold:

(A8) $$\left\{ \begin{array}{ll} \Omega^{b}: & TM\to Hom\left(TM,R^{n}\right)\\ & w_{m}\mapsto \Omega_{\left(v_{m}\right)}^{b}(w_{m})=\Omega \left(v_{m},w_{m}\right) \end{array}\right.\;\mathrm{and}\;\left\{ \begin{array}{l} \Omega^{\#}:Hom\left(TM,R^{n}\right)\to T^{*}M\\ \;X_{m}\mapsto \Omega^{\#}\left(X_{m}\right)=tr\left(\Omega^{b}\circ X_{m}\right)\\ \mathrm{with}\;tr\left(\Omega^{b}\circ X_{m}\right).v_{m}=-tr\left(\Omega^{b}(v_{m})\circ X_{m}\right) \end{array}\right.\;$$

An affine sub bundle of $Hom\left(R^{n},TQ\right)$ is defined by:

(A9) $$\Omega^{\#-1}\left(dH\right)=\left\{X_{m}\in Hom\left(R^{n},TQ\right)/\Omega^{\#}\left(X_{m}\right)=dH(m)\right\}\;$$

**Definition** **A3.**
$\Omega^{\#-1}\left(dH\right)$ *is called the system of Hamiltonian partial differential equations associated with the Hamiltonian function H. A smooth map* ψ:U→M *is a solution of* $\Omega^{\#-1}\left(dH\right)$ *iff:*

(A10) $$T_{u}\psi \in \Omega^{\#-1}\left(dH\left(\psi (u)\right)\right)\forall u\in U\;$$

**Theorem** **A1.**
*Let (M, Ω) be a standard polysymplectic manifold, (p,q) canonical coordinates for Ω on M, and H a Hamiltonian function. A smooth map* ψ:U→M *is a solution of* $\Omega^{\#-1}\left(dH\right)$ *iff in canonical coordinates:*

(A11) $$trdp(u)=-\frac{\partial H}{\partial q}\left(\psi (u)\right)\;\mathrm{and}\;Dq(u)=\frac{\partial H}{\partial p}\left(\psi (u)\right)\;$$

*If a base* $e_{1},\ldots ,e_{n}$ *of R^n^ is chosen and* $p(u)=\left(p_{1}(u),\ldots ,p_{n}(u)\right)$ *with respect to this base, then the equations take the form:*

(A12) $$\sum_{i=1}^{n}\frac{\partial p_{i}}{\partial x_{i}}(u)=-\frac{\partial H}{\partial q}\left(\psi (u)\right)\;\mathrm{and}\;\frac{\partial q}{\partial x_{i}}(u)=\frac{\partial H}{\partial p_{i}}\left(\psi (u)\right)\;$$

**Proof.** 

(A13) $$\begin{array}{l} X\left(\psi (u)\right)=D\psi (u)\in Lin\left(R^{n},T_{\psi (u)}M\right)\\ X(m)=X_{q}(m)+X_{p}(m)\;,\;X_{q}(m)\in Lin\left(R^{n},Q\right)\;,\;X_{p}(m)\in Lin\left(R^{n},Lin\left(Q,R^{n}\right)\right)\\ v(m)=\dot{q}(m)+\dot{p}(m)\;,\;\dot{q}(m)\in Q\;,\;\dot{p}(m)\in Lin\left(Q,R^{n}\right) \end{array}$$

(A14) $$\begin{array}{l} \Omega^{\#}(X).v=tr\Omega^{b}\circ X(v)=-tr\Omega^{b}(v)\circ X\\ \Omega^{b}(\dot{q},\dot{p}).(\dot{q},\dot{p})=\dot{p}(\dot{q})\;,\;(\dot{q},\dot{p})\in TM\\ \Omega^{\#}(X).(\dot{q},\dot{p})=-tr\left(X_{p}(\dot{q})-\dot{p}\circ X_{q}\right)=dH(\dot{q},\dot{p})\\ dH=\frac{\partial H}{\partial q}dq+\sum_{i=1}^{n}\frac{\partial H}{\partial p_{i}}dp_{i}\Rightarrow -trX_{p}=\frac{\partial H}{\partial q}\;,\;\frac{\partial H}{\partial p}=X_{q} \end{array}$$

□

**Example** **A1.**
*Consider a scalar field where* $n=4,Q=R\;\mathrm{and}\;M=R\times R^{4}$ *with scalar coordinates* $\left(q,p_{1},\ldots ,p_{4}\right)$

Let $H\left(q,p_{1},\ldots ,p_{4}\right)=\frac{1}{2}\sum_{i=1}^{4}p_{i}^{2}+mq^{2}$ an Hamiltonian on *M*, the canonical polysymplectic form *Ω* is given by:

(A15) $$\Omega =\sum_{i=1}^{4}dq\wedge dp_{i}⊗\frac{\partial }{\partial x_{i}}\;$$

The Hamiltonian equations for a scalar field:

(A16) $$\psi \left(x_{1},\ldots ,x_{4}\right)=\left(q\left(x_{1},\ldots ,x_{4}\right),p_{1}\left(x_{1},\ldots ,x_{4}\right),\ldots ,p_{4}\left(x_{1},\ldots ,x_{4}\right)\right)\;$$

are:

(A17) $$\sum_{i=1}^{4}\frac{\partial p_{i}}{\partial x_{i}}=mq\;\mathrm{and}\;\frac{\partial q}{\partial x_{i}}=p_{i}\;$$

**Definition** **A4.**
*Let (M, Ω) be a polysymplectic manifold,* $\Omega^{\#}(X)=dH$ *,* H *is called an momentum tensor iff*

(A18) trdH=dH 

**Proposition** **A1.**

(A19) $$X\neg \Theta_{0}=0,\;d\left(trL_{X}\Theta_{0}\right)=0\;\mathrm{and}\;trL_{X}\Theta_{0}=-d\left(H-tr\left(X\neg \Theta_{0}\right)\right)\;$$

**Proof.** 

(A20) $$\begin{array}{l} \Theta_{0}=\sum_{i}p_{i}dq⊗\frac{\partial }{\partial x_{i}}\;\mathrm{and}\;X=X_{q}\frac{\partial }{\partial q}+\sum_{i}X_{p_{i}}\frac{\partial }{\partial p_{i}}\\ \Rightarrow X\neg \Theta_{0}=\sum_{i}p_{i}X_{q}⊗\frac{\partial }{\partial x_{i}} \end{array}$$

(A21) $$\begin{array}{l} trL_{X}\Theta_{0}=tr\left(dX\neg \Theta_{0}+X\neg d\Theta_{0}\right)\\ tr\left(dX\neg \Theta_{0}+X\neg d\Theta_{0}\right)=-dH+trdX\neg \Theta_{0} \end{array}$$

□

The classification of symplectic homogeneous spaces by coadjoint orbits by Souriau belong to the major achievements in Hamiltonian mechanics. C. Günther has extend these results to polysymplectic manifolds. Let Ad:G×LG→LG be the adjoint action. We denote by $Ad^{n}$ induced action on $Lin\left(R^{n},LG\right)$:

(A22) $$\begin{array}{ll} Ad^{n}: & G\times Lin\left(R^{n},LG\right)\to Lin\left(R^{n},LG\right)\\ & Ad_{g}^{n}(f)(x)=Ad_{g}\left(f(x)\right)\;,\;f\in Lin\left(R^{n},LG\right),x\in R^{n},g\in G \end{array}$$

The dual of $Ad^{n}$ is denoted by $Ad^{\#}$:

(A23) $$Ad^{\#}:G\times LG^{*}⊗R^{n}\to LG^{*}⊗R^{n}\;,\;Ad_{g}^{\#}(\alpha )=\alpha \circ Ad_{g}^{n}\;$$

(A24) $$\lambda \left(Ad_{g}u\right)=\Lambda_{g}^{*}\left(\lambda (u)\right)\Rightarrow \Lambda_{g}^{*}\lambda^{n}(f)=\lambda^{n}\left(Ad_{g}^{n}f\right)\;\mathrm{for}\;\mathrm{all}\;g\in G,f\in Lin\left(R^{n},LG\right)\;$$

**Proposition A2** **[Günther Proposition].** *Let*Λ:G×M→M*be a strongly polysymplectic group action with momentum map*$\mu :M\to Lin\left(LG,R^{n}\right)=LG^{*}⊗R^{n}$. *Assume*M*is connected. Then the map:*

(A25) $$\begin{array}{l} M\to LG^{*}⊗R^{n}\\ m\mapsto \mu \left(\Lambda_{g}m\right)-Ad_{g}^{\#}\left(\mu (m)\right) \end{array}$$

*is a constant on*M*for all*g∈G.

**Corollary** **A1.** *There is a smooth map*χ:

(A26) $$\chi :G\to LG^{*}⊗R^{n}\;,\;\chi (g)=\mu \left(\Lambda_{g}m\right)-Ad_{g}^{\#}\left(\mu (m)\right)\;$$

*with the following properties:*

- *is a 1-cocyle for all*g,h∈G*then*
  (A27) $$\chi \left(gh\right)=Ad_{h}^{\#}\left(\chi (g)\right)+\chi (h)\;$$
- *bilinear map*φ on LG: $\varphi :=L_{\chi }:LG\to LG^{*}⊗R^{n}\;,\;\varphi :LG\times LG\to R^{n}$ *is a 2 cocycle*
  (A28) φ(u,[v,w])+φ(v,[w,u])+φ(w,[u,v])=0 , ∀u,v,w∈LG 

**Proof.** 

(A29) $$\begin{array}{l} \chi (hg)=\mu \circ \Lambda_{hg}(m)-Ad_{hg}^{\#}\mu (m)\\ \chi (hg)=\mu \circ \Lambda_{g}\left(\Lambda_{h}m\right)-Ad_{g}^{\#}\circ \mu \left(\Lambda_{h}m\right)+Ad_{g}^{\#}\circ \mu \left(\Lambda_{h}m\right)-Ad_{g}^{\#}Ad_{h}^{\#}\circ \mu (m)\\ \chi (hg)=\chi (g)+Ad_{g}^{\#}\left(\chi (h)\right) \end{array}$$

□

**Theorem** **A2.** **[Günther Theorem (Vector-valued extension of Souriau Theorem)]**
*Let* Λ:G×M→M *be a polysymplectic action with momentum map* $\mu :M\to LG^{*}⊗R^{n}$ *. Then the map:*

(A30) $$\begin{array}{l} \Xi :G\times LG^{*}⊗R^{n}\to G\times LG^{*}⊗R^{n}\\ \Xi \left(g,\eta \right)=Ad_{g}^{\#}\eta +\chi (g) \end{array}$$

*is an affine operation of* G *on* $LG^{*}⊗R^{n}$ *, and commutes for all* g∈G *and* μ *is G-equivariant.*

**Proof.** 

(A31) $$\begin{array}{l} \Xi \left(gh,\eta \right)=\chi (gh)+Ad_{gh}^{\#}\eta +\chi (h)+\chi (g)\circ Ad_{h}+Ad_{h}^{\#}\circ Ad_{g}^{\#}\eta \\ \Xi \left(gh,\eta \right)=\chi (h)+Ad_{h}^{\#}\left(\chi (g)+Ad_{g}^{*}h\right)=\Xi \left(h,\Xi \left(g,\eta \right)\right) \end{array}$$

Ξ is an action.

(A32) $$\begin{array}{l} \Xi_{g}\circ \mu (m)=\chi (g)+Ad_{g}^{\#}\circ \mu (m)\\ \Xi_{g}\circ \mu (m)=\mu \left(\Lambda_{g}m\right)-Ad_{g}^{\#}\left(\mu (m)\right)+Ad_{g}^{\#}\mu (m)=\mu \circ \Lambda_{g}(m) \end{array}$$

□

Christian Günther in a never found 1987 paper wrote that “*The mathematical framework developed in this paper is used in a separate publication to provide a rigorous foundation for field theory*”. For a more recent study of Günther’s poly-symplectic model, we make reference to [133].

## Appendix B. Fisher Metric for Multivariate Gaussian Density

We will in the following illustrate information geometry for multivariate Gaussian density:

(A33) $$p_{\hat{\xi }}(\xi )=\frac{1}{{\left(2\pi \right)}^{n/2}\det{\left(R\right)}^{1/2}}e^{-\frac{1}{2}{\left(z-m\right)}^{T}R^{-1}(z-m)}\;$$

If we develop:

(A34) $$\begin{array}{ll} \frac{1}{2}{\left(z-m\right)}^{T}R^{-1}(z-m) & =\frac{1}{2}\left[z^{T}R^{-1}z-m^{T}R^{-1}z-z^{T}R^{-1}m+m^{T}R^{-1}m\right]\\ & =\;\frac{1}{2}z^{T}R^{-1}z-m^{T}R^{-1}z+\frac{1}{2}m^{T}R^{-1}m \end{array}$$

We can write the density as a Gibbs density:

(A35) $$\begin{array}{l} p_{\hat{\xi }}(\xi )=\frac{1}{{\left(2\pi \right)}^{n/2}\det{\left(R\right)}^{1/2}e^{\frac{1}{2}m^{T}R^{-1}m}}e^{-\left[-m^{T}R^{-1}z+\frac{1}{2}z^{T}R^{-1}z\right]}=\frac{1}{Z}e^{-\langle \xi ,\beta \rangle }\\ \xi =\left[\begin{array}{c} z\\ zz^{T} \end{array}\right]\;\mathrm{and}\;\beta =\left[\begin{array}{c} -R^{-1}m\\ \frac{1}{2}R^{-1} \end{array}\right]=\left[\begin{array}{c} a\\ H \end{array}\right]\;\mathrm{with}\;\langle \xi ,\beta \rangle =a^{T}z+z^{T}Hz=Tr\left[za^{T}+H^{T}zz^{T}\right] \end{array}$$

We can then rewrite density with canonical variables:

(A36) $$\begin{array}{l} p_{\hat{\xi }}(\xi )=\frac{1}{\int_{\Omega^{*}}e^{-\langle \xi ,\beta \rangle }.d\xi }e^{-\langle \xi ,\beta \rangle }=\frac{1}{Z}e^{-\langle \xi ,\beta \rangle }\;\mathrm{with}\;\log\left(Z\right)=n\log(2\pi )+\frac{1}{2}\log\det(R)+\frac{1}{2}m^{T}R^{-1}m\\ \xi =\left[\begin{array}{c} z\\ zz^{T} \end{array}\right]\;,\;\hat{\xi }=\left[\begin{array}{c} E\left[z\right]\\ E\left[zz^{T}\right] \end{array}\right]=\left[\begin{array}{c} m\\ R+mm^{T} \end{array}\right]\;,\;\beta =\left[\begin{array}{c} a\\ H \end{array}\right]=\left[\begin{array}{c} -R^{-1}m\\ \frac{1}{2}R^{-1} \end{array}\right]\;\mathrm{with}\;\langle \xi ,\beta \rangle =Tr\left[za^{T}+H^{T}zz^{T}\right]\\ R=E\left[\left(z-m\right){\left(z-m\right)}^{T}\right]=E\left[zz^{T}-mz^{T}-zm^{T}+mm^{T}\right]=E\left[zz^{T}\right]-mm^{T} \end{array}$$

The first potential function (free energy/logarithm of characteristic function) is given by:

(A37) $$\psi_{\Omega }(\beta )=\int_{\Omega^{*}}e^{-\langle \xi ,\beta \rangle }.d\xi \;\mathrm{and}\;\Phi (\beta )=-\log\psi_{\Omega }(\beta )=\frac{1}{2}\left[-Tr\left[H^{-1}aa^{T}\right]+\log\left[{\left(2\right)}^{n}\det H\right]-n\log\left(2\pi \right)\right]\;$$

We verify the relation between the first potential function and moment:

(A38) $$\begin{array}{l} \frac{\partial \Phi (\beta )}{\partial \beta }=\frac{\partial \left[-\log\psi_{\Omega }(\beta )\right]}{\partial \beta }=\int_{\Omega^{*}}\xi \frac{e^{-\langle \xi ,\beta \rangle }}{\int_{\Omega^{*}}e^{-\langle \xi ,\beta \rangle }.d\xi }.d\xi =\int_{\Omega^{*}}\xi .p_{\hat{\xi }}(\xi ).d\xi =\hat{\xi }\\ \frac{\partial \Phi (\beta )}{\partial \beta }=\left[\begin{array}{c} \frac{\partial \Phi (\beta )}{\partial a}\\ \frac{\partial \Phi (\beta )}{\partial H} \end{array}\right]=\left[\begin{array}{c} m\\ R+mm^{T} \end{array}\right]=\hat{\xi } \end{array}$$

The second potential function (Shannon entropy) is given as a Legendre transform of the first one:

(A39) $$\begin{array}{l} S(\hat{\xi })=\langle \hat{\xi },\beta \rangle -\Phi \left(\beta \right)\;\mathrm{with}\;\frac{\partial \Phi (\beta )}{\partial \beta }=\hat{\xi }\;\mathrm{and}\;\frac{\partial S(\hat{\xi })}{\partial \hat{\xi }}=\beta \\ S\left(\hat{\xi }\right)=-\int_{\Omega^{*}}\frac{e^{-\langle \xi ,\beta \rangle }}{\int_{\Omega^{*}}e^{-\langle \xi ,\beta \rangle }.d\xi }\log\frac{e^{-\langle \xi ,\beta \rangle }}{\int_{\Omega^{*}}e^{-\langle \xi ,\beta \rangle }.d\xi }.d\xi =-\int_{\Omega^{*}}p_{\hat{\xi }}(\xi )\log p_{\hat{\xi }}(\xi ).d\xi \end{array}$$

(A40) $$S(\hat{\xi })=-\int_{\Omega^{*}}p_{\hat{\xi }}(\xi )\log p_{\hat{\xi }}(\xi ).d\xi =\frac{1}{2}\left[\log{\left(2\right)}^{n}\det\left[H^{-1}\right]+n\log\left(2\pi .e\right)\right]=\frac{1}{2}\left[\log\det\left[R\right]+n\log\left(2\pi .e\right)\right]\;$$

This remark was made by Jean-Souriau in his book as soon as 1969. He has observed, as illustrated in following Figure that if we take vector with tensor components $\xi =\left(\begin{array}{c} z\\ z⊗z \end{array}\right)$, components of $\hat{\xi }$ will provide moments of the first and second order of the density of probability $p_{\hat{\xi }}(\xi )$. He used this change of variable $z\prime =H^{1/2}z+H^{-1/2}a$, to compute the logarithm of the characteristic function Φ(β) (see Figure A1 extracted from Souriau Book):

## Appendix C. Geometric Definition of Legendre Transform by Chasles as Reciprocal Polar with Respect to a Paraboloid

The Legendre transform plays a central role related to duality and convexity. Adrien-Marie Legendre [102] has introduced the Legendre transform to solve a minimal surface problem given by Monge (Monge requested him to consolidate its proof), with a link to Poncelet duality [103]. Chasles and Darboux interpreted the Legendre transform as reciprocal polar with respect to a paraboloid (re-used by Hadamard and Fréchet in calculus of variations). Before Legendre, Alexis Clairaut introduced a Clairaut Equation that has been developed by Maurice Fréchet to characterize «distinguished densities» (densities with parameters that have covariance matrix reaching the Fréchet-Cramer-Rao Bound) [9].

Legendre Transform transformes one fonction defined by its value in one point in a fonction defined by its tangent, as illustrated in Figure A2.

Darboux gave in his book one interpretation of Chasles: “*Ce qui revient suivant une remarque de M. Chasles, à substituer à la surface sa polaire réciproque par rapport à un paraboloïde*». In the lecture «*Leçons sur le calcul des variations*”, Hadamard, followed by Vessiot, used the reciprocal polar of figurative, and figuratrice. This has also been developed by Belgodère as presented by Cartan on «*Extrémale d’une surface*» [134,135]. Polarity on the plane is a transformation taking points to lines and dually lines to points. A polarity preserves incidence and has degree 2. For a point *P* (that we name the pole) a conic polarity transforms it to its image which is a line *p* (that we name the polar) as follows: from *P* we draw the two tangents to the conic, which touch it in the points *Q*, *R*. If we now connect points *Q*, *R* with a line *p* we obtain the polar line of the pole *P*. A Self-conjugate point *Q* is incident with its polar *q*; that is *Q* lies on *q*.

Geometric interpretation of the Legendre transform by reciprocal polar with respect to a paraboloid is given by the following simple development. First, let’s consider the surface:

(A41) $$z=f(x,y)\;\mathrm{with}\;p=\frac{\partial z}{\partial x}\;\mathrm{and}\;q=\frac{\partial z}{\partial y}\;$$

We consider the equation of the paraboloid:

(A42) $$x^{2}+y^{2}=2z\;$$

Reciprocal polar with respect to paraboloid has coordinates: X,Y,Z

The polar plan with respect to paraboloid of this reciprocal polar Xx+Yy−z−Z=0 should be equal to tangent plan of the surface at point $\left(x_{0},y_{0},z_{0}\right)$:

(A43) $$z-z_{0}=p_{0}\left(x-x_{0}\right)+q_{0}(y-y_{0})\Rightarrow p_{0}x+q_{0}y-z-\left(p_{0}x_{0}+q_{0}y_{0}-z_{0}\right)=0\;$$

This equality provides:

(A44) $$X=p_{0}\;,\;Y=q_{0}\;,\;Z=p_{0}x_{0}+q_{0}y_{0}-z_{0}\;$$

This is the Legendre transform. So in classical thermodynamics, the Legendre transform S(Q)=〈β,Q〉−Φ(β) is linked with polar reciprocal with respect to the paraboloid:

(A45) $$Q^{2}=2S(Q)\;$$

We can develop other properties of Legengre transform. Let’s $z=f(x,y)\;\mathrm{with}\;p=\frac{\partial z}{\partial x}\;\mathrm{and}\;q=\frac{\partial z}{\partial y}$ and X=p , Y=q , Z=px+qy−z the Legendre transform.

We compute the first derivative of *Z*:

(A46) $$dZ=PdX+QdY\;\mathrm{with}\;P=\frac{\partial Z}{\partial X}\;\mathrm{and}\;Q=\frac{\partial Z}{\partial Y}\;$$

(A47) $$Z=px+qy-z\Rightarrow dZ=pdx+qdy-dz+xdp+ydq\underset{\begin{array}{l} dz=pdx+qdy\\ X=p,Y=q \end{array}}{\Rightarrow }dZ=xdX+ydQ\Rightarrow P=x,Q=y\;$$

We compute the 2nd derivative of *Z*:

(A48) $$R=\frac{\partial^{2}Z}{\partial X^{2}}=\frac{\partial P}{\partial X}=\frac{\partial x}{\partial X}\;,\;T=\frac{\partial^{2}Z}{\partial X\partial Y}=\frac{\partial P}{\partial Y}=\frac{\partial Q}{\partial X}=\frac{\partial x}{\partial Y}=\frac{\partial y}{\partial X}\;,\;S=\frac{\partial^{2}Z}{\partial Y^{2}}=\frac{\partial Q}{\partial Y}=\frac{\partial y}{\partial Y}\;$$

(A49) $$\begin{array}{l} \begin{array}{c} \left\{ \begin{array}{l} dX=rdx+sdy\\ dY=sdx+tdy \end{array}\right.\\ r=\frac{\partial^{2}z}{\partial x^{2}},t=\frac{\partial^{2}z}{\partial y^{2}},s=\frac{\partial^{2}z}{\partial x\partial y} \end{array}\Rightarrow \left\{ \begin{array}{l} dx=\frac{t}{rt-s^{2}}dX-\frac{s}{rt-s^{2}}dY\\ dy=\frac{-s}{rt-s^{2}}dX+\frac{r}{rt-s^{2}}dY \end{array}\right.\\ \Rightarrow \left\{ \begin{array}{l} R=\frac{\partial x}{\partial X}=\frac{t}{rt-s^{2}}\\ S=\frac{\partial x}{\partial Y}=\frac{-s}{rt-s^{2}}\\ T=\frac{\partial y}{\partial Y}=\frac{r}{rt-s^{2}} \end{array}\right.\Rightarrow \left\{ \begin{array}{l} r=\frac{T}{RT-S^{2}}\\ s=\frac{-S}{RT-S^{2}}\\ t=\frac{R}{RT-S^{2}} \end{array}\right. \end{array}$$

The link with with contact transformations is then the following. Considering new variables *X,Y,Z* and *P,Q* the derivatives of *Z* with respect to *X* and *Y*, problem of finding in which case this five quantities could be express of *x,y,z,p* and *q* est the same problem where we look for five functions *X,Y,Z,P* and *Q* of five independant variables *x,y,z,p* and *q* satisfying the differential equation:

(A50) dZ−PdX−QdY=ρ(dz−pdx−qdy) 

where ρ is a function of *x,y,z,p* and *q*.

**Proof.** 

(A51) $$\left\{ \begin{array}{l} p=\frac{\partial z}{\partial x}\\ q=\frac{\partial z}{\partial y} \end{array}\right.\Rightarrow dz-pdx-qdy=0\Rightarrow dZ=PdX+QdY\Rightarrow \left\{ \begin{array}{l} P=\frac{\partial Z}{\partial X}\\ Q=\frac{\partial Z}{\partial Y} \end{array}\right.\;$$

and the reciprocal:

(A52) $$\rho =\frac{\partial Z}{\partial z}-P\frac{\partial X}{\partial z}-Q\frac{\partial Y}{\partial z}\;$$

Links with Ampere transformation is given then by the following developments. Let’s consider Ampere transformation:

(A53) $$\begin{array}{l} dz-pdx-qdy=d\left(z-qy\right)-pdx+ydq\\ \mathrm{Set}\;\{\begin{array}{c} Z=z-qy,X=x,Y=q\\ P=p,Q=-y \end{array}\Rightarrow dZ-pdX-QdY=dz-pdx-qdy \end{array}$$

Then ρ=1, and we have a contact transformation, also valid when Legendre transform is no longer valide (when $rt-s^{2}=0$, *p* and *q* are not independant) The link between Legendre transformation and Ampere transformation is then deduced. Legendre transform is obtained by same equality:

(A54) $$\begin{array}{l} dz-pdx-qdy=d\left(z-qy\right)-pdx+ydq\\ \mathrm{Set}\;\{\begin{array}{c} Z=z-qy,X=x,Y=q\\ P=p,Q=-y \end{array}\Rightarrow dZ-pdX-QdY=dz-pdx-qdy \end{array}$$

We can set:

(A55) $$\begin{array}{l} X=p,Y=q,Z=z-px-qy\\ P=x,Q=y \end{array}$$

□

For complementary studies on the Legendre transform, we can make reference to [99,101].

## Appendix D. Centrifuge Thermodynamics by Roger Balian Based on Classical Approach

Balian has studied the case of gas enclosed in a vessel rotating with an angular velocity ω in thermal equilibrium, and proved that the density of the gas is proportional to $e^{\frac{m\omega^{2}r^{2}}{2kT}}$, with classical approach. The density is increased at the periphery due to centrifugal effects.

Balian has computed the Boltzmann-Gibbs distribution without knowing Souriau equations (exercice 7b of [25]). Balian started by considering the constants of motion that are the energy and the component $J_{z}$ of the total angular momentum $J=\sum_{i}\left(r_{i}\times p_{i}\right)$. Balian observed that he must add to the Lagrangian parameter, given by (Planck) temperature β for energy, an additional one associated with $J_{z}$. He identifies this additional multiplier with −βω by evaluating the mean velocity at each point. He then introduced the same results also by changing the frame of reference, the Lagrangian and the Hamiltonian in the rotating frame and by writing down the canonical equilibrium in that frame. He uses the resulting distribution to find, through integration, over the momenta, an expression for the particles density as the function of the distance from the cylinder axis. The fluid carried along by the walls of the rotating vessel acquires a non-vanishing average angular momentum $\langle J_{z}\rangle$ around the axis of rotation, that is a constant of motion. In order to be able to assign to it a definite value, Balian proposed to associate with it a Lagrangian multiplier λ, in exactly the same way as we classicaly associate the multiplier β with the energy in canonical equilibrium. The average $\langle J_{z}\rangle$ will be a function of λ. The Gibbs density for rotating gas is given by Balian as:

(A56) $$D=\frac{1}{Z}e^{-\beta H-\lambda J_{z}}=\frac{1}{Z}\exp\left\{\sum_{i}\left[\frac{\beta p_{i}^{2}}{2m}+\lambda \left(x_{i}p_{y_{i}}-y_{i}p_{x_{i}}\right)\right]\right\}\;$$

With the energy and the average angular momentum given by:

(A57) $$U=-\frac{\partial \ln Z}{\partial \beta }=\frac{1}{kT}\;\mathrm{and}\;\langle J_{z}\rangle =-\frac{\partial \ln Z}{\partial \lambda }\;$$

The Lagrangian parameter λ has a mechanical nature. To identify this parameter, Balian compared microscopic and macroscopy descriptions of fluid mechanics. He described the single-particle reduced density by:

(A58) $$\begin{array}{ll} f\left(r,p\right) & \propto \exp\left\{-\frac{\beta p^{2}}{2m}-\lambda \left(xp_{y}-yp_{x}\right)\right\}\\ & =\exp\left\{-\frac{\beta }{2m}{\left(p+\frac{m}{\beta }\left[\lambda \times r\right]\right)}^{2}+\frac{m\lambda^{2}}{2\beta }\left(x^{2}+y^{2}\right)\right\} \end{array}\;\mathrm{and}\;\langle J_{z}\rangle =-\frac{\partial \ln Z}{\partial \lambda }\;$$

Whence Balian finds the velocity distribution at a point *r* to be proportional to:

(A59) $$\exp\left\{-\frac{m}{2kT}{\left(v+\frac{1}{\beta }\left[\lambda \times r\right]\right)}^{2}\right\}\;\mathrm{and}\;\langle J_{z}\rangle =-\frac{\partial \ln Z}{\partial \lambda }\;$$

The mean velocity of the fluid at the point r is equal to:

(A60) $$\langle v\rangle =-\frac{1}{\beta }\left[\lambda \times r\right]\;\mathrm{and}\;\langle J_{z}\rangle =-\frac{\partial \ln Z}{\partial \lambda }\;$$

and can be identified with the velocity [ω×r] in an uniform rotation with angular velocity ω. By comparison, Balian put $\omega =-\frac{\lambda }{\beta }$. Balian made the remarks that “*The angular momentum is imparted to the gas when the molecules collide with the rotating walls, which changes the Maxwell distribution at every point, shifting its origin. The walls play the role of an angular momentum reservoir. Their motion is characterized by a certain angular velocity, and the angular velocities* ω *of the fluid and of the walls become equal at equilibrium, exactly like the equalization of the temperature through energy exchanges*”.

Considering the invariance principle, Balian observed that the Lagrangian can be taken as remaining under any change of reference frame, because the stationary action principle is independent of the frame. Comparing the Hamiltonian in two frames for a single particle with position r′ and the velocity v′ in the rotating frame:

(A61) $$L_{1}=\frac{1}{2}mv^{2}=\frac{1}{2}m{\left(v\prime +\left[\omega \times r\prime \right]\right)}^{2}\;$$

Balian then considered the conjugate momentum of r′:

(A62) $$p\prime =\frac{\partial L_{1}}{\partial v\prime }=m\left(v\prime +\left[\omega \times r\prime \right]\right)\;$$

and the Hamiltonian in the rotating frame:

(A63) $$H_{1}\prime =\left(p\prime .v\prime \right)-L_{1}=\frac{p\prime^{2}}{2m}-\left(\omega .\left[r\prime \times p\prime \right]\right)\;$$

The Gibbs density in the rotating frame is then given by:

(A64) $$D=\frac{1}{Z}e^{-\beta H\prime }\;$$

where *H’* is the sum over *N* particles:

(A65) $$H\prime =\sum_{i=1}^{N}\left(\frac{p_{i}\prime^{2}}{2m_{i}}-\left(\omega .\left[r_{i}\prime \times p_{i}\prime \right]\right)\right)\;$$

At this step, Balian observed that to switch back to the original coordinates, p′ and [r′×p′] can be derived from p and [r×p], respectively, by means of the same change of coordinates that leads from r to r′. Balian then got:

(A66) H′=H−(ω.J) 

and identified density *D* with the earlier expression, provided λ=−βω.

Balian observed that as in the case of equilibrium of a gas in a gravitational field, the result could have obtained by a macroscopic calculation from Thermodynamics and Fluid Mechanics, using locally the perfect gas law and the balance between the forces, here centrifugal forces and pressure gradients. Balian recalled that we should fix the value of these Lagrangian multipliers by requiring that on the average the angular and linear momenta vanish. For symmetry reasons these quantities vanish at the same time as the corresponding multipliers, and we have:

(A67) 〈Jz〉=−∂lnZ∂λ=NmωR2[11−exp(−mω2R22kT)−2kTmω2R2]∼ω→012ωNmR2 

and the energy:

(A68) $$U=-\frac{\partial \ln Z}{\partial \beta }=\frac{3}{2}NkT+\frac{1}{2}\omega \langle J_{z}\rangle \;$$

Balian observed that in the change of frame, the linear momentum mv′ is no longer equal to the momentum p′ because the velocity v=p/m in the fixed frame is transformed in v′=p′/m−[ω×r′] in the rotating frame. Balian made the analogy with a particle of charge q in a magnetic field characterized by a velocity (p−qA)/m.

Balian wrote “*Whereas positions and velocities are physical quantities, momenta have a certain amount of arbitrariness which is connected with the fact that we can change the Lagrangian by adding to it a time derivative without changing the equations of motion*.” Balian gave the example in a Gallilean transformation with velocity u with the procedure where the Lagragian is assumed to be invariant $p_{i}\prime =p_{i}$ whereas $v_{i}\prime =v_{i}-u$, the Hamiltonian becomes H′=H−〈u,P〉, where P is the total momentum. Balian observed that another procedure, that better exhibits the Gallielan invariance consists in adding to the Lagrangian the ineffective term:

(A69) $$-\sum_{i}m_{i}\left(\left(v_{i}\prime .u\right)+\frac{1}{2}u^{2}\right)=\frac{d}{dt}\left(\sum_{i}m_{i}\left(\frac{1}{2}u^{2}t-\left(r.u\right)\right)\right)\;$$

When we change coordinates $\left(r_{i},v_{i}\right)$ to $\left(r_{i}\prime ,v_{i}\prime \right)$, the momentum which is conjugate to $r_{i}\prime$ is $p_{i}\prime \prime =p_{i}-m_{i}u=m_{i}v_{i}^{\prime }$ and not $p_{i}\prime =p_{i}$ and the Hamiltonian $H\prime \prime =H-\left(u.P\right)+\frac{1}{2}Mu^{2}$ has in terms of the $p_{i}^{\prime \prime }$ exactly the same form as H in terms of the $p_{i}$.

Balian presented these argues to be regarded as a microscopic justification of such a calculation and wrote “*As in the case of equilibrium of a gas in a gravitational field, we could have obtained the result by a macroscopic calculation from thermodynamics and fluid mechanics, using locally the perfect gas laws and the balance between the forces, here centrifugal forces and pressure gradients*”.

Balian observed that usually no conditions are unquired about the Lagrangian multipliers for dynamical constants of motion sur as the angular or the linear momentum. Balian proposes to fix the values of these multipliers by requiring that on the average the angular and linear momenta vanish. Balian observed that for symmetry reasons, these quantities vanish at the same time as the corresponding multipliers, and we have:

(A70) $$\begin{array}{ll} \langle J_{z}\rangle & =-\frac{\partial \ln Z}{\partial \lambda }=Nm\omega R^{2}\left[\frac{1}{1-e^{-m\omega^{2}R^{2}/2KT}}-\frac{2kT}{m\omega^{2}R^{2}}\right]\\ & \underset{\omega \to 0}{∼}\frac{1}{2}\omega NmR^{2} \end{array}$$

The angular momentum $\langle J_{z}\rangle$ is to lowest order in ω the same as for the rotation of a cylinder with uniform density, which has a moment of inertia equal to $\frac{1}{2}NmR^{2}$. The energy contains a contribution due to the motion, and is given by:

(A71) $$\langle J_{z}\rangle =-\frac{\partial \ln Z}{\partial \beta }=\frac{3}{2}NkT+\frac{1}{2}\omega \langle J_{z}\rangle \;$$

The entropy also depends on the rotational velocity, but only to order $\omega^{4}$. It decreases with ω, as the rotation produces changes in density which increase the spatial order.

## Appendix E. Proof of Convergence for Poly-Symplectic Model Based on Souriau Proof

Jean-Marie Souriau has given the following definition:

**Definition A5.** **[Souriau Generalized Temperature Definition]**
*Let* G *a Lie group acting on a symplectic Manifold* (M,ω) *by an Hamiltonian action* Γ:G×M→M *,* g *is Lie algebra and* $J:M\to g^{*}$ *a moment map of the action, a* ***generalized temperature*** *is an element* β∈g *such that the integral:*

(A72) $$\int_{M}e^{-\langle \beta ,J\rangle }d\lambda_{\omega }\;$$

*is normally convergent.*

Normal convergence means that there exist an open neighborhood Vfrom β to g, and a function $f:M\to {ℜ}^{+}$integrable on Mrelative to Liouville measure $\lambda_{\omega }$, such that:

(A73) $$\int_{M}e^{-\langle \beta ,J\rangle }d\lambda_{\omega }\;$$

Lebesgue theorem on dominated convergence gives the proof.

Jean-Marie Souriau then introduced the following proposition:

**Proposition A3.** **[Souriau Differentiability Proposition]**
*Consider* Ω *, a non-empty set of generalized temperatures,* Ω *is a convex open set of Lie algebra* g *that doesn’t depend on the choice of the choice of the moment map* J *associated with the Hamiltonian action. The partition function* I:Ω↔ℜ *given by* $I_{0}(\beta )=\int_{M}e^{-\langle \beta ,J\rangle }d\lambda_{\omega }$ *is infinitely differentiable on* Ω *. Its nth differentiation is given by the tensorial integral:*

(A74) $$I_{n}(\beta )=\int_{M}J^{⊗n}e^{-\langle \beta ,J\rangle }d\lambda_{\omega }\;$$

*and is normally convergent.*

Let

- $\beta_{0},\beta_{1}\in \Omega$
- $V_{0},V_{1}$ neighborhoods respectively of $\beta_{0},\beta_{1}$
- $f_{0},f_{1}$ positive integrable function on Msuch that:
  (A75) $$\left\{ \begin{array}{l} e^{-\langle \beta_{0}\prime ,J\rangle }\leq f_{0},\;\mathrm{if}\;\beta_{0}\prime \in V_{0}\\ e^{-\langle \beta_{1}\prime ,J\rangle }\leq f_{1},\;\mathrm{if}\;\beta_{1}\prime \in V_{1} \end{array}\right.\;$$

$\forall \lambda \in \left[0,1\right],V_{\lambda }=\left\{\left(1-\lambda \right)\beta_{0}\prime +\lambda \beta_{1}\prime /\beta_{0}\prime \in V_{0},\beta_{1}\prime \in V_{1}\right\}$ is a neighborhood of $\beta_{\lambda }$ given by $\beta_{\lambda }=\left(1-\lambda \right)\beta_{0}+\lambda \beta_{1}$, and the function $f_{\lambda }=\left(1-\lambda \right)f_{0}+\lambda f_{1}$ is integrable on M and $e^{-\langle \beta_{\lambda }\prime ,J\rangle }\leq f_{\lambda },\forall \beta_{\lambda }\prime \in V_{\lambda }$. Then $\beta_{\lambda }\in \Omega$ proving that Ω is convex.

*n-*th differential of $e^{-\langle \beta ,J\rangle }$ is given:

(A76) $$D^{n}\left(e^{-\langle \beta ,J\rangle }\right)={\left(-1\right)}^{n}J^{⊗n}e^{-\langle \beta ,J\rangle }\;$$

Selecting a norm on Lie algebra g, and considering Sup Norm on space L(g,ℜ)of n-multilinear forms on g. We can deduce on $g^{*}$ and on ${\left[g^{*}\right]}^{⊗n}$ a norm of multi-linear map:

(A77) $$\left\|J^{⊗n}\right\|=\underset{\beta }{Sup}\left|\langle \beta ,J\rangle \right|\;$$

Let:

(A78) $$\beta \in \Omega ,\varepsilon >0\;\mathrm{and}\;e^{-\langle \beta ,J\rangle }\leq f,\;\mathrm{if}\;\beta \prime \in g\;\mathrm{and}\;\left\|\beta \prime -\beta \right\|\leq \varepsilon \;$$

Let $\beta \prime \prime \in g\;\mathrm{and}\;\left\|\beta \prime \prime -\beta \right\|\leq \frac{\varepsilon }{2}$, for all X∈g and ‖X‖=1, then:

(A79) $$\left\|\langle X,J\rangle \right\|\leq \frac{2n}{\varepsilon }e^{\frac{\varepsilon }{2n}\left\|\langle X,J\rangle \right\|}\Rightarrow {\left\|\langle X,J\rangle \right\|}^{n}e^{-\langle \beta \prime \prime ,J\rangle }\leq {\left(\frac{2n}{\varepsilon }\right)}^{n}e^{-\langle \beta \prime \prime \pm \frac{\varepsilon }{2}X,J\rangle }\;$$

The last relation is established by considering:

(A80) ∀α∈ℜ,∀n∈ℵ,|2αn|n≤|2sh(αn)|n=|eαn−e−αn|n=|∑p=0n(−1)pCnpe−[−1+2pn]α| 

If we select X∈g and α=〈X,J〉:

(A81) |2n|ne−〈β,J〉|〈X,J〉|n≤|∑p=0n(−1)pCnpe−〈β−[2pn−1]X〉| 

(A82) $$e^{-\langle \beta ,J\rangle }\leq f\Rightarrow e^{-\langle \beta ,J\rangle }{\left|\langle X,J\rangle \right|}^{n}\leq n^{n}f\;,\;\mathrm{if}\;\left\|\beta -\beta_{0}\right\|\leq \frac{\varepsilon }{2},\left\|V\right\|\leq \frac{\varepsilon }{2}\;$$

For X unitary, and by setting $X=J\frac{\varepsilon }{2}$

(A83) $${\left\|\langle X,J\rangle \right\|}^{n}e^{-\langle \beta ,J\rangle }\leq {\left(\frac{2n}{\varepsilon }\right)}^{n}f\;$$

In ${\left\|\langle X,J\rangle \right\|}^{n}e^{-\langle \beta \prime \prime ,J\rangle }\leq {\left(\frac{2n}{\varepsilon }\right)}^{n}e^{-\langle \beta \prime \prime \pm \frac{\varepsilon }{2}X,J\rangle }$, the sign ± is selected such that 〈±εX,J〉≥0.

As $\left\|\beta -\beta \prime \prime \pm \frac{\varepsilon }{2}X\right\|\leq \varepsilon$, the final result is deduced:

(A84) $$\left\|D^{n}\left(e^{-\langle \beta \prime \prime ,J\rangle }\right)\right\|\leq {\left[\frac{2n}{\varepsilon }\right]}^{n}f\Rightarrow \left\|J^{⊗n}e^{-\langle \beta \prime \prime ,J\rangle }\right\|\leq {\left[\frac{2n}{\varepsilon }\right]}^{n}f\;$$

It proves that the n-differential of $e^{-\langle \beta ,J\rangle }$ is normally integrable on M with respect to Liouville measure, the partition function is infinitely differentiable on Ω.

By considering the taylor expansion of exponential function:

(A85) $$e^{\alpha }-1-\alpha =\frac{\alpha^{2}}{2}e^{\lambda \alpha },\lambda \in \left[0,1\right]\;$$

From which, we deduce that:

(A86) $$e^{-\langle \beta -X,J\rangle }J^{⊗n}-e^{-\langle \beta ,J\rangle }J^{⊗n}-e^{-\langle \beta ,J\rangle }J^{⊗n+1}(X)=\frac{1}{2}e^{\langle \beta -\lambda X,J\rangle }J^{⊗n+2}(X)(X)\;$$

where T(X) means the contraction of a covariant tensor with vector X. Then:

(A87) $$\left\|J^{⊗n+2}e^{-\langle \beta ,J\rangle }\right\|\leq {\left[\frac{2\left(n+2\right)}{\varepsilon }\right]}^{n+2}f\Rightarrow \frac{1}{2}e^{\langle \beta -\lambda X,J\rangle }J^{⊗n+2}(X)(X)\leq \frac{1}{2}{\left[\frac{2\left(n+2\right)}{\varepsilon }\right]}^{n+2}f{\left\|X\right\|}^{2}\;$$

By integration on V and using $\int_{V}f.Vol=a<+\infty$, we obtain:

(A88) $$\left\|I_{n}\left(\beta -X\right)-I_{n}(\beta )-I_{n+1}(\beta )\right\|\leq \frac{a}{2}{\left[\frac{2\left(n+2\right)}{\varepsilon }\right]}^{n+2}{\left\|X\right\|}^{2}\;\mathrm{if}\;\beta \in >B\left(\beta_{0},\frac{\varepsilon }{4}\right)\;\mathrm{and}\;\left\|X\right\|\leq \frac{\varepsilon }{4}\;$$

It proves that the function $I_{n}:\beta \in g\to ℜ$ is continuous and derivable in a neighborhood of $\beta_{0}$, and its derivative is given $I_{n+1}$. Then $I_{0}$ is an infinite derivable function with $I_{n}$ as nth derivable.

These demonstrations can be extended for poly-symplectic model of Souriau Lie groups Thermodynamic by considering the polysymplectic partition function:

(A89) $$I_{0}^{poly}=\int_{M}e^{-\sum_{k=1}^{n}\langle \beta_{k},J^{⊗k}(\xi )\rangle }d\lambda_{\omega }\;$$

and its *n*-th derivatices given by:

(A90) $$I_{n,i}^{poly}=\frac{\partial^{n}I_{0}}{\partial \beta_{i}^{n}}=\int_{M}J^{⊗nk}e^{-\sum_{k=1}^{n}\langle \beta_{k},J^{⊗k}\rangle }d\lambda_{\omega }\;$$

where $J^{⊗k}=J⊗\overset{\left(k\right)}{J}\ldots ⊗J\;$ is defined as a tensorial product.

## Appendix F. Relativistic Souriau Thermodynamics of Continua

We will summarize in this appendix the Souriau relativistic thermodynamics of fluids. This Souriau model about relativistic thermodynamics of continua will give a solution to Duhem’s general thermodynanics: *Nous avons fait de la dynamique un cas particulier de la thermodynamique, une Science qui embrasse dans des principes communs tous les changements d’état des corps, aussi bien les changements de lieu que les changements de qualités physiques* “. (In English: *We made dynamics a special case of thermodynamics, and science that embraces common principles in all changes of state bodies, changes of places as well as changes in physical qualities”*.)

The objective is not to make a survey of all literature on this topic. We give this model to compare Souriau’s approaches related to invariance and symmetries in thermodynamics. I think that this is the first time that Souriau relativistic model is presented in English. My objective is to underline that Souriau has replaced the geometric temperature of “Lie groups thermodynamics”, where the temperature is an element of Lie algebra, by a temperature that is defined as a killing vector. I also underline, that in both models, Souriau was motivated to search solutions where the “Legendre transform” structure is preserved between Massieu thermodynamics potentials.

Kinematics is defined by the vector field Θ and the measurement of number of molecules: using two state functions, Souriau has built a (thermo-)dynamic according to the two principles: conservation of the Noetherian quantities attached to the Poincaré group, positive Entropy production. Such a dissipative fluid has movements in which the entropy production is nil; Θ is then a killing vector; the equations of motion fully integrate; Souriau found in particular the results of kinetic theory at equilibrium. This method can be used to study perfect fluids; Souriau recover the classic Lichnerowicz results; moreover, we can build, even in the non-isentropic case, an space-time 2-Form Ω which is Integral invariant (in the sense of Cartan-Poincaré) of the temperature vector Θ; this provides a generalization of Helmholtz’s theorem. In weakly dissipative movements, naturally occur the two viscosity coefficients, as well as the thermal conductivity coefficient; they are accompanied by two other coefficients that may be measurable on actual fluids.

Jean-Marie Sourias has first considered the kinematics of a relativistic simple fluid, considering the following space-time vectors field by temperature vector X↦Θ with:

(A91) $$\Theta =U\varepsilon \left\{ \begin{array}{l} U:\mathrm{Unitary}\;\mathrm{quadri}-\mathrm{vector}\\ \varepsilon =\frac{1}{T}>0\;(\mathrm{Boltzman}\;k=1) \end{array}\right.\;$$

Θ generates a group with a parameter of diffeomorphisms of space-time *E*_4_; the group’s orbits (the current lines of the fluid) form an abstract space *V*_3_ (has a manifold structure of dimension 3, characterized by the fact that the following projection is a restricted submersion:

(A92) $$X\in E_{4}\mapsto x\in V_{3}\;$$

Let the metric tensor *g* Lie derivative (for the vector field $X\in E_{4}\mapsto \Theta$):

(A93) $$\left\{ \begin{array}{l} \gamma =\frac{1}{2}\delta_{L}g\\ \delta X=\Theta \end{array}\right.\;$$

The Killing formula gives the symmetric tensor:

(A94) $$\gamma_{\lambda \mu }=\frac{1}{2}\left[\partial_{\lambda }\Theta_{\mu }+\partial_{\mu }\Theta_{\lambda }\right]\;$$

Let consider positive density n of quotient manifold $V_{3}$:

(A95) $$x\in V_{3}\mapsto n\;$$

Integral of n on $V_{3}$ gives the number of molecules. Its reciprocal image by projection is defined by:

(A96) $$X\in E_{4}\mapsto N\;$$

Particules conservation is given by:

(A97) $$\partial_{\lambda }N^{\lambda }=0\;\mathrm{with}\;N=Un\;$$

Direction of U or Θ defines a foliation of space-time *E_4_*. Leaves are current lines solutions of:

(A98) $$\frac{dX}{ds_{c}}=U\;$$

We illustrate in Figure A3, the Souriau’s midel of Thermodynamics of continua.

Thermodynamic 1st principle in this model is given by:

(A99) $$\partial_{\lambda }T^{\lambda \mu }=0\;\mathrm{with}\;T^{\lambda \mu }=T^{\mu \lambda }\;$$

The energy-momentum density tensor $T^{\lambda \mu }$ has been built by Souriau using the kinematic quantities, such as to verify the second principle.

**Lemma A1** **[Souriau Lemma].** *Let*(n,ε)↦ζ*a differentiable function, then there is a symmetric tensor*${\overset{⌢}{T}}^{\lambda \mu }$ such that:

(A100) $$\partial_{\lambda }\left[N^{\lambda }\zeta \right]=-{\overset{⌢}{T}}^{\lambda \mu }\gamma_{\lambda \mu }\;\mathrm{with}\;\Theta =U\varepsilon \;\mathrm{et}\;N=Un\;$$

(A101) $${\overset{⌢}{T}}^{\lambda \mu }=\frac{n^{2}}{\varepsilon }\frac{\partial \zeta }{\partial n}\left[g^{\lambda \mu }-U^{\lambda }U^{\mu }\right]-n\frac{\partial \zeta }{\partial \varepsilon }U^{\lambda }U^{\mu }\;$$

We assume that there exist φ=φ(n,Θ,γ) such this function is convex and energy-momentum density are given by:

(A102) $$T^{\lambda \mu }=\frac{\partial \varphi }{\partial \gamma_{\lambda \mu }}\;$$

If we assume that $\left\{\gamma_{\lambda \mu }=0\right\}\Rightarrow \left\{T^{\lambda \mu }={\overset{⌢}{T}}^{\lambda \mu }\right\}$ then the following vector has a positive divergence:

(A103) $$S^{\lambda }=N^{\lambda }\zeta +T^{\lambda \mu }\Theta_{\mu }\;$$

The Thermodynamic 2nd principle is given by:

(A104) $$\partial_{\lambda }S^{\lambda }\geq 0\;$$

Proof is given by:

(A105) $$\partial_{\lambda }S^{\lambda }=\left[T^{\lambda \mu }-{\overset{⌢}{T}}^{\lambda \mu }\right]\gamma_{\lambda \mu }\;\partial_{\lambda }S^{\lambda }=\left\{\varphi \left(\gamma \right)-\varphi \left(0\right)-{\overset{⌢}{T}}^{\lambda \mu }\gamma_{\lambda \mu }\right\}+\left\{\varphi \left(0\right)-\varphi \left(\gamma \right)-T^{\lambda \mu }\left(-\gamma_{\lambda \mu }\right)\right\}\geq 0\;$$

$\partial_{\lambda }S^{\lambda }\geq 0$ Souriau proposed to define the dynamics of the fluid by means of the two functions ζ and φ which give at each point the energy tensor $T^{\lambda \mu }$ and the entropy flux $S^{\lambda }$ by following formulas. These functions being determined, we have 5 equations to determine the 5 variables $\left(n,\Theta^{\lambda }\right)$ and, moreover, the $S^{\lambda }$; $\partial_{\lambda }S^{\lambda }\geq 0$ will express the 2nd principle.

(A106) $$\left\{ \begin{array}{l} T^{\lambda \mu }=\frac{\partial \varphi \left(n,\Theta ,\gamma \right)}{\partial \gamma_{\lambda \mu }}\\ S^{\lambda }=N^{\lambda }\zeta \left(n,\varepsilon \right)+T^{\lambda \mu }\Theta_{\mu }\;\mathrm{with}\;\Theta =U\varepsilon \;\mathrm{and}\;N=Un\\ \gamma_{\lambda \mu }=\frac{1}{2}\left[\partial_{\lambda }\Theta_{\mu }+\partial_{\mu }\Theta_{\lambda }\right]\\ \partial_{\lambda }T^{\lambda \mu }=0\;\mathrm{and}\;\partial_{\lambda }N^{\lambda }=0 \end{array}\right.\;$$

Souriau has then considered the case of non-dissipative movements. If φ is strictly convex for variable γ then:

(A017) $$\partial_{\lambda }S^{\lambda }=0\Leftrightarrow \gamma_{\lambda \mu }=0\Leftrightarrow \Theta \;\mathrm{infinitesimal}\;\mathrm{isometry}\;$$

For non-dissipative solution of movement equations, Θ is a Killing vector, associated to an element of Lie algebra of Poincaré group:

(A108) $$\Theta =\left[\begin{array}{cc} \Lambda & \Gamma \\ 0 & 0 \end{array}\right]\;$$

with:

(A109) $$\begin{array}{cc} \Theta_{\lambda }=\Lambda_{\lambda \mu }X^{\mu }+\Gamma_{\lambda } & \left(\Lambda_{\lambda \mu }+\Lambda_{\mu \lambda }=0\right)\\ \Theta =U\varepsilon & \end{array}\}\Rightarrow U^{\lambda },\varepsilon \;$$

The equations of motion integrate through an arbitrary constant:

(A110) $$\zeta +\frac{\partial \zeta }{\partial n}n=Cste\Rightarrow n\;$$

Thermodynamics constants are the following:

- specific molecular volume:
  (A111) u=1n 
- specific mass:
  (A112) $$\rho =-n\frac{\partial \zeta }{\partial \varepsilon }=-\frac{1}{u}\frac{\partial \zeta }{\partial \varepsilon }\;$$
- pressure:
  (A113) $$\;\rho =-\frac{n^{2}}{\varepsilon }\frac{\partial \zeta }{\partial n}=-\frac{1}{\varepsilon }\frac{\partial \zeta }{\partial u}\;$$

In case of a nill entropy production:

(A114) $$\begin{array}{l} \partial_{\lambda }S^{\lambda }=0\Rightarrow \left\{ \begin{array}{l} \gamma =0\\ \Theta =\Lambda .X+\Gamma \end{array}\right.\Rightarrow \left\{ \begin{array}{l} U^{\lambda }\partial_{\lambda }\varepsilon =0\Rightarrow \exists \varepsilon ,x\in V_{3}\mapsto \varepsilon \\ \partial_{\lambda }U^{\lambda }=0\Rightarrow \left[\partial_{\lambda }N^{\lambda }=0\Rightarrow U^{\lambda }\partial_{\lambda }n=0\right]\Rightarrow \exists n,x\in V_{3}\mapsto n\\ \varepsilon U^{\lambda }\partial_{\lambda }U_{\mu }+\partial_{\mu }\varepsilon =0 \end{array}\right.\\ \Rightarrow \mathrm{variable}\;n\;\mathrm{and}\;\varepsilon \;\mathrm{are}\;\mathrm{constant}\;\mathrm{on}\;\mathrm{current}\;\mathrm{lines}\; \end{array}$$

We can also deduce the following equations:

(A115) $$\left\{ \begin{array}{l} \Theta =\Lambda .X+\Gamma \\ \partial_{\lambda }N^{\lambda }=0 \end{array}\right.\Rightarrow U^{\lambda }\partial_{\lambda }\left[\frac{n^{2}}{\varepsilon }\frac{\partial \zeta }{\partial n}\right]=0\;\mathrm{and}\;U^{\lambda }\partial_{\lambda }\left[n\frac{\partial \zeta }{\partial n}\right]=0\;$$

From tensor computation, Souriau has computed the energy-momentum density currents:

(A116) $$\begin{array}{l} \partial_{\lambda }N^{\lambda }=0\Rightarrow \partial_{\lambda }\left[N^{\lambda }\zeta \right]=N^{\lambda }\partial_{\lambda }\zeta =U^{\lambda }n\left[\frac{\partial \zeta }{\partial n}\partial_{\lambda }n+\frac{\partial \zeta }{\partial \varepsilon }\partial_{\lambda }\varepsilon \right]\\ \gamma_{\lambda \mu }=\frac{1}{2}\left[\partial_{\lambda }\Theta_{\mu }+\partial_{\mu }\Theta_{\lambda }\right]=\frac{\varepsilon }{2}\left[\partial_{\lambda }U_{\mu }+\partial_{\mu }U_{\lambda }\right]+\frac{1}{2}\left[U_{\lambda }\partial_{\mu }\varepsilon +U_{\mu }\partial_{\lambda }\varepsilon \right]\\ \Rightarrow g^{\lambda \mu }\gamma_{\lambda \mu }=\varepsilon \partial_{\lambda }U^{\lambda }+U^{\lambda }\partial_{\lambda }\varepsilon \end{array}$$

with the following developments:

(A117) $$\begin{array}{l} U\;\mathrm{unitary}\Rightarrow U_{\lambda }U^{\lambda }=g_{\lambda \mu }U^{\lambda }U^{\mu }=1\Rightarrow U^{\lambda }\partial_{\mu }U_{\lambda }=0\Rightarrow U^{\lambda }U^{\mu }\gamma_{\lambda \mu }=U^{\lambda }\partial_{\lambda }\varepsilon \\ \partial_{\lambda }N^{\lambda }=0\Rightarrow U^{\lambda }\partial_{\lambda }n+\partial_{\lambda }U^{\lambda }n=0\\ \Rightarrow T^{\lambda \mu }=\frac{n^{2}}{\varepsilon }\frac{\partial \zeta }{\partial n}\left[g^{\lambda \mu }-U^{\lambda }U^{\mu }\right]-n\frac{\partial \zeta }{\partial \varepsilon }U^{\lambda }U^{\mu } \end{array}$$

For this non-dissipative movement, we can prove:

(A118) $$\left\{ \begin{array}{l} U^{\lambda }\partial_{\lambda }\left[\frac{n^{2}}{\varepsilon }\frac{\partial \zeta }{\partial n}\right]=0\\ U^{\lambda }\partial_{\lambda }\left[n\frac{\partial \zeta }{\partial n}\right]=0 \end{array}\right.\mathrm{and}\left\{ \begin{array}{l} U^{\lambda }\partial_{\lambda }\varepsilon =0\\ \partial_{\lambda }U^{\lambda }=0\\ \varepsilon U^{\lambda }\partial_{\lambda }U_{\mu }+\partial_{\mu }\varepsilon =0 \end{array}\right.\;$$

(A119) $$\begin{array}{l} T^{\lambda \mu }=\frac{n^{2}}{\varepsilon }\frac{\partial \zeta }{\partial n}\left[g^{\lambda \mu }-U^{\lambda }U^{\mu }\right]-n\frac{\partial \zeta }{\partial \varepsilon }U^{\lambda }U^{\mu }\\ \Rightarrow \partial_{\lambda }T^{\lambda \mu }=g^{\lambda \mu }\left\{\partial_{\lambda }\left[\frac{n^{2}}{\varepsilon }\frac{\partial \zeta }{\partial n}\right]+\frac{\partial_{\lambda }\varepsilon }{\varepsilon }\left[\frac{n^{2}}{\varepsilon }\frac{\partial \zeta }{\partial n}+n\frac{\partial \zeta }{\partial \varepsilon }\right]\right\}=\frac{n}{\varepsilon }g^{\lambda \mu }\partial_{\lambda }\left\{n\frac{\partial \zeta }{\partial n}+\zeta \right\} \end{array}$$

(A120) $$\Rightarrow \left\{ \begin{array}{l} \partial_{\lambda }T^{\lambda \mu }=0\\ \partial_{\lambda }N^{\lambda }=0 \end{array}\right.\mathrm{integrable}\;\mathrm{on}\left\{ \begin{array}{l} n\;\mathrm{constant}\;\mathrm{on}\;\mathrm{current}\;\mathrm{lines}\\ n\frac{\partial \zeta }{\partial n}+\zeta \;\mathrm{constant}\;\mathrm{in}\;\mathrm{space}-\mathrm{time} \end{array}\right.\;$$

Souriau has proved that the entropy vector preserves the Legendre transform:

(A121) $$\begin{array}{l} \left\{ \begin{array}{l} S^{\lambda }=N^{\lambda }\zeta +T^{\lambda \mu }\Theta_{\mu }\\ T^{\lambda \mu }=\frac{n^{2}}{\varepsilon }\frac{\partial \zeta }{\partial n}\left[g^{\lambda \mu }-U^{\lambda }U^{\mu }\right]-n\frac{\partial \zeta }{\partial \varepsilon }U^{\lambda }U^{\mu }\\ \Theta =U\varepsilon \;\mathrm{and}\;N=Un \end{array}\right.\Rightarrow S^{\lambda }=N^{\lambda }\left[\zeta -\varepsilon \frac{\partial \zeta }{\partial \varepsilon }\right]\\ S^{\lambda }=N^{\lambda }s\Rightarrow s=\zeta -\varepsilon \frac{\partial \zeta }{\partial \varepsilon } \end{array}$$

with the entropy per molecule:

(A122) s=ζ+ρuε 

ζ is the Massieu potential (Massieu charcateristic function):

(A123) $$\begin{array}{l} \zeta =-\frac{F}{T}=-\frac{u\rho -Ts}{T}\;\mathrm{with}\;F:\;\mathrm{Helmoltz}\;\mathrm{Free}\;\mathrm{Energy}\\ \zeta +\frac{\partial \zeta }{\partial n}n=-\frac{G}{T}=-\frac{F+pu}{T}\;\mathrm{with}\;G:\;\mathrm{Free}\;\mathrm{Gibbs}-\mathrm{Duhem}\;\mathrm{Energy} \end{array}$$

The link with Souriau 2-form and Poincaré-Cartan integral invariant is given by the following developments. Consider the 1-form given by enthalpy:

(A124) $$H_{\lambda }=hU_{\lambda }\;\mathrm{with}\;h=\frac{p+\rho }{n}=u\left[p+\rho \right]\;$$

Its 2-form given by exterior differentiation

(A125) $$\Omega_{\lambda \mu }=\partial_{\lambda }H_{\mu }-\partial_{\mu }H_{\lambda }\;$$

Movement’s equation are replaced by:

(A126) $$\left\{ \begin{array}{l} \partial_{\lambda }N^{\lambda }=0\\ \partial_{\lambda }T^{\lambda \mu }=0 \end{array}\right.\Rightarrow \left\{ \begin{array}{l} \partial_{\lambda }N^{\lambda }=0\\ \Omega_{\lambda \mu }\Theta^{\mu }+\partial_{\lambda }s=0 \end{array}\right.\;$$

Ω is a Poincaré-Cartan integral invariant of the field:

(A127) $$\begin{array}{l} \Omega_{\lambda \mu }\Theta^{\mu }+\partial_{\lambda }s=0\Rightarrow \left\{ \begin{array}{l} \delta s=0\\ \delta_{L}\Omega =0 \end{array}\right.\;\mathrm{for}\;\delta X=\Theta \\ \mathrm{if}\;\partial_{\lambda }s=0\;(\mathrm{isentropic}\;\mathrm{movment})\;\Rightarrow \Theta \in \ker\left(\Omega \right) \end{array}$$

Souriau has then considered weakly dissipative movements. If we cannot know φ=φ(n,Θ,γ), it can be approximated by 2nd order development in γ variable:

(A128) $$\varphi =\varphi_{0}+{\overset{⌢}{T}}^{\lambda \mu }\gamma_{\lambda \mu }+\frac{1}{2}C^{\lambda \mu ,vq}\gamma_{\lambda \mu }\gamma_{vq}\Rightarrow T^{\lambda \mu }=T^{\lambda \mu }=\frac{\partial \varphi }{\partial \gamma_{\lambda \mu }}={\overset{⌢}{T}}^{\lambda \mu }+C^{\lambda \mu ,vq}\gamma_{vq}\;$$

Entropy production is given by:

(A129) $$\begin{array}{l} \partial_{\lambda }S^{\lambda }=\left[T^{\lambda \mu }-{\overset{⌢}{T}}^{\lambda \mu }\right]\gamma_{\lambda \mu }=C^{\lambda \mu ,vq}\gamma_{\lambda \mu }\gamma_{vq}\\ \mathrm{Onsager}\;\mathrm{Reciprocity}\Rightarrow C^{\lambda \mu ,vq}=C^{vq,\lambda \mu } \end{array}$$

55 coefficients of Transport coefficients $C^{\lambda \mu ,vq}$ are reduced to 5 coefficients (by fluid symetries and Onsager reciprocity): *A, B, C, E & F*.

Souriau then obtained relativistic (Fourier) equation of heat. Let us consider the constraints tensor:

(A130) $$\begin{array}{l} \tau_{jk}=-T_{jk}=\delta_{jk}\left[-p+\lambda_{vis}\partial_{l}v^{l}-B\frac{\partial \varepsilon }{\partial t}\right]+\mu_{vis}\left[\partial_{j}v_{k}+\partial_{k}v_{j}\right]\\ \left(j,k=1,2,3\;\mathrm{and}\;v_{j}\;\mathrm{speed},\;\mathrm{zero}\;\mathrm{at}\;\mathrm{the}\;\mathrm{point}\;\mathrm{considered}\right) \end{array}$$

With the equations given by:

- Heat Flux:
  (A131) $$T^{j0}={\left\{F\left[\vec{grad\varepsilon -\varepsilon \frac{\partial \vec{v}}{\partial t}}\right]\right\}}^{j}\;$$
- Specific Mass-Energy:
  (A132) $$T^{00}=\rho +C\frac{\partial \varepsilon }{\partial t}-B\varepsilon div\left(\vec{v}\right)\;$$
  with:
  λvis=[A−2E3]ε, μvis=Eε, ε=1T and Thermo-conductivity:FT2 

Variables *A*, *B*, *C*, *E* & *F* are functions of ε and n, and convexity of φ induces:

(A133) $$A>0,C>0,E>0,F>0,\left|B\right|<\sqrt{AC}\;$$
